# Dual‐*Sabatier* Optima: How Reaction Mechanism Determines Activity Volcano Map of Dual‐Atom Catalysts for Oxygen Reduction Reaction

**DOI:** 10.1002/anie.8386838

**Published:** 2026-04-27

**Authors:** Jin Liu, Hao Li, Haoxiang Xu, Daojian Cheng

**Affiliations:** ^1^ State Key Laboratory of Organic‐Inorganic Composites Beijing University of Chemical Technology Beijing China; ^2^ Beijing Key Laboratory of Intelligent Design and Manufacturing for Hydrogen Energy Materials Beijing University of Chemical Technology Beijing China; ^3^ Advanced Institute for Materials Research (WPI‐AIMR) Tohoku University Sendai Japan; ^4^ Deep Intelligence Experiment Technology (Beijing) Co., Ltd Beijing China

**Keywords:** data‐driven discovery, dissociation pathway, dual‐atom catalysts (DACs), oxygen reduction reaction (ORR), *Sabatier* principle

## Abstract

Dual‐atom catalysts (DACs) have demonstrated superior potential in the oxygen reduction reaction (ORR). However, the single‐peak activity volcano derived from classical associative mechanism is contrast to the large‐scale experimental data from the Digital Catalysis Platform (*DigCat*). Herein, we studied ORR over 200 DACs from thermodynamic and kinetic perspectives, and found that the dissociative mechanism is generally dominant for DACs. By integrating potential‐related microkinetic modeling and machine learning (ML)‐derived interpretable structural descriptors, we discovered a dual‐*Sabatier* optima volcano map against Δ*G*(OH*) (or structural descriptors), which was rigorously validated against available experimental data. Dual‐*Sabatier* optima stem from the rate‐determining step of dissociative mechanism switching among three elementary reactions (O_2_ dissociation → 2OH protonation → OH protonation), which can be extended across DACs containing transition metal, metal‐like, and non‐metal elements as center atoms. It opens a brand‐new perspective for rational design of DACs and atomically dispersed catalysts for other reactions beyond ORR, of which the dominant reaction mechanism may be different from single‐atom catalysts (SACs) and lead to diverse activity volcano maps. **Most importantly, this work illustrates that new phenomenon can be identified from “old experimental data” under a large data scale, with the help of theoretical simulations integrated with interpretable ML**.

## Graphical abstract

Dual‐atom catalysts (DACs) for oxygen reduction reaction (ORR), where the dissociative mechanism dominates, show a dual‐*Sabatier* optima volcano map, diverging from the classical single‐peak volcano of the associative mechanism. Potential‐dependent microkinetic modeling and interpretable machine learning rationalize experimental data and provide design guidelines for high‐performance DACs.

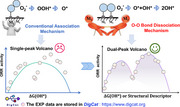

## 1 Introduction

Guided by the current consensus in catalytic chemistry and energy materials research [1, 2], noble metal‐based catalysts remain the indispensable cathodic heart of energy conversion devices [3, 4, 5], such as fuel cells and electrolyzers, owing to their peerless catalytic activity toward the electrochemical reaction. Commercial benchmarks typically necessitate substantial noble metal loadings and rely on the high cost of noble metal like Pt (∼$36 084/kg) to sustain high power‐output efficiencies [6]—fundamentally restricts its large‐scale sustainable application [7]. In this context, atomically dispersed catalysts (ADCs) have gained prominence [8]. By anchoring metal active centers as isolated atoms onto carbonaceous supports [9], ADCs achieve the theoretical limit of atom utilization and enable precise modulation of electronic structures. Notably, dual‐atom catalysts (M_1_M_2_‐N‐C DACs) represent the vanguard of this field, demonstrating catalytic performance superior to traditional single‐atom catalysts (M_1_‐N‐C SACs) across a spectrum of critical electrochemical processes [10], including oxygen reduction reaction (ORR) [11], hydrogen evolution reaction (HER) [12], oxygen evolution reaction (OER) [13], CO_2_ reduction reaction (CO_2_RR) [14], nitrate reduction reaction (NO_3_RR) [15], and electrosynthesis of organic molecules [16]. Unlike the single site in SACs, DACs contain two adjacent metal atoms (different or identical) as active centers, which not only form strong interactions with the support individually but also interact with each other [17]. Additionally, the dual‐metal sites can simultaneously or in steps adsorb multiple reactant molecules or different parts of one molecule [18], providing spatially adjacent and functionally complementary active sites, thereby facilitating multi‐step catalysis in complex reactions [19]. This synergy effect can precisely regulate the electronic structure and coordination environment of the active site, optimize the adsorption/activation ability of reactants, and effectively promote the multi‐electron transfer process [20, 22].

**FIGURE 1:**
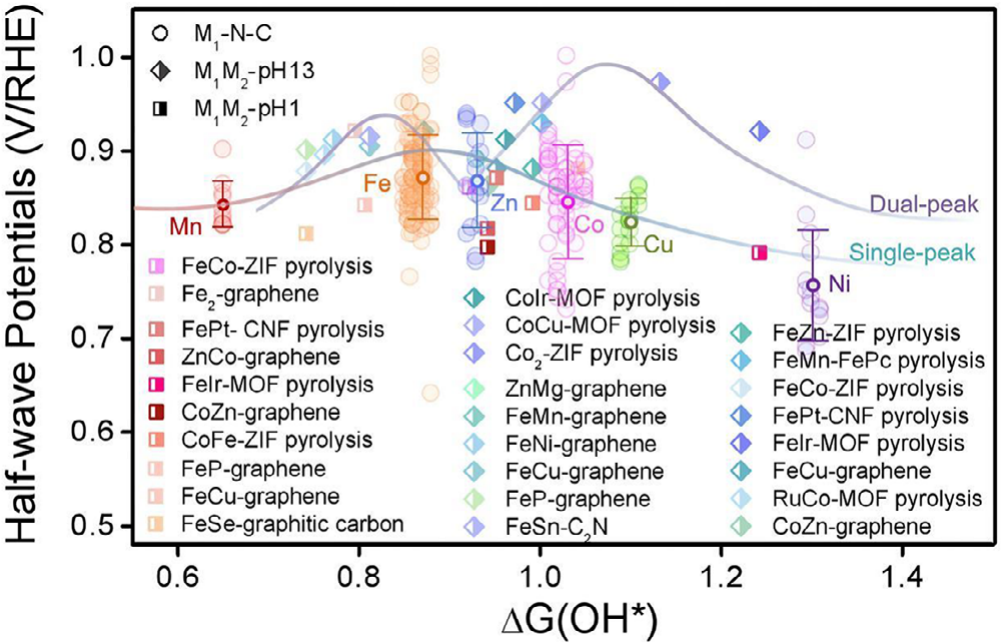
Comparison of experimental ORR performance between SACs and DACs. Experimental data indicate that ORR volcano plots for SACs and DACs exhibit a notable difference. The ORR volcano demonstrates a unique transition from a single‐peak for SACs to a double‐peak pattern for DACs. (The above data are available in the *DigCat* database: https://www.digcat.org. Note that the ORR activities of the MnFe, FeCo, and Fe_2_ systems represent averaged values from multiple reported measurements. Detailed values can be found in Tables S1 and S2). To help readers more intuitively compare the activity trends between SACs and DACs, we have presented them separately in Figures S18 and S19, and further conducted a quantitative goodness‐of‐fit analysis to compare the descriptive power of the single‐peak and dual‐peak volcano models for the experimental data.

Many experimental tests and theoretical studies have been devoted to elucidating the electrocatalytic mechanism and outstanding activity of DACs. As for ORR, the current understanding on the superior ORR performance of DACs to SACs were still based on single‐site associative mechanism (O_2_→OOH*→O*→OH*→H_2_O) [23‐25], where only one of metal sites (M_1_) in DACs serves as active center, while the others (M_2_) are spectators modifying the electronic configuration of M_1_ [26], thus enhancing the intrinsic activity of the M_1_ site [27]. Besides, theoretical or high‐throughput screening for DACs aided by density functional theory (DFT) calculations, or the interpretation of experimental results, were similar to those for SACs and Pt‐based alloys. They were still based on the single‐peak ORR activity volcano map conforming to the *Sabatier* principle [28, 29, 30], that is, moderate adsorption of oxygen intermediates (such as OH*) has the optimal activity [31, 32, 33]. **However, the single‐peak type ORR activity volcano plot derived from the classical associative mechanism is significantly inconsistent with the statistical results of the experimental data**. As shown in Figure 1, the experimental ORR activities of over 100 M–N–C SACs and metal‐containing complexes are summarized in our *DigCat* database (the Digital Catalysis Platform at https://www.digcat.org) [34]. The statistical average of half‐wave potential of Mn, Fe, Zn, Co, Cu, and Ni SACs have a single‐peak volcano relationship with OH* adsorption free energy. However, M_1_M_2_–N–C DACs with carbon substrates (e.g., graphene/carbon nanotubes (CNT), ZIF, MOF) show an evolution toward a dual‐peak volcano map. To rationalize this deviation, we introduce two new concepts. The first is “dual‐Sabatier optima”, which denotes the presence of two separate peaks in catalytic activity along a descriptor axis, originating from the concurrent operation of associative and dissociative ORR pathways on DACs. The second is “moderate adsorption trap”, referring to a descriptor window where the adsorption free energies of oxygen‐containing intermediates (e.g., OH*, O*, OOH*) are neither too weak (leading to low coverage) nor too strong (causing site poisoning), but moderate enough to balance surface speciation and unlock the dissociative mechanism. As shown below, these concepts fundamentally reshape the volcano landscape for DACs. What's more, the superior kinetic behavior of DACs to SACs can't be rationalized through the classical *Sabatier* principle or associative mechanism. ***Therefore, the underlying origin of this enhanced performance warrants further mechanistic study***.

In recent experimental works, some characterization results suggested that DACs catalyze ORR *via* mechanisms other than the associative mechanism [35, 36]. Wang et al. [36]. and Liu et al. [37]. observed side‐on adsorption of O_2_ by in‐situ SR‐FTIR technology and adjusted the atomic distance of DACs to induce O─O radical breakage without forming redundant OOH* intermediates, which cause the ORR to follow the dissociated ORR pathway. Our previous theoretical studies [38] have also found that, in certain dual‐atom systems (including Fe and Co), the oxygen molecule is activated via side‐on adsorption, and ORR proceeds via a dissociation mechanism, yielding higher ORR activity than the associative mechanism. Therefore, it is necessary to conduct systematic research to explore the universality of the dissociation mechanism in DACs and establish a corresponding ORR activity “volcano map” to guide the rational design of DACs.

**SCHEME 1:**
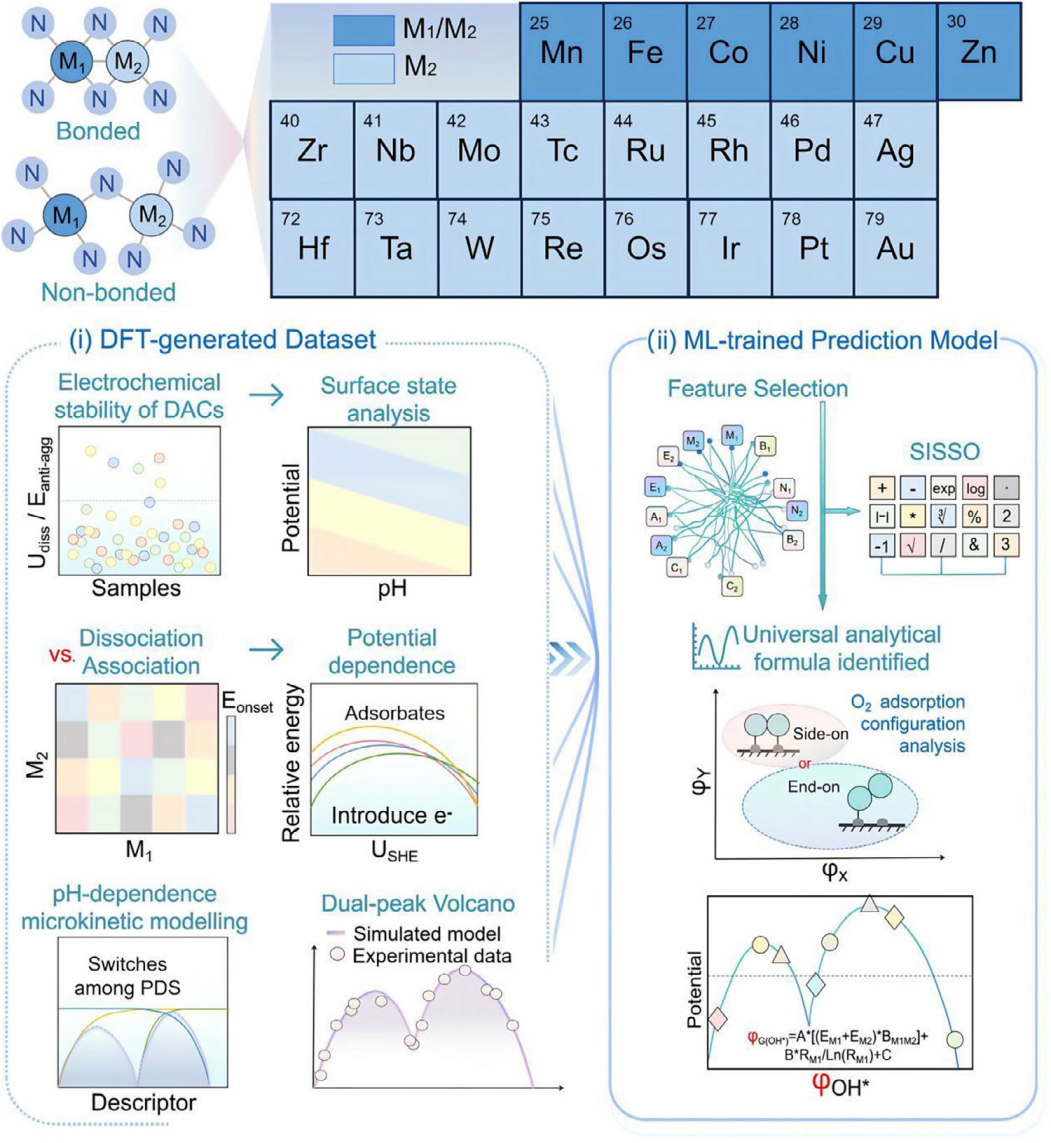
General workflow of this work, including two major parts: (i) Establish a DFT‐generated dataset, and (ii) a ML prediction model based on structural descriptors.

Herein, we constructed a series of DACs (M_1_M_2_–N–C, M_1_ = Mn, Fe, Co, Ni, Cu, and Zn; M_2_ = Zr–Au) on the two‐dimensional nitrogen‐doped graphene surface. As shown in the Scheme 1, in the first part, the ORR process over 210 types of DACs containing transition metals was systematically studied. First, DACs that could follow O_2_ side‐on adsorption were screened out for the following stability and activity analysis. Further, constant‐potential calculations were performed to explore the effects of electrode potential and pH on the free energy profile. All DACs that meet O_2_ side‐on adsorption criteria catalyze ORR via a dual‐site dissociation mechanism, with thermodynamic and kinetic superiority. The corresponding volcano map of ORR activity showed a dual‐peak pattern and rationalizes available experimental data, which is attributed to switching among three rate‐determining steps. In the second part, based on the database established in the first part through DFT calculations, a classification model using the Random Forest (RF) algorithm was developed to determine the preferred adsorption mode of O_2_ on DACs. The machine learning (ML) model was trained using the available features and DFT calculation results. A structural descriptor was further built using the Sure Independence Screening and Sparsifying Operator (SISSO) methods [39], achieving correlation with the dual‐peak ORR activity trend. The ML models above were applied to high‐throughput screening of 216 types of DACs containing metal‐like and nonmetallic elements as central atoms. 14 DACs with promising ORR activity were identified, some of which have also been reported in experimental references.

## 2 Results and Discussion

### 2.1 Identification of the O_2_ Side‐on Adsorption Mode on DACs

**FIGURE 2:**
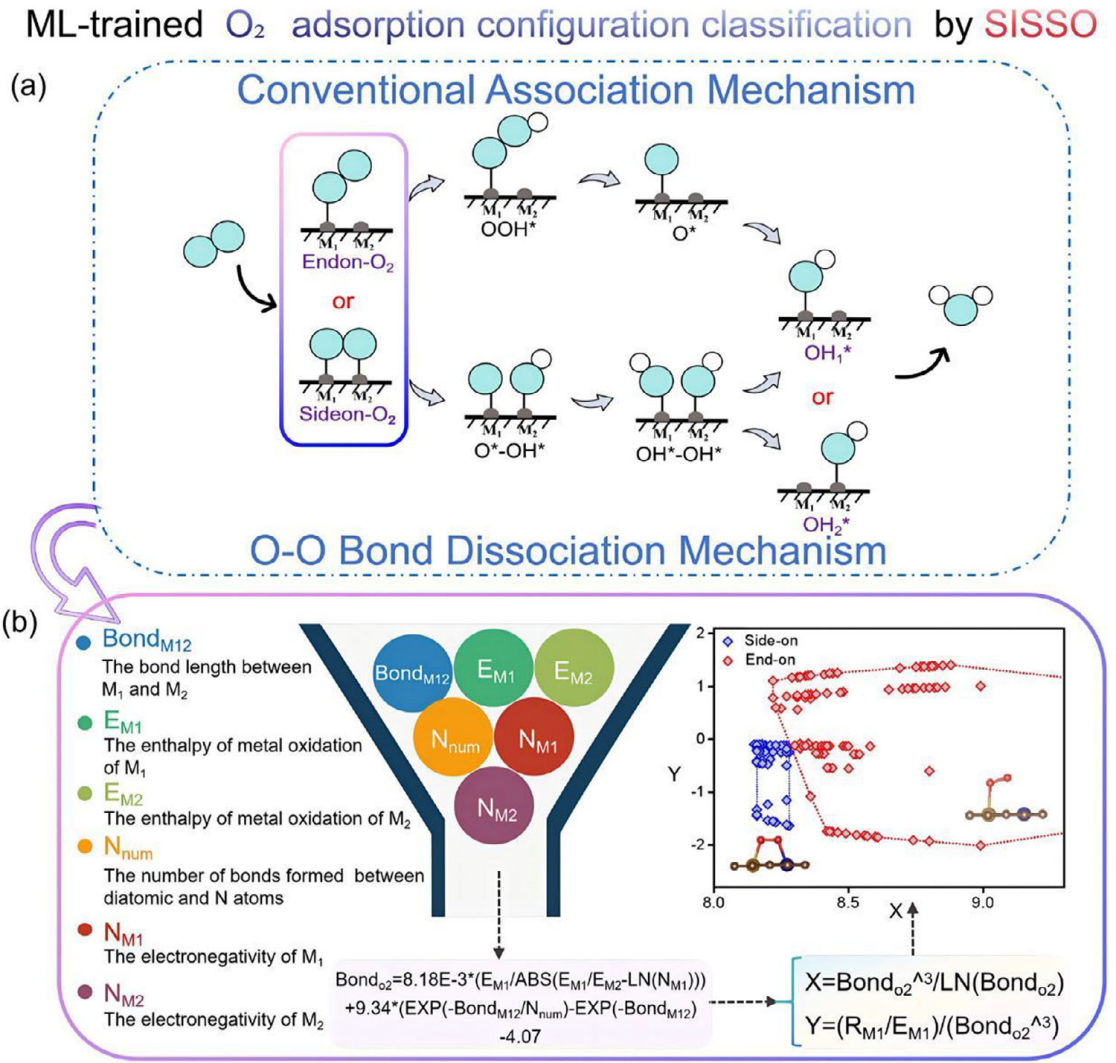
ML‐trained O_2_ adsorption configuration classification by the SISSO. (a) Reaction mechanisms map of association and dissociation. (b) Schematic diagrams of ML‐assisted classification agent for O_2_ adsorption mode on M_1_M_2_ DACs.

As shown in Figure 2a, in the traditional ORR reaction pathway with only one active center and O_2_ end‐on adsorption, the binding energies of various oxygen‐containing reaction intermediates (including OH*, O*, and OOH*) are highly correlated [40, 41], resulting in the theoretical onset potential being limited [42]. When the second active center is introduced, O_2_ can side‐on adsorb on two adjacent active sites in the DACs, and the O─O bond can be directly broken upon protonation to form the O*+OH* intermediate (without the existence of OOH*). Using FeRu‐DAC as an example, we clarify the dissociation pathway of the O─O bond breakage during the ORR process (Details referring to Note S2.1), which is feasible based on the “electron acceptation‐inversion dual‐channel” mechanism from our previous work [38]. Subsequently, during the protonation of 2OH* to generate OH* along dissociation pathway, the difficulty of desorbing OH* upon protonation at different sites of DACs varies. Compared with the single active site in the traditional association pathway, the active site of OH* protonation over DACs is optional and optimizable, thereby leading to an improvement in ORR performance. The dissociation mechanism becomes a favorable reaction mechanism by taking advantage of the dual‐site. This dual‐site coordinated O─O dissociation pathway forms the structural basis for the high activity of DACs.

As shown in Figure S2, two representative and thermodynamically stable coordination configurations of DACs were adopted in this study, in which each metal atom is 4‐coordinated to N atoms. M_1_M_2_‐1 is formed by the chemical bond between two transition metal atoms, while M_1_M_2_‐2 is formed when the two metal atoms in DACs do not form a chemical bond. The selection of these configurations was based on systematic stability screening in our previous work [38], which confirmed them as the two most stable structural types, thus representing the core interaction mechanisms of “direct bonding” and “substrate‐mediated coupling” in DACs. Moreover, various experimental characterization techniques [43, 44, 45] have also fully confirmed the stable existence of M_1_M_2_ in these two configurations on N‐doped graphene. Considering that the synthesis and application in ORR for MnM [46], FeM [47], CoM [48], NiM [49], CuM [50], and ZnM [51] have been widely reported in experimental studies, in this work, Mn, Fe, Co, Ni, Cu, and Zn were selected as the candidate materials for M_1_. Meanwhile, the metals of M_2_ are 3d, 4d, and 5d transition metals from IVB to IB group (except Tc). Finally, 210 DAC models were constructed, including M_1_M_2_‐1 with metal‐atom bond lengths of 2.306–2.735 Å and M_1_M_2_‐2 with bond lengths of 3.417–3.602 Å. The bond lengths optimized between M_1_ and M_2_ after DFT calculations are consistent with experimental measurements [35, 52]. For instance, experimental confirmation of the Mn─Fe bond length in MnFe@NC is 2.38 Å [35, 46], which aligns with our calculated value of 2.40 Å (see Table S14 for other systems).

Given that O_2_ can side‐on adsorb at adjacent sites on DACs, providing a structural precondition for the activation of the ORR dissociation mechanism, we developed an ML model. The target variable in this model is the preferred adsorption configuration of O_2_ at DACs, which is used to predict whether dissociation can occur. Additionally, designing comprehensive and appropriate features is an important step in constructing the ML model. Nine physicochemical properties of DACs and O_2_ were selected as the ML model's features. As shown in Figure 2b, the oxidation enthalpies of the metal atoms (E_M1_ and E_M2_) reflect physical properties. The electronegativity (N_M1_, N_M2_, and (N_M1_ + N_M2_)/2) represents electronic features. The atomic radii of metals (R_M1_ and R_M2_), the length of the optimized metal–metal bond (Bond_M1M2_), and the number of bonds between the two metal atoms and N (N_num_) describe the geometric characteristics of the system. All values of the 9 features and the results of 210 DFT calculations on whether O_2_ can side‐on adsorb were combined into the original dataset (Tables S6 and S7) to construct the ML model.

To ensure the rationality of the feature set, the heat map depicting the Pearson correlation coefficients between the nine features and their respective importance is presented in Figure S3a. It is evident that the correlations among most features are relatively low, and the importance of similarity is also relatively low. This suggests that the selected feature set is reasonable. Furthermore, we performed SHapley Additive exPlanation (SHAP) analysis [53, 54, 55] to assess the importance of each feature in the RF model and its contribution to model predictions (Figure S3b,c). Among these, the enthalpy of oxidation formation of the two metals (E_M1/M2_) is identified as the most significant feature, accounting for approximately 45% of the total contribution, indicating that the species of the active center atom was the prerequisite factor for the preferable adsorption mode of O─O. After validating the active center, the distance between the bimetallic (Bond_M1M2_) became the second‐most‐determined factor. To further elucidate the complex relationships between catalyst characteristics and performance, the recently developed SISSO method was applied to a preprocessed dataset. SISSO can effectively screen and construct explicit mathematical expressions with clear physical meaning from a pool of candidate descriptors, moving beyond simple numerical quantification (for details, refer to Note S2.2). Consequently, the O_2_ adsorption modes of all studied systems can be categorized into two distinct regions: side‐on and end‐on (as illustrated in Figure 2 and summarized in Table 1). It is evident that in the explicit descriptor expression, only the Bond_M1M2_ feature requires DFT calculations. Subsequently, we employed the SISSO to quantify the correlation between each feature vector (the set of nine vectors) and Bond_M1M2_, as presented in Table 1. Notably, these descriptors coincided with the two factors analyzed previously. This phenomenon indicates that both geometric and electronic effects can manipulate the Bond_M1M2_. The root mean square error (RMSE) within the model was merely 0.0057 Å (Figure S3d). By incorporating characteristics such as the enthalpy of oxidation formation of bimetallic atoms, it is feasible to bypass DFT calculations and rapidly identify whether O_2_ can undergo side‐on adsorption over DACs.

**TABLE 1:** The expression formed by atomic properties trained by SISSO to predict Bond_M1M2_ and classify the O_2_ adsorption modes_._

Training objective	Descriptor
**Bond_O2_**	**Bond_O2_** = 8.18E^−3^*(E_M1_/ABS(E_M1_/E_M2_‐LN(N_M1_))) +9.34*(EXP(‐Bond_M12_/N_num_)‐EXP(‐Bond_M12_)‐4.07
**O_2_ Side‐on adsorption**	**X** = Bond_O2_^ [3]/LN(Bond_O2_)
	**Y** = (R_M1_/E_M1_)/(Bond_O2_^ [3])

### 2.2 Thermodynamic and Kinetic Superiority of the Dissociation Pathway for ORR on DACs

Among the 210 DACs constructed, 78 of them can achieve side‐on adsorption of O_2_ at adjacent sites (Table S8). The thermodynamic and electrochemical stabilities of these models were evaluated by the anti‐aggregation energy (E_anti‐agg_) and dissolution potential (U_diss_) (Table S9), leading to the selection of 42 systems that satisfied the predefined stability criteria (for details, refer to Note S2.3). Subsequently, we analyzed the pre‐adsorption phase diagrams, which allowed us to determine the active site structure for each system under the conditions of the ORR (for additional information, see Note S2.4).

**FIGURE 3:**
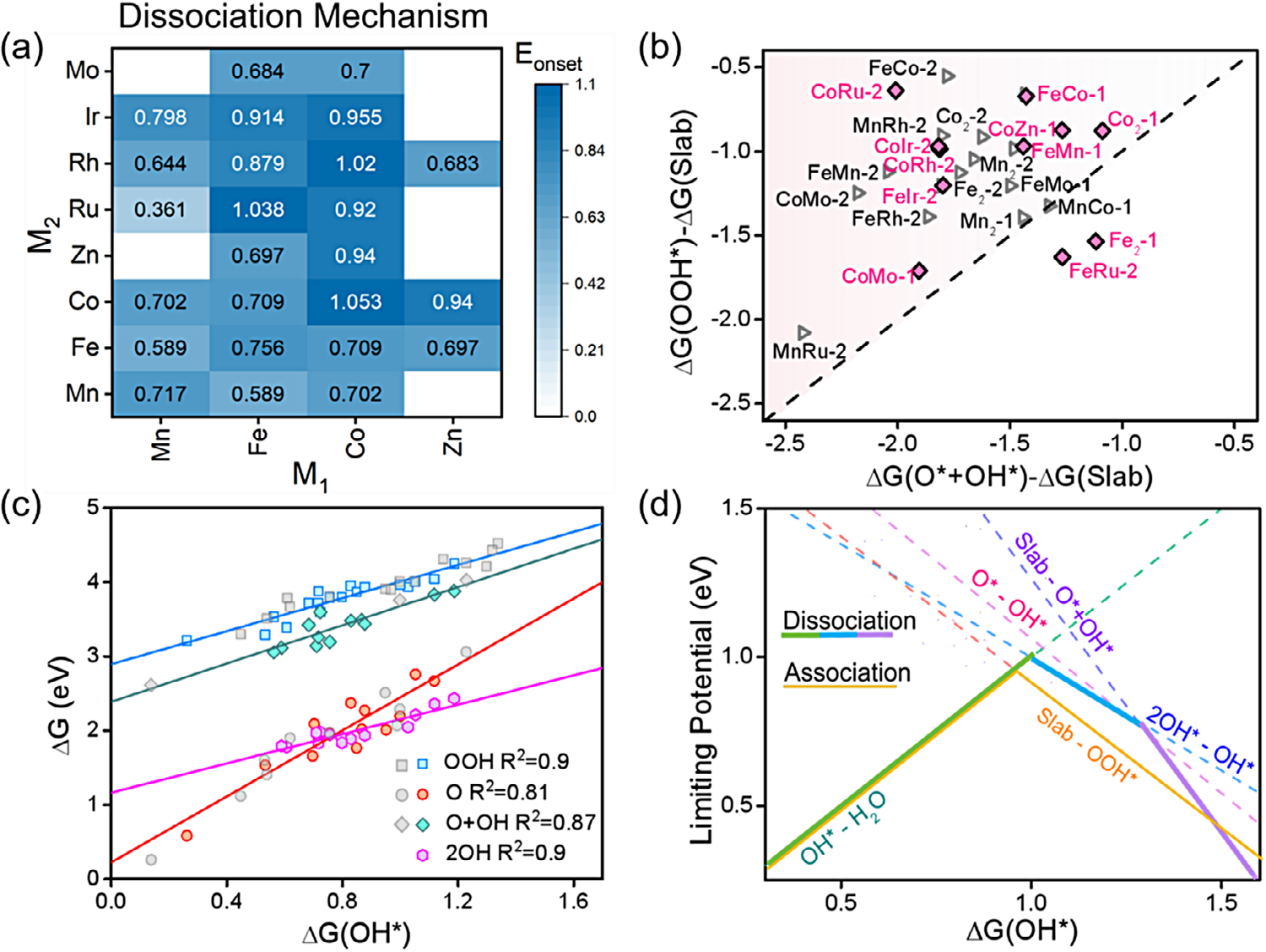
Thermodynamic analysis of the ORR process incorporating both associative and dissociative pathways. The heat map of ORR theoretical onset‐potential through (a) dissociation mechanism on M_1_M_2_‐2. (b) Gibbs free energy change (Δ*G*) of O_2_* + H^+^ + e^−^→ O*+OH* versus O_2_* + H^+^ + e^−^ → OOH*. (c) Scaling relationships among the free energy of intermediate under the association mechanism and dissociation mechanism. The data of the gray point come from Refs. [50, 51, 56, 57, 58, 59, 60]. (d) Volcano‐shaped relationship between Δ*G*(OH*) and the thermodynamic onset potential under association and dissociation mechanisms, respectively.

A systematic study was conducted on the free energy profile of stable forms of DACs (Figures S8 and S9), in which the ORR performance of the association and dissociation mechanisms was compared, clarifying the activity differences between the two mechanisms. From a thermodynamic perspective, the onset potential (E**^onset^_ORR_** = ‐max[Δ*G_i_*], i represents the step number corresponding to each elementary reaction) of ORR is limited by the step with the largest reaction free energy (Δ*G*_max_), which is also regarded as the potential‐determining step (PDS) of ORR. The smaller the free energy drop in the PDS, the more it indicates that ORR can only proceed at a lower potential. The PDS of the ORR reaction of the FeCo‐1 through dissociation mechanism is the protonation process of 2OH* (Figure S5e), with a Δ*G*_max_ of −0.92 eV, which is far lower than the association mechanism. The generation process of OOH* in the association mechanism is the PDS of ORR, and the step where the reaction free energy drops the least is only −0.67 eV. Similarly, the Δ*G*_max_ of the dissociation mechanism over the CoIr‐2 is −0.92 eV (Figure S5f), while that of the association mechanism is only Δ*G*_max_ = −0.26 eV. Therefore, the PDS of the dissociation mechanism releases more heat than that of the association mechanism, indicating that the dissociation mechanism plays a more predominant role in ORR. Other DACs also exhibited similar phenomena, as indicated by the free energy profiles (Figures S8 and S9). According to the heat map of the ORR theoretical onset‐potential in Figures 3a and Supplementary S10, a higher theoretical onset potential gradually increases from the association mechanism to the dissociation mechanism. Moreover, the atomic pairs composed of M_1_ and post‐transition metals, such as FeCo, FeMn, FeIr, etc., tend to follow the dissociation mechanism and exhibit superior ORR activity.

The dominance of the dissociation pathway stems from its distinctive reaction network and the characteristics of its intermediates. A key feature of this route is the lateral adsorption and direct cleavage of O_2_, which yields intermediate species—specifically the adsorbed pair O+OH* and subsequently 2OH*—that are not present in the conventional associative mechanism. As shown in Figure 3b, for most DACs, the formation of O+OH* releases significantly more heat than the formation of OOH*, indicating that ORR tends to follow the dissociation mechanism and produce the more stable O+OH* intermediate. Furthermore, we analyzed the linear relationships among the adsorption free energies of intermediates involved in both the dissociation and associative mechanisms (O+OH*, 2OH*, OH*, O*, and OOH*). By integrating data from prior studies (represented in gray) with our calculated results (colored), a consistent scaling relationship was established (Figure 3c). The results show that the scaling between Δ*G*(OH*) and Δ*G*(OOH*)/Δ*G*(O*) aligns well with reported trends. Notably, Δ*G*(O+OH*) is lower than Δ*G*(OOH*), further confirming that the dissociation mechanism is thermodynamically more favorable.

Meanwhile, Δ*G*(OH*) serves as a descriptor for the ORR activity trend on DACs (Figure 3d). In the associative mechanism, the protonation steps slab→OOH* (right branch) and OH*→H_2_O (left branch) constitute the PDS. In contrast, the dissociation mechanism involves three PDS: slab→O+OH*, 2OH*→OH*, and OH*→H_2_O. Here, the PDS differs between these two mechanisms. Under strong OH* adsorption (Δ*G*(OH*)≤–1.05 eV), DACs lie on the left branch of the volcano, and the PDS is OH*→H_2_O (the thick green solid line)—the same as in the associative mechanism (the fine yellow solid line). For moderate OH* adsorption (–1.05 ≤Δ*G*(OH*) ≤ –1.35 eV), 2OH*→OH* becomes the PDS, which differs from the associative route (where slab→OOH* is the PDS). In heteronuclear DACs, the reaction free energy for 2OH* protonation varies considerably due to differences in the electronic structures and coordination environments of the two metal sites, directly influencing the thermodynamics of this step. Under weak OH* adsorption (Δ*G*(OH*)≥–1.35 eV), slab→O+OH* becomes the PDS in the dissociation mechanism, again distinct from the associative mechanism (PDS: slab→OOH). These shifts in PDS from the associative to the dissociative mechanism enhance DACs in the right branch, thereby effectively increasing the E_onset_ of ORR and improving overall catalytic performance.

In addition to the thermodynamic superiority above, to further explore the kinetic feasibility of the dissociation mechanism, we identified the contribution of a dual‐site dissociation pathway in the ORR kinetics, through systematically analyzing the contribution of each pathway to the total polarization curve, as shown in Figures S11 and S12. Obviously, the association pathway (red) of each stable system contributes a little current density, while the dissociation pathway (blue) contributes a much greater degree of flux, indicating that the dual‐site dissociation pathway is dominant. To further validate our kinetic model, we simulated the polarization curve of FeRu‐2 and compared it with the experimentally reported one (Figure S21). The calculated half‐wave potential (0.82 V) and onset potential (1.0 V) agree well with the experimental values of 0.85 V and 1.0 V, respectively. The minor discrepancies are discussed in detail in Section S2.5 of the Supporting Information. This consistency confirms the reliability of our microkinetic model.

**FIGURE 4:**
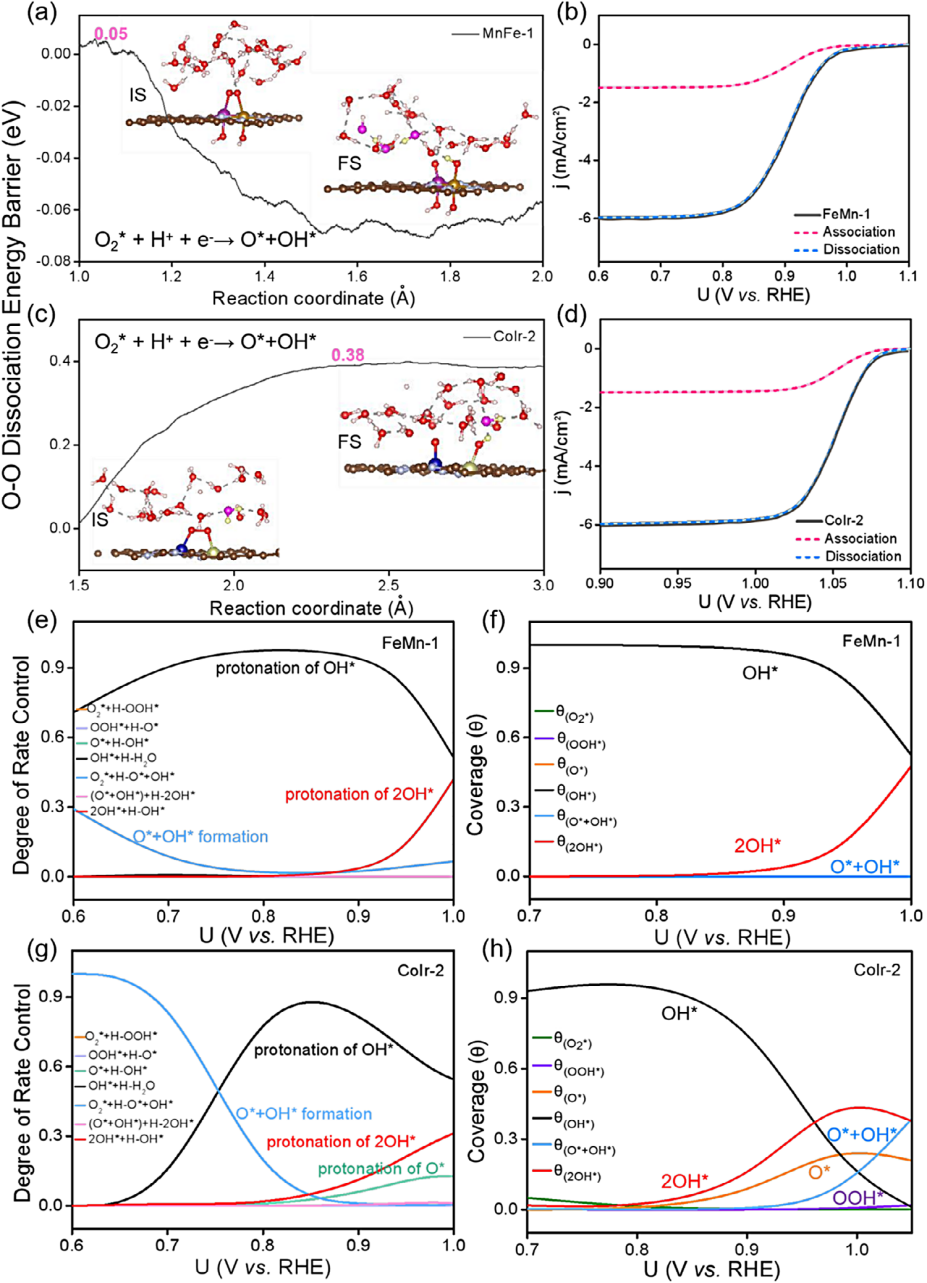
Kinetic analyses of the ORR Catalyzed by FeMn‐1 and CoIr‐2. (a), (c) Free energy profile and average energy during the O─O bond breaking process upon protonation from solution on MnFe‐1 and CoIr‐2. (b), (d) Simulated polarization curves of MnFe‐1 and CoIr‐2 DACs, as well as the contribution from the association and dissociation mechanisms with calculated O‐O dissociation barrier. (e), (g) The degree of rate control and (f), (h) coverage of intermediate for each elementary step in the reaction network as a function of output potential on MnFe‐1 and CoIr‐2 DACs. Note: Coverage and degree of rate control profiles are shown in the respective panel for each metal, and those not explicitly shown indicate zero coverage.

The rate for electrochemical reduction steps is calculated assuming an activation energy of 0.26 eV for the proton‐electron transferring step in aqueous solution, as previously suggested [61]. Furthermore, to ensure the accuracy of the calculations, taking FeMn‐1 and CoIr‐2 as examples, the constrained *ab initio* molecular dynamics (AIMD) and the climbing‐image nudged elastic band (CI‐NEB) methods were used to simulate the dissociation process of the O─O bond upon protonation. The activation energy barrier values calculated by the two methods were very close to each other (Figures S13a,b and 4a–c). When the calculated activation energy barriers by the constrained AIMD method are substituted into the microkinetic model, it was found that the polarization curve changes little and the dual‐site dissociation pathway is still dominant (Figure 4b,d), which indicates that the activation energy barriers of each elementary step to 0.26 eV do not affect the conclusion of this study.

**FIGURE 5:**
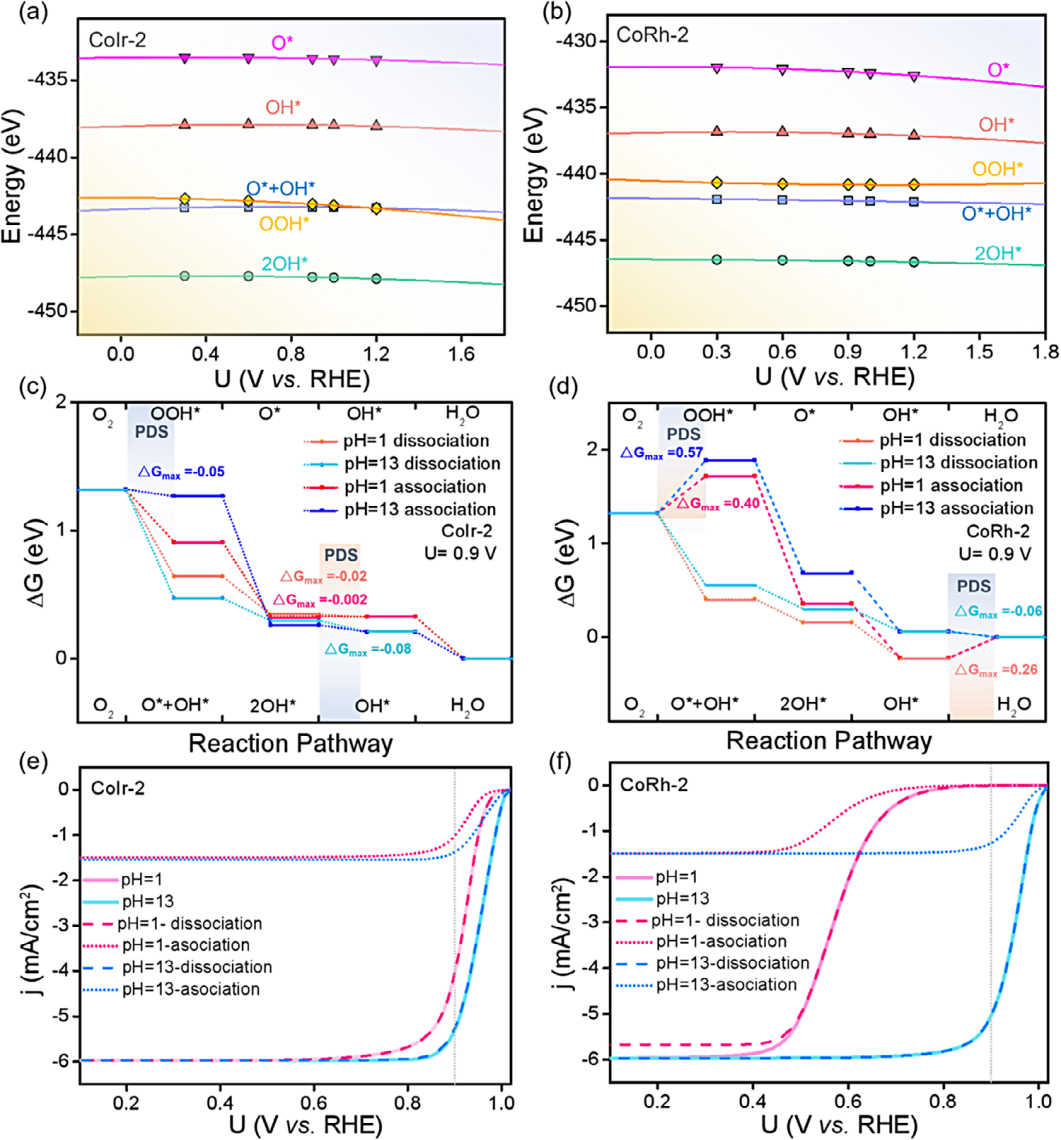
CoIr‐2 and CoRh‐2 catalyze the ORR process for pH‐dependent and potential‐dependent conditions. Variation of Gibbs free energy as a function of potential for (a) CoIr‐2 DAC and (b) CoRh‐2 DAC. Free energy diagrams of (c) CoIr‐2 DAC and (d) CoRh‐2 DAC at ORR working potentials (at 0.9 V vs. RHE) with pH = 1 and 13. Simulated polarization curves of (e) CoIr‐2 DAC and (f) CoRh‐2 DAC with pH = 1 and 13.

As previously mentioned, the protonation process of 2OH* in dissociation mechanism can selectively proceed via hydrogenation at thermodynamically more favorable sites, thereby overcoming the constraints imposed by the traditional association mechanism and potentially elevating the theoretical onset potential for ORR. However, this advantage still requires further kinetic validation. Taking the FeRu‐2 as a representative example, the adsorption free energy of OH* at the Fe site (Δ*G*(OH*‐Fe) = 0.53 eV) is considerably lower than that at the Ru site (Δ*G*(OH*‐Ru) = 1.04 eV), as illustrated in Figure 5a. Nevertheless, the AIMD simulation results reveal that the protonation energy barriers at these two sites are relatively similar (0.296 eV at the Fe site and 0.287 eV at the Ru site), as shown in Figure S14. This suggests that the protonation of OH* does not kinetically preferably occur at the thermodynamically most stable adsorption site (i.e., the Fe site). In contrast to the conventional association mechanism, where the intermediate is fixed at a specific active site, DAC enables OH* to dynamically distribute and transition between adjacent metal centers through a dual‐site dissociation mechanism. Consequently, the adsorption configuration and binding energy of OH* exhibit a degree of variability. This characteristic expands the range of feasible reaction pathways for elementary steps, allowing the protonation process to occur beyond a single specific adsorption site. As a result, the flexibility ultimately leads to improved overall ORR performance of the catalyst.

Figure 4e–h shows potential‐dependent intermediates coverage and degree of rate control for each elementary step (for details, refer to Section S1.2) for the FeMn‐1 DAC and CoIr‐2 DAC. At the common working potential (*U* = 0.9 V vs. RHE), apart from the protonation process of OH*, the 2OH* formation from the hydrogenation of O*+OH* is found to be the second rate‐limiting step for the whole ORR, indicating that ORR proceeds dominantly through the dual‐site dissociation pathway. Besides, the coverage of both O*+OH* and 2OH* intermediates, belonging to dual‐site dissociation pathway, are much larger than that of OOH* solely involved in association pathway. It also suggests a predominant contribution of the dual‐site dissociation pathway to ORR activity on FeMn‐1 and CoIr‐2 DACs. These findings indicate that the transition of the reaction pathway toward the dual‐site dissociation mechanism can be plausibly regarded as the underlying origin for the superior kinetic behavior exhibited by DACs.

### 2.3 ORR Performance Analysis Based on pH‐Dependence and Potential‐Dependence

Results from conventional constant‐charge method calculations indicate the preferability and superiority of the dissociation mechanism for ORR over DACs at the computational hydrogen electrode. To more accurately simulate the electrochemical interface and investigate the effects of pH and electrode potential on catalytic activity, constant‐potential method calculations were performed. Using the high‐performance CoRh DAC and experimentally reported CoIr DAC [62] as representative examples, pH and electrode potential effect on free energy were introduced by adjusting the work function (W_f_) of the system, as illustrated in Figures 5a,b (The specific fitting formula is shown in Tables S4 and S5). From the free energy profile in thermodynamics (Figure 5c), regardless of acidic or alkaline environments, the calculated reaction free energy of PDS under the dissociation mechanism of CoIr@NC (alkaline Δ*G*_max_ = −0.08 eV, acidic Δ*G*_max_ = −0.02 eV) is more exothermic than the corresponding values under the association mechanism (alkaline condition: −0.05 eV and acidic condition: ‐0.002 eV). From the polarization curve in kinetics, the experimentally measured ORR onset potential under alkaline conditions for CoIr@NC was 1.0 V versus RHE [62], and the theoretical onset potential of it under alkaline conditions was 0.91 V versus RHE under the associative mechanism and 0.97 V versusRHE under the dissociative mechanism (Figure 5e). **These findings demonstrate that experimental observations are aligning more closely with results predicted under the dissociative mechanism**. It is notable that under acidic conditions, the ORR performance following the dissociation mechanism is still superior to that following the association mechanism. For the CoRh‐2 DAC (Figure 5d), under realistic ORR operating conditions (*U* = 0.9 V vs. RHE), the reaction free energy of PDS calculated under the dissociation mechanism in alkaline medium was Δ*G*_max_ = −0.06 eV, and was ‐0.26 eV in acidic medium, which were significantly lower than the corresponding values under the association mechanism (0.57 eV in alkaline medium and 0.40 eV in acidic medium). The results of the microkinetic calculations (Figure 5f) also indicate that the dual‐site dissociation pathway is dominant, and its oxygen reduction reaction performance is significantly superior to that of the association mechanism.

Subsequently, we systematically analyzed the contributions of the two reaction mechanisms in acidic and alkaline media to the total polarization curve. Based on the constant potential calculation data of 10 DACs reported in the experiments [48, 51, 58, 59, 62, 63, 64, 65], a microkinetic model related to pH and electrode‐potential was constructed to simulate the ORR polarization curve (Figure S15). The results showed that under different pH conditions, the associative pathway contributes only marginally to the current density, whereas the dual‐site dissociation pathway dominates the reaction flux, suggesting its predominant role in the ORR process. Notably, we further examined the influence of the electrochemical environment on the O_2_ adsorption configuration. The results confirm (as shown in Figure S16) that the O_2_ adsorption mode—namely, whether it adopts a side‐on or end‐on configuration—remains unchanged under varying applied potentials or simulated implicit acidic/alkaline environments. This robustness indicates that the key adsorption configuration is dictated primarily by the intrinsic geometric and electronic properties of the dual‐atom sites, thereby providing a stable structural foundation for the dissociative mechanism across a wide pH range. This finding aligns with recent experimental characterization [37, 66] using in situ synchrotron radiation spectroscopy, which directly identifies the key intermediate species (Pt─O─O─Fe) without the formation of redundant OOH* intermediates during ORR in PtFe DAC. Moreover, the intrinsic turnover frequency (TOF_e‐_) at 0.9 V vs. RHE, simulated based on the dissociation mechanism, was highly consistent with the experimentally measured activity trend (Figure S17), once again verifying the applicability and reliability of the dissociation mechanism across different media. Notably, to avoid errors arising from variations in active site density, the theoretical activity in this study is compared with the experimental activity using the TOF_e‐_ at 0.9 V rather than the half‐wave potential.

### 2.4 Dual‐Peak Volcano Map for ORR Activity on DACs

**FIGURE 6:**
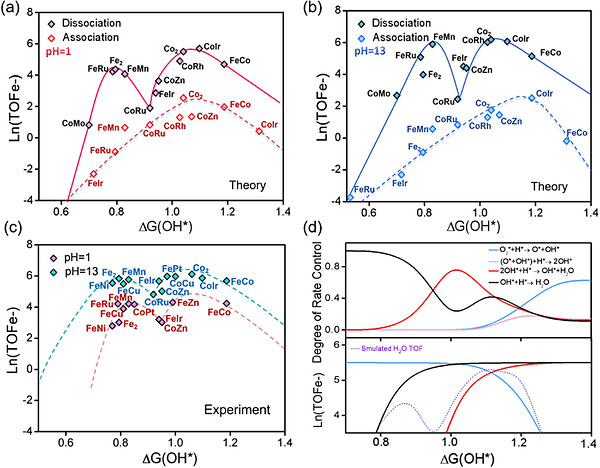
Microkinetic ORR volcano models of M_1_M_2_−N−C DACs and rate‐determining analyses. ORR activity as function of Δ*G*(OH*) contributed from dissociation and association mechanism over DACs at 0.9 V *vs*. RHE with (a) pH = 1 and (b) pH = 13 from theoretical calculation. (c) Experimental ORR activity as function of Δ*G*(OH*) over DACs at 0.9 V vs. RHE with pH = 1 and pH = 13 from Ref [35, 46, 62, 63, 64, 65], with all relevant experimental values and sources provided in Tables S1 and S2. (d) The degree of rate control for each elementary step of dissociation mechanism as a function of Δ*G*(OH*) on DACs at 0.9 V vs. RHE. And Rate‐determining analyses of the ORR process through dissociation mechanism. Dashed lines indicate the ORR activity solved by PDS analysis with the rate limited by the O_2_ protonation dissociation (blue), one of 2OH* protonation (red) and OH* protonation (black).

Different from the ORR volcano plots of DACs that only consider the association mechanism (showing a single peak, i.e., the *Sabatier* optimum), the ORR volcano plots with the introduction of the dissociation mechanism evolve into double peaks under both alkaline and acidic conditions. In Figure 6a,b, the dual‐peak volcano represents the ORR TOF_e‐_ following the dual‐site dissociation mechanism as a function of Δ*G*(OH*), at 0.9 V versus RHE. Meanwhile, the ORR activity trend for the traditional association pathway remains a single‐peak volcano spanned by Δ*G*(OH*). The dual‐peak volcanoes show superior ORR activity to single‐peak volcanoes within all region of Δ*G*(OH*) (Figure 6a,b), which again proves that dual‐site dissociation mechanism is more preponderant than the association mechanism for DACs.

Besides, moderate Δ*G*(OH*) corresponds to optimal ORR activity in single‐peak volcanoes based on association pathway, known as the *Sabatier* Optima. However, when the dissociation mechanism is included, a “moderate adsorption strength trap”, or “dual‐*Sabatier* optima behaviors” appears. This creates two optimal ranges of Δ*G*(OH*) for ORR activity through the dissociation pathway. To be noted, the dual‐peak volcano behaviors derived from theoretical calculation remarkably align with previous experimental observations in both acidic and alkaline solutions (Figure 6c). As shown in Figure 6a–c, the established dual‐peak model systematically captures the performance variations among FeM and CoM DACs. Both Figure 6c and Tables S1 and S2 confirm that catalysts located within the “performance trap” near Δ*G*(OH*) ∼ 0.9 eV (e.g., FeIr (pH = 1), CoRu (pH = 13)) exhibit experimentally measured ORR activities substantially lower than those near the optimal regions of the dual‐peak volcano (e.g., FeMn, CoIr). This trend cannot be explained by conventional *Sabatier* optimum theory based on the association mechanism. These DACs (e.g., FeIr (pH = 1), CoRu (pH = 13)) have moderate OH* adsorption strength, but their ORR performance is much lower than other FeM and CoM DACs with stronger or weaker OH adsorption. Traditional *Sabatier*’s optimal theory of the association mechanism does not account for this. Notably, the two optimal Δ*G*(OH*) in the dual‐peak volcano plots (Figure 6a,b) shift slightly between acidic (pH 1) and alkaline (pH 13) conditions: the left peak rises from ∼0.79 eV (pH = 1) to ∼0.83 eV (pH = 13), while the right peak shifts from ∼1.1 eV (pH = 1) to ∼1.05 eV (pH = 13). This trend reflects the pH‐dependent free energies of proton—electron transfer intermediates (see Section S1.3, Equation (2)). Our microkinetic model, based on the RHE, captures this through the Nernst equation. Despite minor shifts in optimal adsorption strengths, the dual‐peak volcano shape persists across pH, supporting the universality and robustness of the dissociation mechanism and the dual‐*Sabatier* optimum.

To better understand why a dual‐peak volcano appears in the dual‐site dissociation mechanism, we analyzed reaction rates for ORR steps on M_1_M_2_–N–C DACs. We also found that two switches in the PDS among three elementary reactions (OH* protonation → 2OH* protonation → O_2_ hydrogenation dissociation) occur as Δ*G*(OH*) increases, according to the degree of rate control, shown in upper row of Figure 6d. And then we calculated TOF solved by with the rate successively limited by artificially prescribed PDSs, namely OH* protonation (black), one of 2OH* protonation (red) and O_2_ protonation dissociation (blue), according to PDS analysis above, and obtained dashed lines indicating the integrated TOF (under row of Figure 6d). As shown in Figure 6, first, strong hydroxyl binding (< 0.85 eV) makes OH* removal as the PDS, so that higher activity of OH* protonation determines higher integrated TOF. As Δ*G*(OH*) becomes more positive (0.85 ∼ 0.11 eV), PDS gradually transfers from OH* protonation to 2OH* protonation (2OH*→OH*), and the activity of 2OH* protonation (2OH*→OH*) shows a monotonically increasing trend starting from an extremely low TOF. It makes the integrated TOF present an inverted volcano trend (first decrease and then increase) in this region (Δ*G*(OH*) = 0.85 ∼ 0.11 eV). When OH* adsorption is weaker (> 1.1 eV), O_2_ protonation dissociation step to form O+OH* becomes the another PDS, and the damped activity of O_2_ protonation dissociation step led to the decreasing integrated TOF. Therefore, when ORR activity solved by rate successively limited by the artificially prescribed OH* protonation, one of 2OH* protonation and O_2_ protonation dissociation, the integrated TOF as a function of Δ*G*(OH*) indeed presents a dual‐peak volcano. Typically, a *Sabatier* volcano plot shows a single peak from a single switch between two PDSs. For DACs, two switches among three PDSs during dissociation create a dual‐peak volcano. This behavior forms a dual‐*Sabatier* optimum.

### 2.5 Structural Descriptor‐Based Prediction Model Through SISSO

**FIGURE 7:**
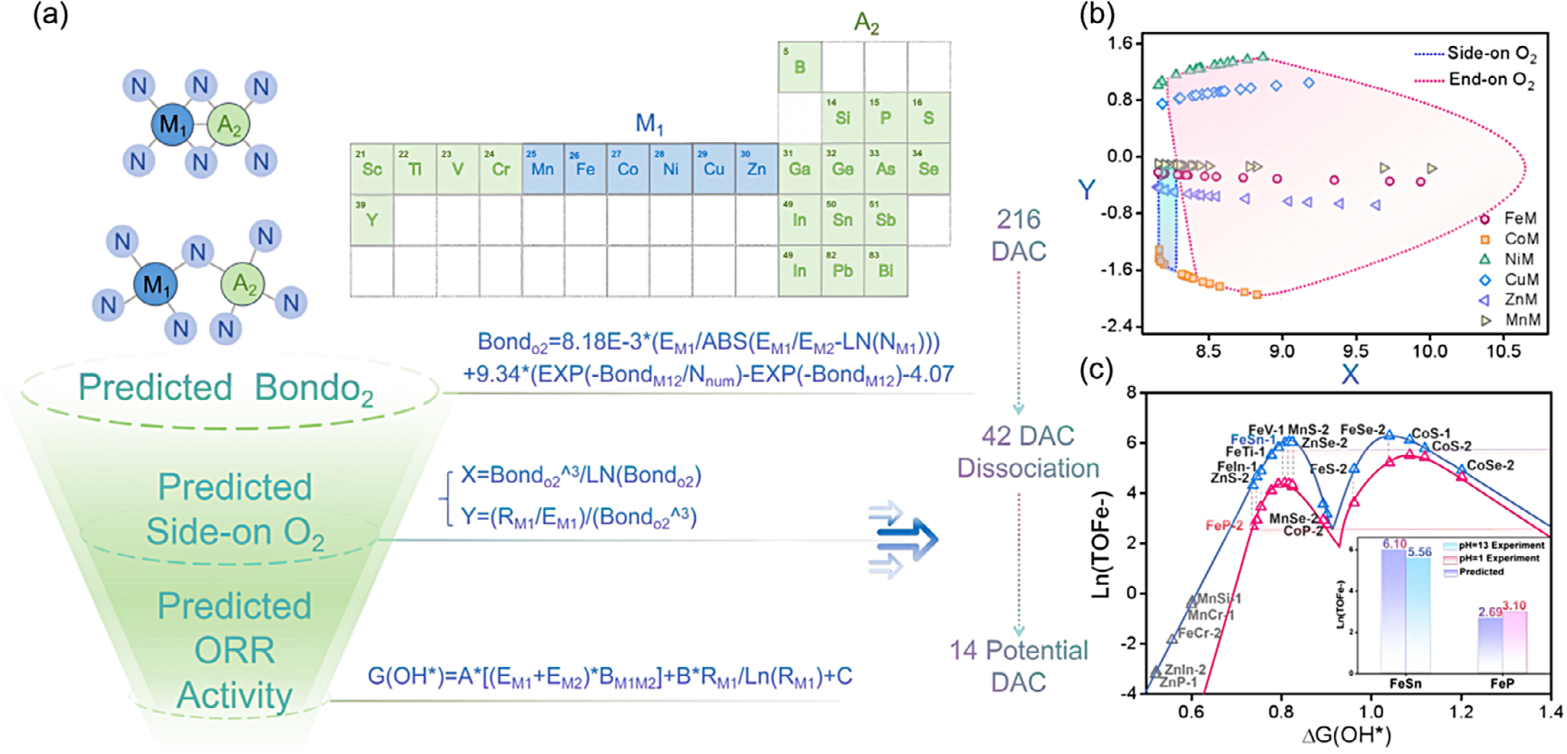
ML model prediction for potential M1A2‐DACs. (a) Schematic diagrams of structural descriptor‐based ORR catalytic performance predictions for DACs containing early transition metal, nonmetal, or metalloid elements. (b) Classification diagram of O_2_ adsorption modes for DACs containing early transition metal, nonmetal or metalloid element. (c) Predicted ORR activity contributed from dissociation mechanism over DACs at 0.9 V vs. RHE with pH = 13 (blue) and pH = 1 (pink) through structural‐descriptor‐based Δ*G*(OH*).

Based on the established model above, we systematically evaluated the ORR performance of 216 types of M_1_A_2_–DAC (M_1_ = Mn, Fe, Co, Ni, Cu, Zn; A_2_ includes non‐metallic elements B, Si, P, and S, as well as semi‐metallic elements Ga, Ge, As, Se, In, Sn, Sb, Pb, and Bi) (Figure 7a). We employed the side‐on O_2_ adsorption mode with the formation of bridging O‐O bonds on adjacent metal sites, as a criterion for the feasibility of dissociation pathways, and used this as a preliminary screening standard (Figure 7b). To further establish quantitative structure‐activity relationships, we employed interpretable ML methods and conducted feature importance analyses across multiple feature vectors to identify key features affecting ORR activity. Based on the SISSO algorithm, we established a structural descriptor with specific mathematical expression to corelate Δ*G*(OH*) (Figure 7a). The RMSE across all components in the model was as low as 0.0068 eV, demonstrating excellent predictive accuracy. Based on the obtained structural descriptor, we constructed a rapid screening model for ORR activity based on the intrinsic characteristics of bimetallic centers.

Among the 216 candidate DACs, 42 were identified as capable of proceeding via the ORR dissociation mechanism and selected for the following activity prediction. To verify the predictive ability of the model, we compared the key results with the reported experimental data. Zhang et al. [67]. reported that under alkaline conditions, the ORR TOF_e‐_ of FeSn DAC at 0.9 V vs. RHE was 5.56 s^−1^, which was highly consistent with the predicted value of 6.10 s^−1^ in our study (Figure 7c). Similarly, Chen et al. [68]. measured the TOF of FeP DAC in acidic medium to be 3.1 s^−1^, which was also consistent with the predicted value of 2.69 s^−1^ by this model (Figure 7c). The high consistency between the predicted values and the experimental data fully demonstrates the excellent reliability of this structural descriptor‐based screening model, considering the dissociation mechanism. FeP‐2 in acidic conditions and FeSn‐1 in alkaline conditions were used as the screening criterion since they have been experimentally reported. A total of 14 unreported DACs were ultimately screened out with promising ORR activity and were presented in Tables S12 and S13. The above results indicate that the structural descriptor‐based dual‐peak volcano prediction model, which considers the dissociation mechanism, is reliable and can provide an effective tool for large‐scale, rapid screening of high‐performance ORR DACs.

## 3 Conclusion

In this work, we move beyond identifying a dominant reaction pathway to **unveil a new chemical insight: the emergence of “dual‐*Sabatier* optima” in the ORR over DACs**. This phenomenon is evident in large‐scale experimental data. **Notably, it contradicts the classical single‐peak volcano derived from associative mechanisms. It represents a fundamental departure from the conventional *Sabatier* principle for atomically dispersed systems**.

Our integrated approach uses constant‐potential DFT calculations, microkinetic modeling, and interpretable machine learning to show that DACs’ superior activity comes from a dissociative mechanism. The dual‐peak activity volcano is shaped by a dynamic switch in the PDS among three basic reactions: O_2_ dissociation, O protonation, and OH protonation — each controlled by OH* adsorption strength. This detail leads to the idea of a “moderate adsorption trap,” a framework explaining why DACs with intermediate Δ*G*(OH*) may not be best and refining design principles for multi‐atom catalysts.

The derived structure‐activity descriptors and dual‐peak model apply universally across DACs comprising transition metals, metalloids, and non‐metals, and are rigorously validated against experimental data in both acidic and alkaline media. More broadly, this work establishes that the dominant reaction mechanism—and thus the shape of the activity volcano—can fundamentally differ between single‐atom and multi‐atom catalysts. Additionally, it underscores how large‐scale data analysis, precise theory, and interpretable machine learning extract profound new insights from existing experimental knowledge. Together, these advances offer a refined blueprint for the rational design of advanced catalysts, not only for ORR but for a wide spectrum of multi‐step catalytic reactions.

## Conflicts of Interest

The authors declare no conflicts of interest.

## Supporting information



The detailed calculation methods of thermodynamic stability analysis and pre‐adsorption phase diagrams used in this work; the structures and adsorption energies of reactants, intermediates, transition states and products; the microscopic kinetic models and the comparison of predicted results with experimental results regarding polarization curves; as well as the analysis related to the O_2_* adsorption process, descriptor mining, and the reaction pathways of ORR.**Supporting File 1**: anie72390‐sup‐0001‐SuppMat.docx.

## Data Availability

The data that support the findings of this study are available from the corresponding author upon reasonable request.

## References

[1] J. Zhang , C. Zhang , Y. Zhao , et al., “Three Dimensional Few‐Layer Porous Carbon Nanosheets Towards Oxygen Reduction,” Applied Catalysis B: Environmental 211 (2017): 148–156, 10.1016/j.apcatb.2017.04.038.

[2] J. Zhang , L. Qu , G. Shi , J. Liu , J. Chen , and L. Dai , “N,P‐Codoped Carbon Networks as Efficient Metal‐Free Bifunctional Catalysts for Oxygen Reduction and Hydrogen Evolution Reactions,” Angewandte Chemie (International ed in English) 55, no. 6 (2016): 2230–2234, 10.1002/anie.201510495. 26709954

[3] C. H. Choi , M. Kim , H. C. Kwon , et al., “Tuning Selectivity of Electrochemical Reactions by Atomically Dispersed Platinum Catalyst,” Nature Communications 7 (2016): 10922, 10.1038/ncomms10922. PMC4786782 26952517

[4] R. Shen , W. Chen , Q. Peng , et al., “High‐Concentration Single Atomic Pt Sites on Hollow CuS_x_ for Selective O_2_ Reduction to H_2_O_2_ in Acid Solution,” Chemistry 5, no. 8 (2019): 2099–2110, 10.1016/j.chempr.2019.04.024.

[5] Y. Liu , X. Quan , X. Fan , H. Wang , and S. Chen , “High‐Yield Electrosynthesis of Hydrogen Peroxide From Oxygen Reduction by Hierarchically Porous Carbon,” Angewandte Chemie (International ed in English) 54, no. 23 (2015): 6837–6841, 10.1002/anie.201502396. 25892325

[6] Y. J. Wang , N. Zhao , B. Fang , H. Li , X. T. Bi , and H. Wang , “Carbon‐supported Pt‐Based Alloy Electrocatalysts for the Oxygen Reduction Reaction in Polymer Electrolyte Membrane Fuel Cells: Particle Size, Shape, and Composition Manipulation and Their Impact to Activity,” Chemical Reviews 115, no. 9 (2015): 3433–3467, 10.1021/cr500519c. 25871490

[7] H. T. Chung , D. A. Cullen , D. Higgins , et al., “Direct Atomic‐Level Insight Into the Active Sites of a High‐Performance PGM‐Free ORR Catalyst,” Science 357, no. 6350 (2017): 479–484, 10.1126/science.aan2255. 28774924

[8] Z. W. Chen , L. X. Chen , C. C. Yang , and Q. Jiang , “Atomic (Single, Double, And Triple Atoms) Catalysis: Frontiers, Opportunities, and Challenges,” Journal of Materials Chemistry A 7, no. 8 (2019): 3492–3515, 10.1039/C8TA11416A.

[9] L. Bai , Z. Duan , X. Wen , R. Si , and J. Guan , “Atomically Dispersed Manganese‐Based Catalysts for Efficient Catalysis of Oxygen Reduction Reaction,” Applied Catalysis B‐Environmental 257 (2019): 4755–4762, 10.1021/acsaem.9b00386.

[10] L. Li , K. Yi , and Y. Chen , “Breaking the Scaling Relationship Limit: From Single‐Atom to Dual‐Atom Catalysts,” Accounts of Materials Research 3 (2022): 584–596, 10.1021/accountsmr.1c00264.

[11] M. Liu , X. Wang , S. Cao , et al., “Ferredoxin‐Inspired Design of S‐Synergized Fe–Fe Dual‐Metal Center Catalysts for Enhanced Electrocatalytic Oxygen Reduction Reaction,” Advanced Materials 36, no. 19 (2024): e2309231, 10.1002/adma.202309231. 38345181

[12] C. Rong , X. Shen , Y. Wang , et al., “Electronic Structure Engineering of Single‐Atom Ru Sites via Co‐N_4_ Sites for Bifunctional pH‐Universal Water Splitting,” Advanced Materials 34, no. 21 (2022): e2110103. 35384087 10.1002/adma.202110103

[13] Z. Pei , X. F. Lu , H. Zhang , Y. Li , D. Luan , and X. W. D. Lou , “Highly Efficient Electrocatalytic Oxygen Evolution Over Atomically Dispersed Synergistic Ni/Co Dual Sites,” Angewandte Chemie (International ed in English) 61, no. 40 (2022): e202207537, 10.1002/anie.202207537. 35894631

[14] J. Ding , H. Bin Yang , X.‐L. Ma , et al., “A Tin‐Based Tandem Electrocatalyst for CO_2_ Reduction to Ethanol With 80% Selectivity,” Nature Energy 8, no. 12 (2023): 1386–1394, 10.1038/s41560-023-01389-3.

[15] Q. Li , C. Jia , Q. Wang , et al., “Rational Design of Conductive MOF‐Based Diatomic Electrocatalysts for Selective Ammonia Synthesis,” Journal of the American Chemical Society 147, no. 43 (2025): 39430–39439, 10.1021/jacs.5c11655. 41111208

[16] C. Zhao , Y. Jin , J. Yuan , et al., “Tailoring Activation Intermediates of CO_2_ Initiates C–N Coupling for Highly Selective Urea Electrosynthesis,” Journal of the American Chemical Society 147, no. 10 (2025): 8871–8880, 10.1021/jacs.5c00583. 40035438

[17] Y. Li , Y. Li , H. Sun , et al., “Current Status and Perspectives of Dual‐Atom Catalysts Towards Sustainable Energy Utilization,” Nanomicro Letters 16, no. 1 (2024): 139. 10.1007/s40820-024-01347-y PMC10904713 38421549

[18] J. Kundu , T. Bhoyar , S. Park , H. Jin , K. Lee , and S.‐I. Choi , “Recent Advances in Single‐ and Dual‐Atom Catalysts for Efficient Nitrogen Electro‐Reduction and Their Perspectives,” Advanced Powder Materials 4, no. 2 (2025): 100279, 10.1016/j.apmate.2025.100279.

[19] J. Wu , Z. Chen , K. Yang , et al., “Electric Bias‐Induced Reversible Configuration Of Single And Heteronuclear Dual‐Atom Catalysts on 1Tʹ‐MoS_2_ ,” Nature Nanotechnology 20, no. 8 (2025): 1043–1051, 10.1038/s41565-025-01934-z. 40389639

[20] D. C. Zhong , Y. C. Wang , M. Wang , and T. B. Lu , “Precise Synthesis of Dual‐Atom Catalysts for Better Understanding the Enhanced Catalytic Performance and Synergistic Mechanism,” Accounts of Chemical Research 58, no. 9 (2025): 1379–1391, 10.1021/acs.accounts.4c00855. 40207527

[21] X. Chen , H. Cheng , R. Shi , T. Zhou , T. He , and Q. Liu , “Recent Advances in Data‐Driven Design of Dual‐Atom Catalysts,” Advanced Functional Materials 36, no. 2 (2025): e10647, 10.1002/adfm.202510647.

[22] L. Liu and A. Corma , “Bimetallic Sites for Catalysis: From Binuclear Metal Sites to Bimetallic Nanoclusters and Nanoparticles,” Chemical Reviews 123, no. 8 (2023): 4855–4933, 10.1021/acs.chemrev.2c00733. 36971499 PMC10141355

[23] W. Ye , S. Chen , Y. Lin , et al., “Precisely Tuning the Number of Fe Atoms in Clusters on N‐Doped Carbon Toward Acidic Oxygen Reduction Reaction,” Chemistry 5, no. 11 (2019): 2865–2878, 10.1016/j.chempr.2019.07.020.

[24] L. Jiao , J. Zhu , Y. Zhang , et al., “Non‐Bonding Interaction of Neighboring Fe and Ni Single‐Atom Pairs on MOF‐Derived N‐Doped Carbon for Enhanced CO_2_ Electroreduction,” Journal of the American Chemical Society 143, no. 46 (2021): 19417–19424, 10.1021/jacs.1c08050. 34779627

[25] A. Kulkarni , S. Siahrostami , A. Patel , and J. K. Norskov , “Understanding Catalytic Activity Trends in the Oxygen Reduction Reaction,” Chemistry Reviews 118, no. 5 (2018): 2302–2312, 10.1021/acs.chemrev.7b00488. 29405702

[26] Y. Zhang , F. Li , S. Li , et al., “Asymmetric Dual‐Atomic Catalyst With Axial Chloride Coordination for Efficient Oxygen Reduction Reaction,” Advanced Materials 37, no. 33 (2025): e2507478, 10.1002/adma.202507478. 40437913

[27] R. Sui , B. Liu , C. Chen , et al., “Constructing Asymmetric Fe–Nb Diatomic Sites to Enhance ORR Activity and Durability,” Journal of the American Chemical Society 146, no. 38 (2024): 26442–26453, 10.1021/jacs.4c09642. 39267445

[28] B. Wei , Z. Fu , D. Legut , et al., “Rational Design of Highly Stable and Active MXene‐Based Bifunctional ORR/OER Double‐Atom Catalysts,” Advanced Materials 33, no. 40 (2021): e2102595, 10.1002/adma.202102595. 34342921

[29] J. Xu , A. Elangovan , J. Li , and B. Liu , “Graphene‐Based Dual‐Metal Sites for Oxygen Reduction Reaction: A Theoretical Study,” Journal of Physical Chemistry C 125, no. 4 (2021): 2334–2344, 10.1021/acs.jpcc.0c10617.

[30] L. Zhang , X. Guo , S. Zhang , and S. Huang , “Building up the “Genome” of Bi‐Atom Catalysts Toward Efficient HER/OER/ORR,” Journal of Materials Chemistry A 10, no. 21 (2022): 11600–11612, 10.1039/D2TA02050E.

[31] X. Zhao , X. Liu , B. Huang , P. Wang , and Y. Pei , “Hydroxyl Group Modification Improves the Electrocatalytic ORR and OER Activity of Graphene Supported Single and bi‐metal Atomic Catalysts (Ni, Co, and Fe),” Journal of Materials Chemistry A 7, no. 42 (2019): 24583–24593, 10.1039/C9TA08661G.

[32] X. Zhu , J. Yan , M. Gu , et al., “Activity Origin and Design Principles for Oxygen Reduction on Dual‐Metal‐Site Catalysts: A Combined Density Functional Theory and Machine Learning Study,” Journal of Physical Chemistry Letters 10, no. 24 (2019): 7760–7766, 10.1021/acs.jpclett.9b03392. 31786912

[33] D. Li , P. Sun , H. Xu , J. Yun , and D. Cao , “A Revised High‐Throughput Screening Model on Oxygen Reduction Reaction Over Dual Atom Catalysts Based on the Axial Pre‐Adsorption and O_2_ Adsorption,” Advanced Energy Materials 15, no. 9 (2024): 2403524, 10.1002/aenm.202403524.

[34] D. Zhang and H. Li , “Digital Catalysis Platform (DigCat): A Gateway to Big Data and AI‐Powered Innovations in Catalysis,” Chemistry Archive 1 (2024): 9lpb9, 10.26434/chemrxiv-2024-9lpb9/v3.

[35] L. Liu , J. Hu , X. Rao , et al., “Revealing the Dependence of Oxygen Reduction Mechanism and Activity on the D‐Band Center Difference of Fe‐M Bimetallic Sites,” Applied Catalysis B: Environment and Energy 384 (2026): 126191, 10.1016/j.apcatb.2025.126191.

[36] Y. Xie , X. Chen , K. Sun , et al., “Direct Oxygen‐Oxygen Cleavage Through Optimizing Interatomic Distances in Dual Single‐Atom Electrocatalysts for Efficient Oxygen Reduction Reaction,” Angewandte Chemie (International ed in English) 62, no. 17 (2023): e202301833, 10.1002/anie.202301833. 36853880

[37] W. Zhou , H. Su , W. Cheng , et al., “Regulating the Scaling Relationship for High Catalytic Kinetics and Selectivity of the Oxygen Reduction Reaction,” Nature Communications 13, no. 1 (2022): 6414, 10.1038/s41467-022-34169-w. PMC9613657 36302910

[38] J. Liu , H. Xu , J. Zhu , and D. Cheng , “Understanding the Pathway Switch of the Oxygen Reduction Reaction From Single‐ to Double‐/Triple‐Atom Catalysts: A Dual Channel for Electron Acceptance–Backdonation,” Journal of the American Chemical Society Au 3, no. 11 (2023): 3031–3044, 10.1021/jacsau.3c00432. 38034973 PMC10685438

[39] L. Xu , X. Wang , X. Hu , et al., “Artificial‐Intelligence‐Assisted Design Principle For Developing High‐Performance Single‐Atom Catalysts,” Innovation (Camb) 6, no. 7 (2025): 100911. 40697790 10.1016/j.xinn.2025.100911 PMC12277759

[40] V. Viswanathan , H. A. Hansen , J. Rossmeisl , and J. K. Nørskov , “Universality in Oxygen Reduction Electrocatalysis on Metal Surfaces,” ACS Catalysis 2, no. 8 (2012): 1654–1660, 10.1021/cs300227s.

[41] T. He , S. K. Matta , G. Will , and A. Du , “Transition‐Metal Single Atoms Anchored on Graphdiyne as High‐Efficiency Electrocatalysts for Water Splitting and Oxygen Reduction,” Small Methods 3, no. 9 (2019): 1800419, 10.1002/smtd.201800419.

[42] I. C. Man , H. Y. Su , F. Calle‐Vallejo , et al., “Universality in Oxygen Evolution Electrocatalysis on Oxide Surfaces,” ChemCatChem 3, no. 7 (2011): 1159–1165, 10.1002/cctc.201000397.

[43] C. Liu , T. Li , X. Dai , et al., “Catalytic Activity Enhancement on Alcohol Dehydrogenation via Directing Reaction Pathways From Single‐ to Double‐Atom Catalysis,” Journal of the American Chemical Society 144, no. 11 (2022): 4913–4924, 10.1021/jacs.1c12705. 35261231

[44] J. Jiao , Q. Yuan , M. Tan , et al., “Constructing Asymmetric Double‐Atomic Sites For Synergistic Catalysis Of Electrochemical CO_2_ Reduction,” Nature Communications 14, no. 1 (2023): 6164, 10.1038/s41467-023-41863-w. PMC10547798 37789007

[45] X. Zhu , D. Zhang , C.‐J. Chen , et al., “Harnessing the Interplay of Fe–Ni Atom Pairs Embedded In Nitrogen‐Doped Carbon for Bifunctional Oxygen Electrocatalysis,” Nano Energy 71 (2020): 104597, 10.1016/j.nanoen.2020.104597.

[46] G. Yang , J. Zhu , P. Yuan , et al., “Regulating Fe‐Spin State by Atomically Dispersed Mn‐N in Fe‐N‐C Catalysts With High Oxygen Reduction Activity,” Nature Communications 12, no. 1 (2021): 1734, 10.1038/s41467-021-21919-5. PMC7979714 33741940

[47] Y. He , X. Yang , Y. Li , et al., “Atomically Dispersed Fe–Co Dual Metal Sites as Bifunctional Oxygen Electrocatalysts for Rechargeable and Flexible Zn–Air Batteries,” ACS Catalysis 12, no. 2 (2022): 1216–1227.

[48] Q. Sun , X. Yue , L. Yu , et al., “Well‐Defined Co_2_ Dual‐Atom Catalyst Breaks Scaling Relations of Oxygen Reduction Reaction,” Journal of the American Chemical Society 146, no. 51 (2024): 35295–35304. 39660442 10.1021/jacs.4c12705

[49] Y. Zhou , W. Yang , W. Utetiwabo , et al., “Revealing of Active Sites and Catalytic Mechanism in N‐Coordinated Fe, Ni Dual‐Doped Carbon With Superior Acidic Oxygen Reduction Than Single‐Atom Catalyst,” Journal of Physical Chemistry Letters 11, no. 4 (2020): 1404–1410, 10.1021/acs.jpclett.9b03771. 32004006

[50] Y. Chen , J. Mao , H. Zhou , et al., “Coordination Shell Dependent Activity of CuCo Diatomic Catalysts for Oxygen Reduction, Oxygen Evolution, and Hydrogen Evolution Reaction,” Advanced Functional Materials 34, no. 10 (2023): 2311664, 10.1002/adfm.202311664.

[51] Z. Lu , B. Wang , Y. Hu , et al., “An Isolated Zinc–Cobalt Atomic Pair for Highly Active and Durable Oxygen Reduction,” Angewandte Chemie (International ed in English) 58, no. 9 (2019): 2622–2626, 10.1002/anie.201810175. 30600864

[52] Y. Li , B. Wei , M. Zhu , et al., “Synergistic Effect of Atomically Dispersed Ni–Zn Pair Sites for Enhanced CO_2_ Electroreduction,” Advanced Materials 33, no. 41 (2021): e2102212, 10.1002/adma.202102212. 34463377

[53] H. Wang , Y. Shao , S. Zhou , C. Zhang , and N. Xiu , “Support Vector Machine Classifier via Soft‐Margin Loss,” IEEE Transactions on Pattern Analysis and Machine Intelligence 44, no. 10 (2022): 7253–7265, 10.1109/TPAMI.2021.3092177. 34166185

[54] A. B. Parsa , A. Movahedi , H. Taghipour , S. Derrible , and A. K. Mohammadian , “Toward Safer Highways, Application of XGBoost and SHAP for Real‐time Accident Detection and Feature Analysis,” Accident Analysis and Prevention 136 (2020): 105405, 10.1016/j.aap.2019.105405. 31864931

[55] R. O. Alabi , M. Elmusrati , I. Leivo , A. Almangush , and A. A. Makitie , “Machine Learning Explainability in Nasopharyngeal Cancer Survival Using LIME and SHAP,” Scientific Reports 13, no. 1 (2023): 8984, 10.1038/s41598-023-35795-0. 37268685 PMC10238539

[56] P. Guo , B. Liu , F. Tu , et al., “Breaking Sabatier's Vertex via Switching The Oxygen Adsorption Configuration And Reaction Pathway On Dual Active Sites For Acidic Oxygen Reduction,” Energy & Environmental Science 17, no. 9 (2024): 3077–3087, 10.1039/D4EE00823E.

[57] Z. Xiao , P. Sun , Z. Qiao , et al., “Atomically Dispersed Fe‐Cu Dual‐Site Catalysts Synergistically Boosting Oxygen Reduction for Hydrogen Fuel Cells,” Chemical Engineering Journal 446 (2022): 137112, 10.1016/j.cej.2022.137112.

[58] S. Sarkar , A. Biswas , T. Purkait , M. Das , N. Kamboj , and R. S. Dey , “Unravelling the Role of Fe–Mn Binary Active Sites Electrocatalyst for Efficient Oxygen Reduction Reaction and Rechargeable Zn‐Air Batteries,” Inorganic Chemistry 59, no. 7 (2020): 5194–5205, 10.1021/acs.inorgchem.0c00446. 32191443

[59] M. Xiao , Y. Chen , J. Zhu , et al., “Climbing the Apex of the ORR Volcano Plot via Binuclear Site Construction: Electronic and Geometric Engineering,” Journal of the American Chemical Society 141, no. 44 (2019): 17763–17770, 10.1021/jacs.9b08362. 31603677

[60] Z. Chen , X. Su , J. Ding , et al., “Boosting Oxygen Reduction Reaction With Fe and Se Dual‐atom Sites Supported by Nitrogen‐doped Porous Carbon,” Applied Catalysis B‐Environmental 308 (2022): 121206, 10.1016/j.apcatb.2022.121206.

[61] V. Tripković , E. Skúlason , S. Siahrostami , J. K. Nørskov , and J. Rossmeisl , “The Oxygen Reduction Reaction Mechanism on Pt(111) From Density Functional Theory Calculations,” Electrochimica Acta 55, no. 27 (2010): 7975–7981, 10.1016/j.electacta.2010.02.056.

[62] M. Xiao , J. Zhu , S. Li , et al., “3d‐Orbital Occupancy Regulated Ir‐Co Atomic Pair toward Superior Bifunctional Oxygen Electrocatalysis,” ACS Catalysis 11, no. 14 (2021): 8837–8846, 10.1021/acscatal.1c02165.

[63] N. Zhang , T. Zhou , J. Ge , et al., “High‐Density Planar‐Like Fe_2_N_6_ Structure Catalyzes Efficient Oxygen Reduction,” Matter 3, no. 2 (2020): 509–521, 10.1016/j.matt.2020.06.026.

[64] M. Liu , H. Chun , T.‐C. Yang , et al., “Tuning the Site‐to‐Site Interaction in Ru–M (M = Co, Fe, Ni) Diatomic Electrocatalysts to Climb up the Volcano Plot of Oxygen Electroreduction,” ACS Nano 16, no. 7 (2022): 10657–10666. 35834391 10.1021/acsnano.2c02324

[65] Z. Yu , C. Si , A. P. LaGrow , et al., “Iridium–Iron Diatomic Active Sites for Efficient Bifunctional Oxygen Electrocatalysis,” ACS Catalysis 12, no. 15 (2022): 9397–9409, 10.1021/acscatal.2c01861.

[66] C. Zhang , Y. Guo , C. Chen , et al., “Accelerated O Horizontal Line O Bond Cleavage and Stabilized Fe Sites by Synergistic D Horizontal Line p Fe Horizontal Line Sn Dual‐Atom Pair for Enhanced Oxygen Reduction,” Angewandte Chemie (International ed in English) 138, no.7 (2026): e24265, 10.1002/ange.202524265. 41486582

[67] X. Wang , N. Zhang , S. Guo , et al., “p‐d Orbital Hybridization Induced by Asymmetrical FeSn Dual Atom Sites Promotes the Oxygen Reduction Reaction,” Journal of the American Chemical Society 146, no. 31 (2024): 21357–21366, 10.1021/jacs.4c03576. 39051140

[68] B. Ni , R. Chen , L. Wu , P. Sun , and T. Chen , “Encapsulated FeP Nanoparticles With in‐situ Formed P‐doped Graphene Layers: Boosting Activity in Oxygen Reduction Reaction,” Science China Materials 64, no. 5 (2020): 1159–1172, 10.1007/s40843-020-1525-7.

