# Supplementary material for: Dual‐Sabatier Optima: How Reaction Mechanism Determines Activity Volcano Map of Dual‐Atom Catalysts for Oxygen Reduction Reaction
Source: Angew Chem Int Ed Engl. 2026 Apr 27;65(24):e8386838. doi: 10.1002/anie.8386838 (PMC13245613; doi:10.1002/anie.8386838)
Supplement: Supplementary file 1 — The detailed calculation methods of thermodynamic stability analysis and pre‐adsorption phase diagrams used in this work; the structures and adsorption energies of reactants, intermediates, transition states and products; the microscopic kinetic models and the comparison of predicted results with experimental results regarding polarization curves; as well as the analysis related to the O2* adsorption process, descriptor mining, and the reaction pathways of ORR.Supporting File 1: anie72390‐sup‐0001‐SuppMat.docx. [file ANIE-65-e8386838-s001.docx]

**SUPPORTING INFORMATION**

**Dual-*Sabatier* Optima：How Reaction Mechanism Determines Activity Volcano Map of Dual-Atom Catalysts for Oxygen Reduction Reaction**

Jin Liu^1,2^, Hao Li^3*^, Haoxiang Xu^1,2,4*^ and Daojian Cheng^1,2,4*^

^1^Beijing Key Laboratory of Intelligent Design and Manufacturing for Hydrogen Energy Materials, and College of Chemical Engineering, Beijing University of Chemical Technology, Beijing 100029, China

^2^State Key Laboratory of Organic-Inorganic Composites, Beijing University of Chemical Technology, Beijing 100029, China

^3^Advanced Institute for Materials Research (WPI-AIMR), Tohoku University, Sendai 980-8577, Japan.

^4^Deep Intelligence Experiment Technology (Beijing) Co., Ltd., Beijing 100029, China

^*^E-mail: c[hengdj@mail.buct.edu.cn](mailto:chengdj@mail.buct.edu.cn); [xuhx@mail.buct.edu.cn](mailto:xuhx@mail.buct.edu.cn); li.hao.b8@tohoku.ac.jp

**Section 1 Supplementary Method**

**S1.1 First-principles computations**

All the computations were carried out by spin-polarized DFT method, as implemented in Vienna *ab initio* Simulation Package (VASP) code^1^. The exchange-correlation energy was modeled using the Perde-Burke-Ernzerhof (PBE) functional^2, 3^. Among the extensive theoretical computational works, the N-doped graphene monolayer has been regarded as an efficient theoretical model to represent the carbon-based support of DACs^4-6^. In this work, the graphene was modeled with a (5x5) supercell, and a vacuum slab of 25 Å was inserted in the *z* direction for surface isolation to prevent interaction between two neighboring surfaces. An energy cutoff of 500 eV was adopted for the plane-wave basis. In structural optimizations, the Brillouin zone was sampled by 3×3×1 k-points using the Monkhorst−Pack scheme, while denser k-points of 6×6×1 were employed for electronic property computations. The energy convergence criterion is 10^-5^ eV, and the force convergence criterion is 0.02 eV A^-1^. DFT-D3 (D stands for dispersion) with Grimme’s empirical correction was employed to describe van der Waals (vdW) interactions. It is generally recognized that the localized 3d electrons correlation for transition metal in fourth period can be described by considering on-site coulomb (U) and exchange (J) interactions, a key element in the DFT + U method. Here we applied DFT + U through the rotationally invariant approach^7, 8^ with the corresponding U - J values for different transitions listed in Supplementary Table S15. The adsorption free energy of each adsorbate is defined as:

Δ*G* = Δ*E* – TΔ*S* + Δ*E_ZPE_ +* Δ*E*_solv_ (1)

where the Δ*E* is the energy change from DFT calculations considering the solvent effect, Δ*E_ZPE_* is the zero-point energy change, and Δ*S* is the entropy change at 298.15 K. The adsorption free energy Δ*G*_OOH*_, Δ*G*_O+OH*_, Δ*G*_O*_, Δ*G*_2OH*_, and Δ*G*_OH*_ are relative to the free energy of stoichiometrically appropriate amounts of H_2_O (l) and H_2_ (g). The solvent effect was tested using an implicit solvation model in the VASPsol code^9, 10^. At the common ORR reaction temperature of 298.15 K, the dielectric constant of water is 78.36 F/m. Referring to relevant calculation references^11^, the dielectric constant of 80 was approximately set to simulate the aqueous electrolyte. The eﬀective surface tension parameter was assigned to 0 in VASPsol to neglect the cavitation energy contribution. The Debye length is set to 3.0 Å. Δ*E*_solv_ refers to the stabilization of the adsorbate because of the interaction with the surrounding water. The average Δ*E*_solv_ values of each system adsorbed with O* + OH*, OOH*, 2OH*, O*, and OH* calculated are shown in Supplementary Table S1.

Activation barriers for all proton-electron transfer steps of the intermediates were set to 0.26 eV, taken from Pt (111)^12^, which has been successfully applied in DACs^13^. We calculated the activation barriers of the reaction involved with the O−O bond breaking, using the CINEB method^14^. In addition, we also adopted AIMD and the "slow-growth" sampling method to simulate the O−O bond breaking process.

**S1.2 Slow-growth method**

We employed constrained AIMD and a “slow-growth” sampling approach to simulate the O-O bond breaking process. The Nose-Hoover thermostat was adopted to keep the temperature at 300 K, and the time step was set to 1 fs. The annealing temperature velocities were rescaled every 20 steps to readjust the target temperature. Each state of the O-O bond breaking process on different DACs was obtained through AIMD simulation, and the collective variable (CV) increment was set to 0.0005 Å, depending on the length of the O-O bond (0-4 Å). All the AIMD simulations were performed by using the gamma point of the Brillouin zone. The convergence criterion for the electronic step is set to 1×10^−4^, and the POMASS of H is set to 2.

**S1.3 Constant-potential computations**

Different amounts of charges were introduced into the cell to simulate an electrode potential system of 0.9 V *vs.* RHE. The solvation environment was included by applying the implicit solvation model VASPsol, which solves the linearized Poisson-Boltzmann equation. The dielectric constant of water was set to a relative permittivity of 78.4, and the surface tension was set to zero. Inside, the work function (W_f_) of a system is calculated as the difference between the vacuum level (E_vac_) and the Fermi level (E_F_), and E_vac_ is extracted from the planar-averaged electrostatic potential profile, with 10^−5^ e/Å^3^ being chosen as the cutoff value of the electron density^15^. Then, the electrode potential versus the reversible hydrogen electrode (U_RHE_) can be calculated, which is defined as:

U_RHE_ = (W_f_ - 4.43)/*e* + 0.0592 × pH (2)

where 4.43 is the work function of the standard hydrogen electrode^16^; *e* is the electron charge; pHs are set to 1 for the acid system and 13 for the alkaline system, according to the used experimental electrolytes (0.1M HClO4 and 0.1M KOH)^17^. To convert constant-charge free energy differences to constant-potential ones, quadratic functions are applied to fit the free energy Δ*G*(U) of each species against the potential. The specific fitting formula is shown in Supplementary **Tables S4-S5**. Finally, the quadratic relations between the reaction free energy Δ*G*(U) and the electric potential are obtained by subtracting the free energy curves of the involved species.

It is worth noting that while the constant-potential correction based on VASPsol and quadratic fitting captures the essential energetic response to the electrode potential, this implicit solvation approach has certain theoretical limitations. Specifically, it may not fully account for the complex Fermi level shifts and non-linear electronic shielding within the electrochemical double layer (EDL) as rigorously as advanced continuous-charge models (e.g., JDFTx with the CANDEL model). However, we justify the reliability of our results for the following reasons: (1) Our study focuses on the comparative activity trends among different dual-metal sites. Since the systematic errors in Fermi level alignment tend to cancel out when calculating the relative binding energy differences of similar intermediates on related surfaces, the predicted scaling relations remain robust. (2) To mitigate the limitations of the implicit model, we have incorporated explicit water molecules to describe local hydrogen-bonding effects. (3) The high consistency between our simulated polarization curves (e.g., onset and half-wave potentials) and experimental data further validates that this computational framework provides a physically sound and reliable description of the catalytic trends for these dual-atom systems.

**S1.4 The explicit solvation effect of ORR adsorbates**

The solvation effects were modeled via AIMD simulations with explicit water molecules. Specifically, the AIMD simulations were performed using VASP with the PBE functional and the D3 dispersion correction scheme, which is known to accurately describe metal-water interfaces. Our simulations employed a plane-wave cutoff energy of 400 eV and a Gaussian smearing width of 0.1 eV. The electronic structure was relaxed until all forces converged to below 0.05 eV/Å. Γ-point AIMD simulations were carried out with a time step of 1 fs, using a Nosé thermostat set at 300 K. The metal-water interface in the supercell contained 20 water molecules, corresponding to at least three layers of static water. For each surface-adsorbate combination, the molecular-dynamics trajectory was run for 3 ps to account for hydrogen-bonding orientations. Data sampling commenced after a 1 ps equilibration period, yielding at least eight distinct surface-adsorbate configurations. These configurations were then allowed to relax until a force convergence threshold of 0.05 eV Å^-1^ was reached, and the solvation effects were calculated using Equations (3)-(5):

$E_{{OH}^{*}}^{solv}=E_{slab-{OH}^{*}}^{solv}-E_{slab}^{solv}-E_{H_{2}O}+\frac{1}{2}E_{H_{2}}$ (3)

$E_{O{OH}^{*}}^{solv}=E_{slab-O{OH}^{*}}^{solv}-E_{slab}^{solv}-2E_{H_{2}O}+\frac{3}{2}E_{H_{2}}$ (4)

$E_{\mathrm{ads}^{*}}^{\mathrm{correction}}=E_{\mathrm{ads}^{*}}^{solv}-E_{\mathrm{ads}^{*}}^{vaccum}$ (5)

where $E_{slab-\mathrm{ads}^{*}}^{solv}$represents the total energy of the surface with adsorbate and water molecules. $E_{slab}^{solv}$ denotes the total energy of a slab surface with water molecules. All of these energies were obtained from the structural relaxations. Using these calculations, we present the mean values of the adjusted adsorption energies of OOH* and O*+OH* in **Table S16 and Fig. S20**. The calculation results show that intermediate solvation energies vary consistently between the explicit AIMD model and the implicit solvation model (VASPsol) used in our work. This consistent trend confirms that the implicit model is used to treat long-range electrostatic and overall solvation effects. Specifically, explicit hydrogen bonding fine-tunes the absolute adsorption energies of polar intermediates, but these adjustments do not change the relative energy ordering of different intermediates. Thus, the key findings are: (1) the implicit model's reliability is supported, and (2) explicit hydrogen bonding affects absolute but not relative adsorption energies.

**S1.5 Machine learning method**

In this work, the ML methods were implemented using the open-source Scikit-learn package in the Python 3 environment. We constructed two ML models, RF Classification and K-Nearest Neighbor (KNN) Classification. Although these models have relatively simple structures, they can provide interpretable insights into the interactions between variables. And each ML model was hyperparameter-tuned and quintuple-cross-validated to ensure optimal performance and reliability. The performance of the ML model was evaluated by the probability of correct predictions (Accuracy). The test results showed that RF's accuracy was 0.85, while KNN's was 0.71. This indicates that the predictive performance of the RF model is stronger, and the prediction models used in this work for the diatomic oxygen adsorption model are all based on RF calculations.

In SISSO^18^, the feature construction was performed by creating all the possible mathematical combinations, within certain feature complexity, between the primary features using the operators { +, −, ·, /, log, exp, exp−, −1, 2, 3, √, ∛, |−| }. The code for VS-SISSO and the user guide are available at <https://github.com/rouyang2017/SISSO.> The RMSE and the coefficient of determination (R^2^) were used to evaluate the performance of the SISSO model; the higher the R^2^ and the lower the RMSE, the better the model.

**S1.6 Polarization curve simulation**

Following the kinetic model developed by Hansen et al.^19^, we simulated the polarization curve of DACs. Here, we highlight the construction of a complete ORR pathway on DACs, and electrochemical O_2_* protonation producing O*+OH* is identified to be quite thermodynamically and kinetically favorable. The O_2_ molecule diffusion adsorption, and electrochemical reduction steps are listed by the following equations:

$$R1: O_{2(aq,electrolyte)}\frac{k1}{k-1} O_{2(aq,interface)}$$

$$R2: O_{2(aq,interface)}+ *\frac{k2}{k-2} O_{2}^{*}$$

$$R3: O_{2}^{*}+H^{+}+ e^{-} \frac{k3}{k-3} \mathrm{OOH}^{*}$$

$$R4: O_{2}^{*}+H^{+}+ e^{-} \frac{k4}{k-4} [{O^{*}+OH}^{*}]$$

$$R5: \mathrm{OOH}^{*}+H^{+}+ e^{-} \frac{k5}{k-5} O^{*} + H_{2}O$$

$$R6: [{O^{*}+OH}^{*}]+H^{+}+ e^{-} \frac{k6}{k-6} [ {2OH}^{*} ]$$

$$R7: O^{*}+H^{+}+ e^{-} \frac{k7}{k-7} \mathrm{OH}^{*}$$

$$R8: [{2OH}^{*}]+H^{+}+ e^{-} \frac{k8}{k-8} \mathrm{OH}^{*} + H_{2}O$$

$$R9: \mathrm{OH}^{*}+H^{+}+ e^{-} \frac{k9}{k-9} H_{2}O + *$$

O_2(aq,electrolyte)_ and O_2(aq,interface)_ represent O_2_ in the electrolyte and the catalyst-electrolyte in interface, respectively. Note that equations R_1_ and R_2_ are non-electrochemical steps, and the rest of equations are electrochemical steps. *θ* represents the coverage of the species. Based on the above reduction steps, we can gained the rate equations of each species, such as,

$\frac{\partial\chi_{O2(dl)}}{\partial t}$ = $k_{1}\chi_{O2(aq)} - k_{-1}\chi_{O2(dl)} - k_{2}\chi_{O2(dl)}\theta^{*} +k_{-2} \theta_{O_{2}^{*}}$

$\frac{\partial\theta_{O_{2}^{*}}}{\partial t}$ = $k_{2}\chi_{O2(dl)}\theta^{*} - k_{-2} \theta_{O_{2}^{*}} - k_{3}\theta_{O_{2}^{*}} + k_{-3}\theta_{OOH}^{*}$ - $k_{7}\theta_{O_{2}^{*}} - k_{-7}\theta_{[O^{*}+{OH}^{*}]}$

To account for the mass transfer limitation, the transport of O_2_ from the electrolyte to the interface is governed by Fick’s first law. The net rate of O_2_ diffusion (R_diff_) is expressed as:

$R_{\mathrm{diff}}=k_{\mathrm{diff}} (\chi_{O2(aq,electrolyte)}- \chi_{O2(aq,interface)}$)

where k_diff_ is the diffusion rate constant related to the Nernstian diffusion layer thickness. In these equations χ_H2O_ = 1 and χ_O2(aq)_ = 2.34*10^-5^ corresponding to 1 atm O_2(g)_ in equilibrium with O_2(aq)_. Additionally, site conservation on the DACs sites must be satisfied

$1= \theta_{O2*} + \theta_{OOH*} + \theta_{O*} + \theta_{OH*} + \theta_{*} + \theta_{[O*+OH*]} + \theta_{[2OH*]}$

The rate equations are solved numerically at steady state, and further infer the turn over frequency (TOF).

For non-electronchemical step i,its equilibrium constant (K) can be expressed as:

K_i_ = exp ($-\frac{\triangle Gi}{k_{B}T} )$

where △G_i_ is the free energy change of step i, and k_B_ is the Boltzmann constant. The rate constant (k_i_) is given by:

k_i_ = $\frac{k_{B}T}{h}$ exp (-$\frac{E_{a,i}}{k_{B}T} )$

where E_a,i_ is the activation energy.

While for electrochemical step, K_i_ is associated with the reaction potential (U vs RHE), given by:

K_i_ = exp ($-\frac{e(U-Ui)}{k_{B}T} )$

where U_i_ is the reversible potential of step i deduced by U_i_=-G_i_/e.

And the k_i_ for electrochemical step, associated with the reaction potential (U vs RHE), is written as:

k_i_ = $\frac{k_{B}T}{h}$ exp ($-\frac{E_{a,i}}{k_{B}T} )$ exp ($-\frac{e\beta i(U-Ui)}{k_{B}T} )$

where β_i_ is the symmetric factor taken as 0.5^20^. Since the E_a,i_ of electrochemical ORR steps are generally small range from 0.10 to 0.26 eV^12, 20, 21^, we adopted E_a,i_ =0.26 eV for all the electrochemical steps of ORR on DACs.

Moreover, the rate constants for all the reverse reaction (k_i_),can be deduced by:

$$k_{-i} = \frac{k_{i}}{K_{i}}$$

Finally, the current density (j) can be calculated by:

*j* = eρTOF_e-_

Importantly, at low potentials (e.g., < 0.8 V vs. RHE), the electrochemical consumption rate on the dual-metal sites far exceeds the diffusion supply. This causes χ_O2(aq,interface)_ to approach zero, leading to a potential-independent current density plateau:

$$j_{\mathrm{limit}}=ne\rho\text{∙}k_{\mathrm{diff}} \chi_{O2(aq,electrolyte)}$$

This rigorously explains the parallel line behavior in the simulated polarization curves, representing the transition to a pure mass-transfer-controlled regime.

**S1.7 Degree of Rate Control**

The degree of rate control (DRC) developed by Campbell was used to quantify the impact of the free energy perturbation δG_i_ of a specific species i on the total reaction rate, which has been widely adopted to identify the rate-controlling transition states (TS) and intermediates. We slightly modify the original DRC equation to express the impact of the reaction energy changes on the total reaction rate. The DRC_r_ is defined as

$${DRC}_{r,n}={(\frac{\partial lnr}{\partial\left( -\frac{G_{n}}{k_{B}T} \right)})}_{G_{m\neq n,}G_{i}^{TS}}$$

where G_n_ is the reaction energy and G_i_^TS^ is the free energy of a transition state. It describes the sensitivity of the total reaction rate to the reaction energy.

**Section 2 Supplementary Note**

**S2.1 The “electron acceptation-inversion dual-channel” mechanism**

According to our previous works on DACs^22^, the dissociation pathway of O-O bond breakage during the ORR process is feasible, and the activation of the O-O bond can be induced through the "electron acceptation-inversion dual-channel" mechanism. Taking FeRu-DAC as an example, we clarify the activation mechanism of O_2_* (Supplementary Fig. S1), based on projected density of state and partial differential charge density projecting to specific energy level^23, 24^. When adjacent bimetallic sites form bonds with both ends of O_2_*, the electrons of σ orbital of O-O bond contributed by O 2p_y_ (2p_z_) orbital transfer to d_xz_ and d_yz_ orbitals of Fe (Ru) in the energy level from -5 to -7 eV. At the same time, the electrons from partially filled Fe (Ru) d_xz_ orbital flow back into π* orbitals of O-O bond in the energy level of 0-2 eV. The O-O bond length in O_2_@FeRu is elongated to 1.42 Å, which can be attributed to strong hybridization between the Fe 3d_xz_ (3d_yz_), Ru 3d_xz_, and O 2p_y_ (2p_z_) orbitals. Such hybridization enhances delocalized electron transfer between the O_2_ molecule and the FeRu active site, thereby facilitating O_2_ dissociation upon protonation and initiating the dissociation pathway during ORR.

**S2.2 Physical meaning of individual descriptors and their combined effects**

(1) Physical meaning of individual descriptors

- **E_M1_/E_M2_** (oxidation enthalpy of the metal atoms M_1_/M_2_): This term reflects the tendency of the metal atoms to lose electrons, which directly influences charge transfer to adsorbed species. A lower (more negative) oxidation enthalpy generally indicates a stronger electron-donating ability, promoting the back-donation of electrons into the π* orbitals of O_2_, thereby facilitating side-on adsorption and O-O bond activation.
- **Bond_M1M2_** (optimized M_1_M_2_ bond length): This geometric parameter determines whether two adjacent metal sites can spatially accommodate the side-on adsorption of O_2_ and promote O-O bond cleavage. A suitable bond length (typically between 2.3-3.6 Å) enables the formation of a bridging O-O configuration (M_1_-O-O-M_2_), which is a prerequisite for the dissociative pathway.
- **N_num_** (number of bonds between the dual-metal site and N atoms): This parameter controls the local coordination environment and the electronic coupling with the carbon support. A higher N_num_ usually implies a more rigid and stable dual-atom structure, which can modulate the d-band center and the adsorption strength of intermediates.
- **Ӽ_M1_/Ӽ_M2_** (electronegativity of the M_1_/M_2_): Electronegativity regulates the electron distribution and bond polarity between the two metal atoms. A larger difference in electronegativity can induce an internal electric field, promoting asymmetric charge distribution and favoring the side-on adsorption of O_2_.
- **R_M1_/R_M2_** (atomic radii of the M_1_/M_2_): Atomic radii influence the spatial constraints and bonding geometry. Larger radii may lead to longer metal - metal distances, which could hinder the formation of a compact O_2_-bridged structure, while smaller radii may facilitate closer metal - metal proximity.

(2) Collective role in governing O_2_ adsorption configuration

① Electronic effects:

E_M1_/E_M2_ and Ӽ_M1_/Ӽ_M2_ jointly regulate the d-band center and charge-transfer capability of the dual-atom site. A suitable d-band center enables optimal interaction with O_2_ orbitals, promoting side-on adsorption and weakening the O-O bond.

② Geometric effects:

Bond_M1M2_ and R_M1_/R_M2_ determine whether the dual-site can geometrically accommodate the side-on adsorption of O_2_. The Bond_M1M2_ term is particularly critical, as it directly appears in the SISSO expression for O_2_ adsorption classification (Table 1).

③ Coordination effects:

N_num_ influences the rigidity and stability of the dual-site structure, which in turn affects the adsorption energy of intermediates such as OH*. A well-defined coordination environment ensures consistent electronic modulation across different DACs.

In summary, the SISSO-derived descriptors integrate key atomic and structural features that collectively govern the O_2_ adsorption configuration and the OH* binding energy. The E_M1_/E_M2_ and Ӽ_M1_/Ӽ_M2_ reflect the electronic properties of the metal centers, controlling charge transfer and d-band modulation. The Bond_M1M2_ and R_M1_/R_M2_ determine the geometric feasibility for side-on O_2_ adsorption. The N_num_ captures the local bonding environment and structural rigidity. Together, these features enable the prediction of whether a given DAC favors the dissociative pathway, which requires side-on O_2_ adsorption and optimized OH* binding for high ORR activity.

**S2.3 Thermodynamic and electrochemical stability analysis**

The thermodynamic and electrochemical stabilities of these models were evaluated by the anti-aggregation energy (E_anti-agg_) and dissolution potential (U_diss_), which are defined as:

E_anti-agg_=E_M1M2-NC_–E__M1-NC_/E__M2-NC_–E_M,bulk_

U_diss_=U^0^_diss(metal,bulk)_-E_anti-agg_/***n***e

where E_M,bulk_ is the total energy of the metal atom in its most stable bulk structure, E__M1-NC_ and E__M2-NC_ are the total energies of M_1_M_2_-NC with a M_1_/M_2_ atom being dissolved, and U^0^_diss_ and ***n*** being the standard dissolution potential of the corresponding bulk metal and the number of transferred electrons involved in the dissolution (Supplementary Table S9). According to our definition, if E_anti-agg_ < 0 eV, the configuration is thermodynamically stable. As for systems containing both M_1_ and M_2_ atoms, if E_anti-agg_ = max[E_anti-agg-M1_, E_anti-agg-M2_] < 0 eV, then this configuration is considered thermodynamically stable. And if U_diss_ > 0 V *vs* RHE, then it is considered electrochemically stable. Supplementary Fig. S4 and Table S6 show the E_anti-agg_ and U_diss_ values for the DACs, and 42 systems that meet the stability criteria were finally screened out.

**S2.4 Pre-adsorption phase diagram**

In this study, we present and discuss the surface preadsorption phase diagram, which allows us to analyze the most stable pre-adsorption situation of each system under the operating potential:

*G*= *G*_[U=0,pH=0]_-*m*eU

A spontaneously absorbed ORR intermediate (OH*, O*, or O_2_*) with the lowest free energy (*G*) at a common ORR working potential (U=0.9 V *vs*. RHE) and a wide pH range determines the stable form of DACs in those conditions. Each intermediate species along the catalytic path for ORR is characterized by a straight line with a negative slope, which is determined by the number of electrons to be transferred. The *m* is number of electrons to be transferred (1 for *m*-*G*(OH*), 2 for *m*-*G*(O*), and 4 for *m*-*G*(clean slab)). For example, FeCo-1 needs to pre-adsorb an OH*, and the clean surface of CoIr-2 is regarded as the most stable under ORR working potential (Supplementary Fig. S5a-d). Therefore, the most stable form of 42 stable systems (Supplementary Figs. S6-S7) is determined for further study of the ORR reaction free energy.

**S2.5 Discussion of Minor Differences between Theory and Experiment**

The minor discrepancies (e.g., a 0.03 V difference in E_1/2_ for the FeRu system) can be attributed to: (i) our DFT model assumes ideal, defect-free active site structures, whereas real catalysts contain inhomogeneities; (ii) differences in local coordination environments (e.g., N-doping patterns, carbon defects); and (iii) our kinetic current curves do not include mass-transport contributions, while experimental curves are measured under rotating disk electrode conditions (mixed kinetic-diffusion control). Despite these minor differences, our theoretical method reproduces the overall trends and relative activity rankings well.

**Section 3 Supplementary Figures**


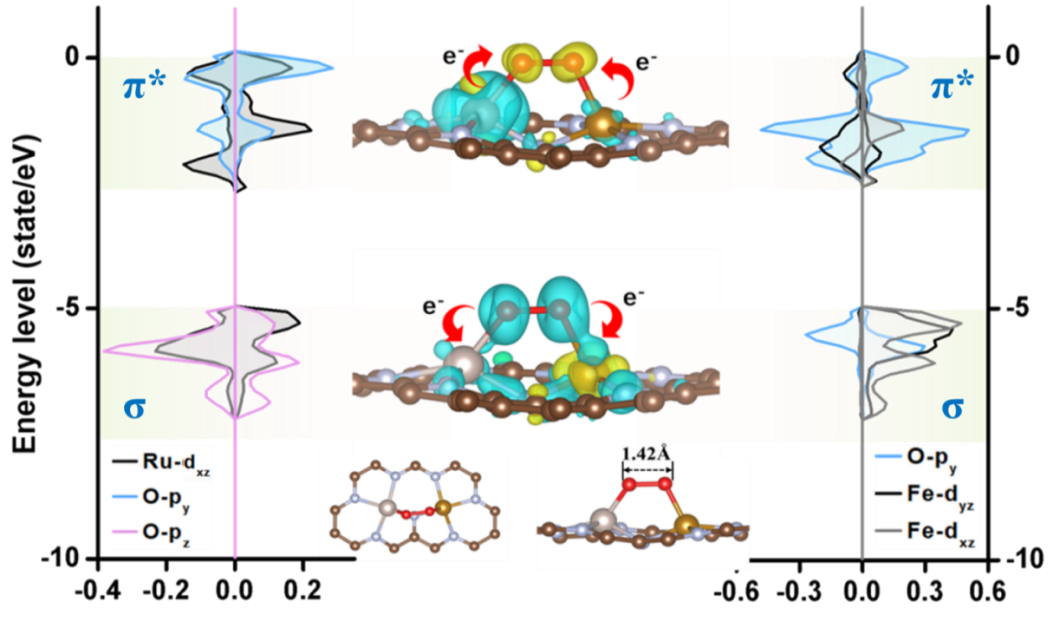


**Figure S1.** Partial differential charge density projecting to specific energy level, projected density of state between O_2_ molecule on FeRu-2 DAC (The blue area represents the loss of electrons, and the yellow area represents the gain of electrons. The Fermi energy is set to 0 eV )


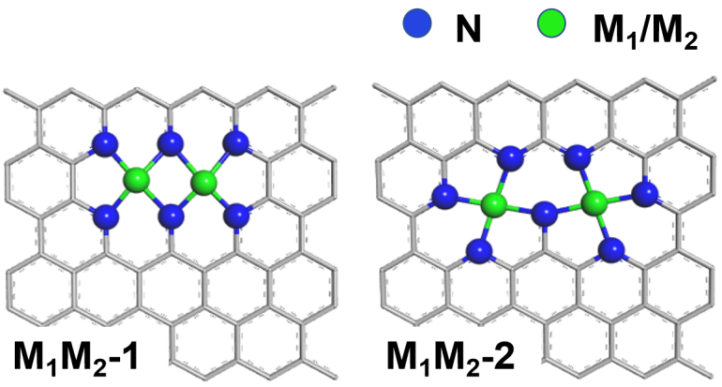


**Figure S2.** Two coordination configurations of dual-atom catalysts


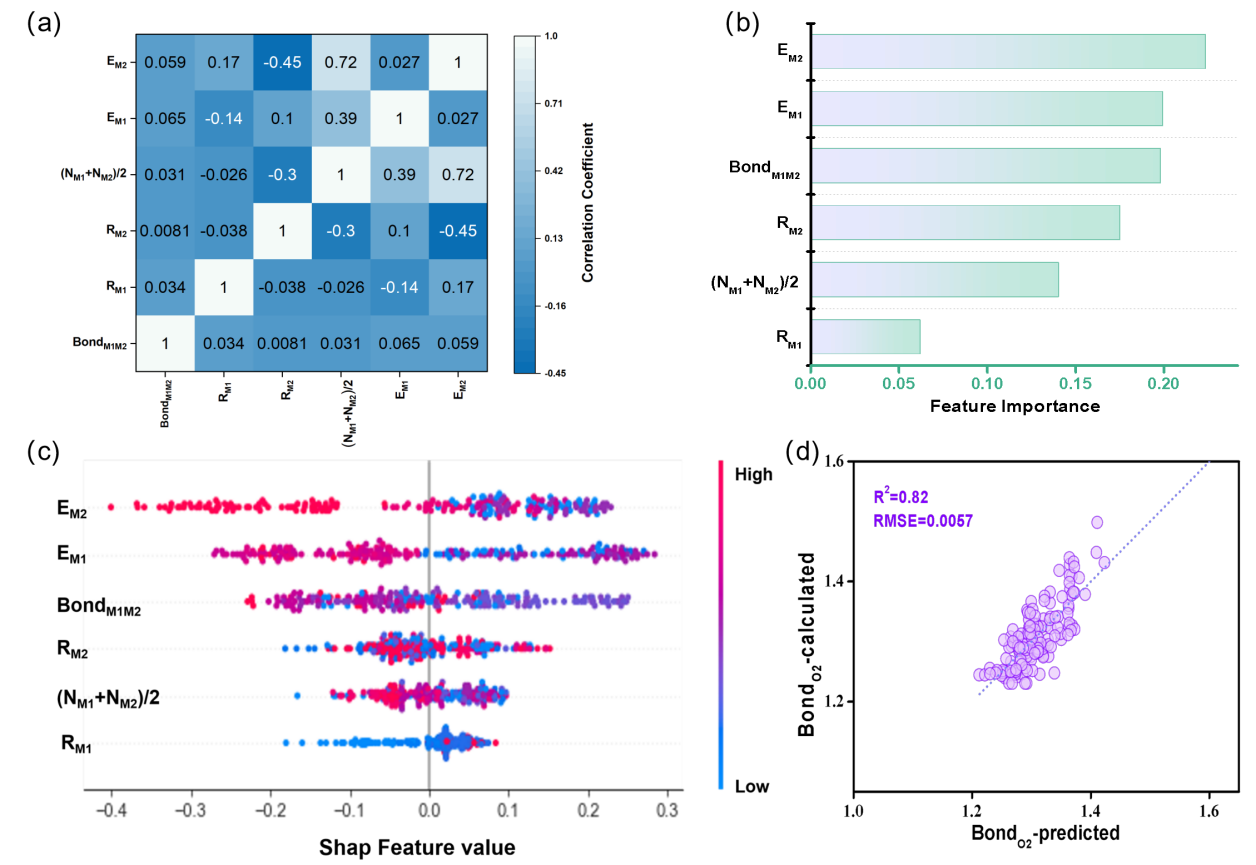


**Figure S3.** (a) A heatmap of the Pearson correlation coefficients between the nine features and their corresponding feature importances. SHAP diagram of RF model: (b) Analysis of the importance of the selected feature and (c) SHAP honeycomb diagram of the RF model. (d) The data distribution R^2^ and RMSE of Bond_O2_-predicted and Bond_O2_-calculated for machine learning models.


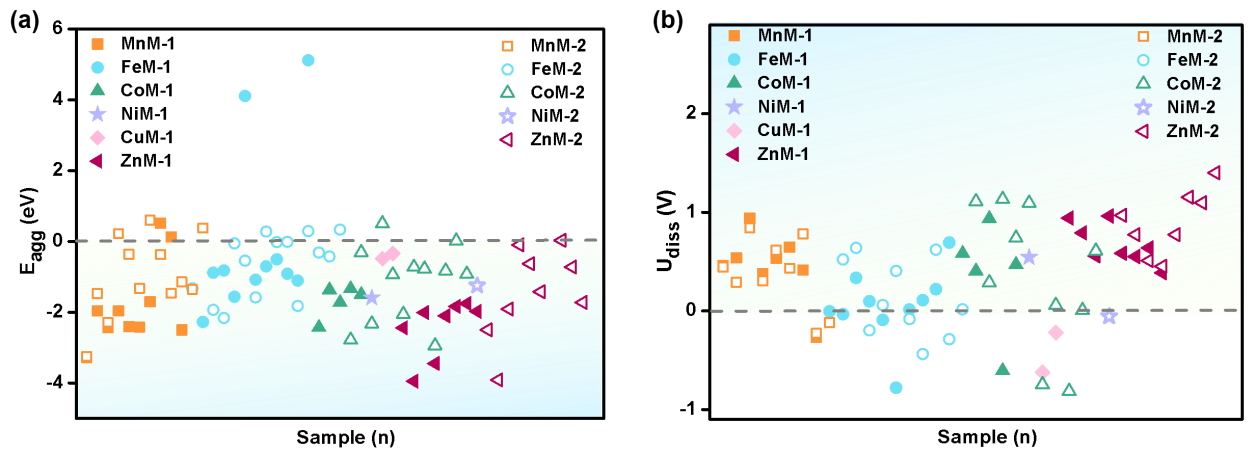


**Figure S4.** Computed (a) anti-aggregation energy and (b) dissolution potential of metal atoms in DACs.


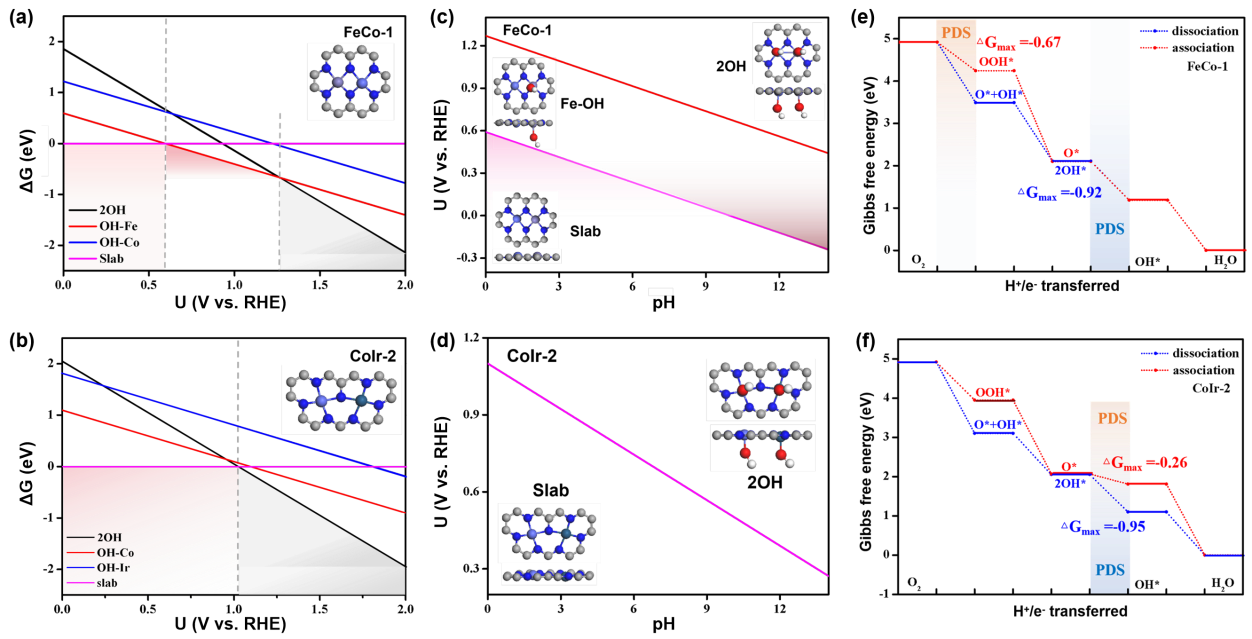


**Figure S5.** (a-d) The pre-adsorption phase diagrams of FeCo-1 and CoIr-2, and (e-f) the Gibbs free energy step diagrams of ORR under the dissociation mechanism and the corresponding association mechanism.


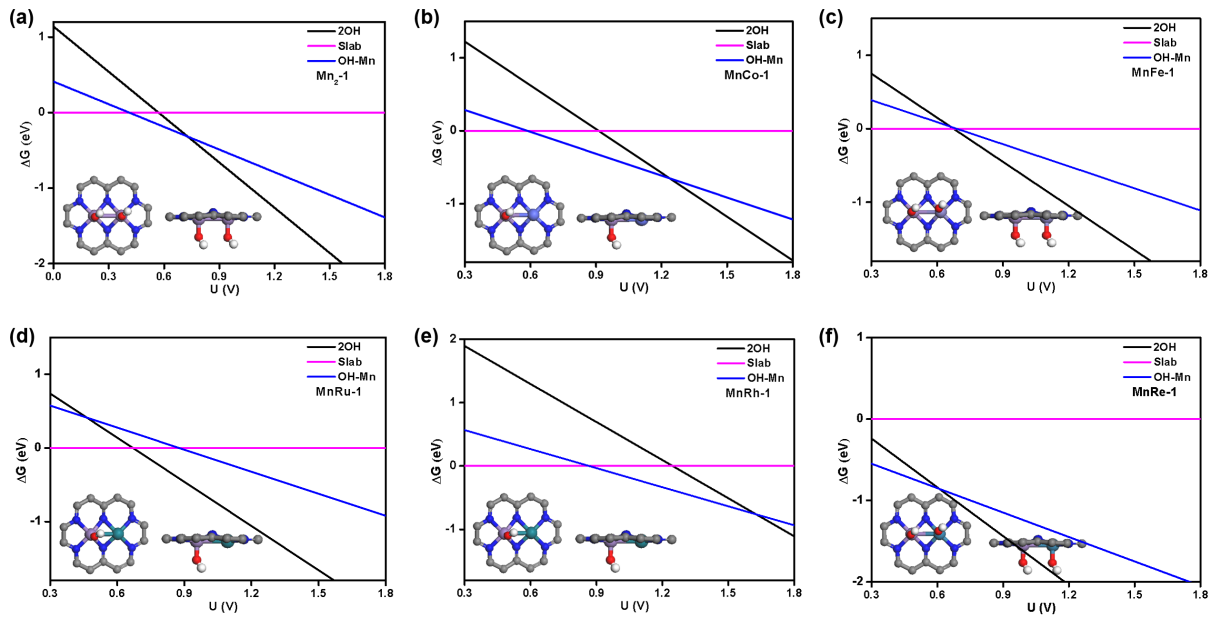


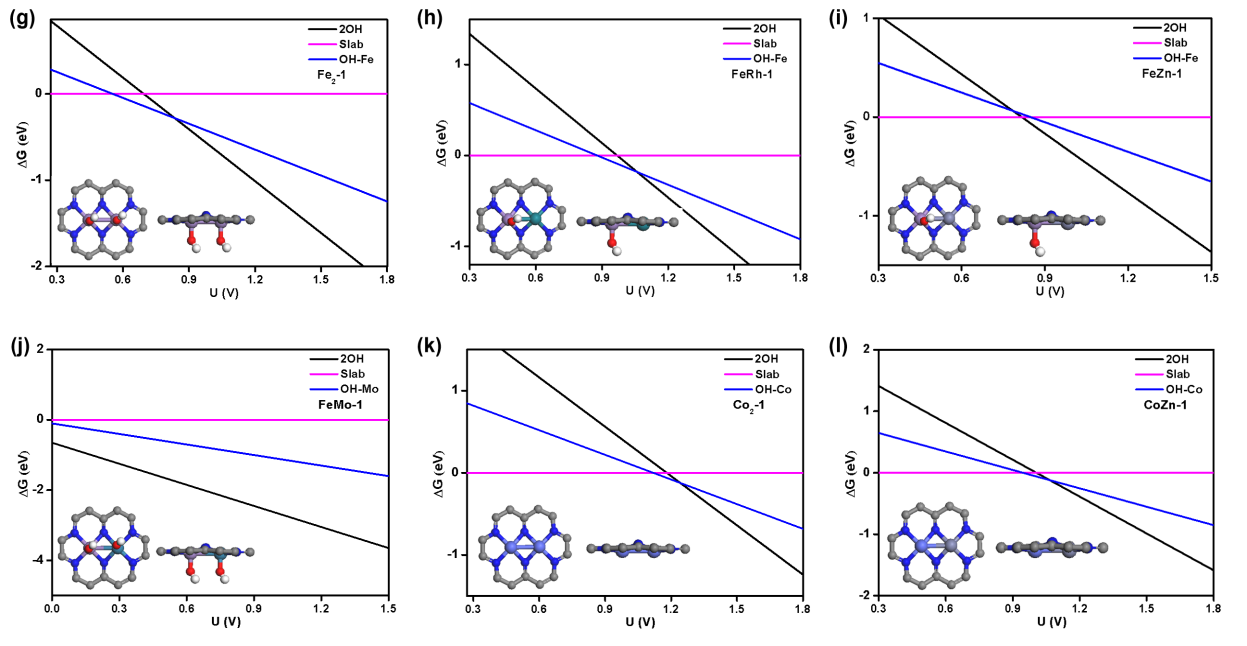


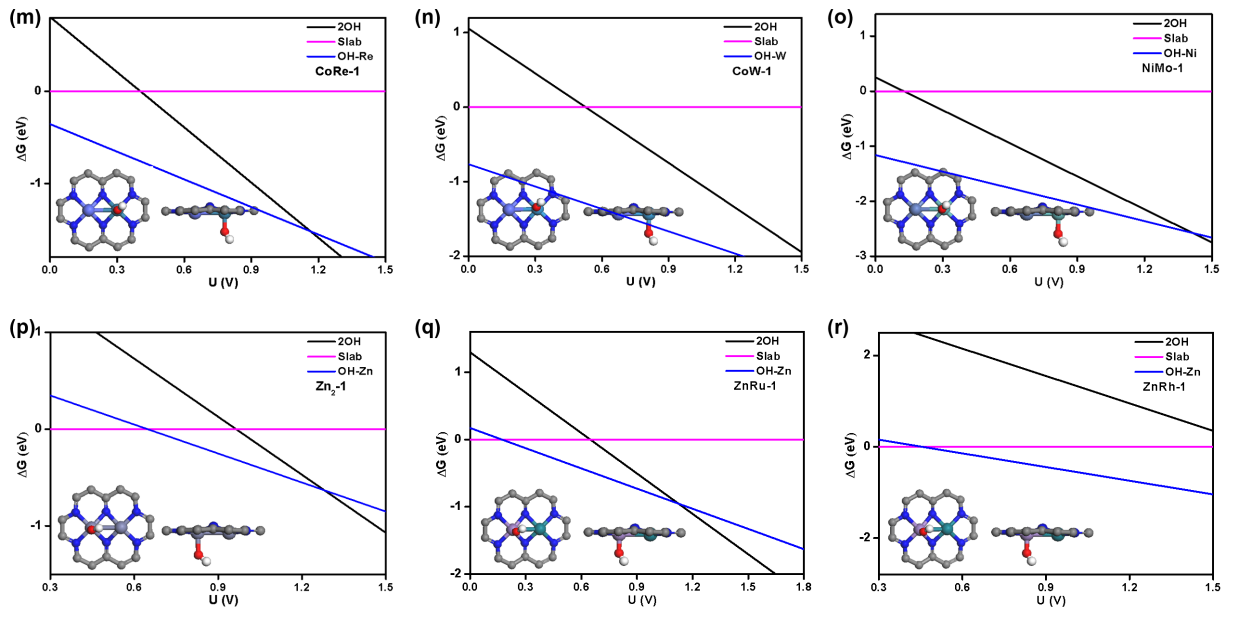


**Figure S6.** The pre-adsorption phase diagrams of M_1_M_2_-1 DACs.


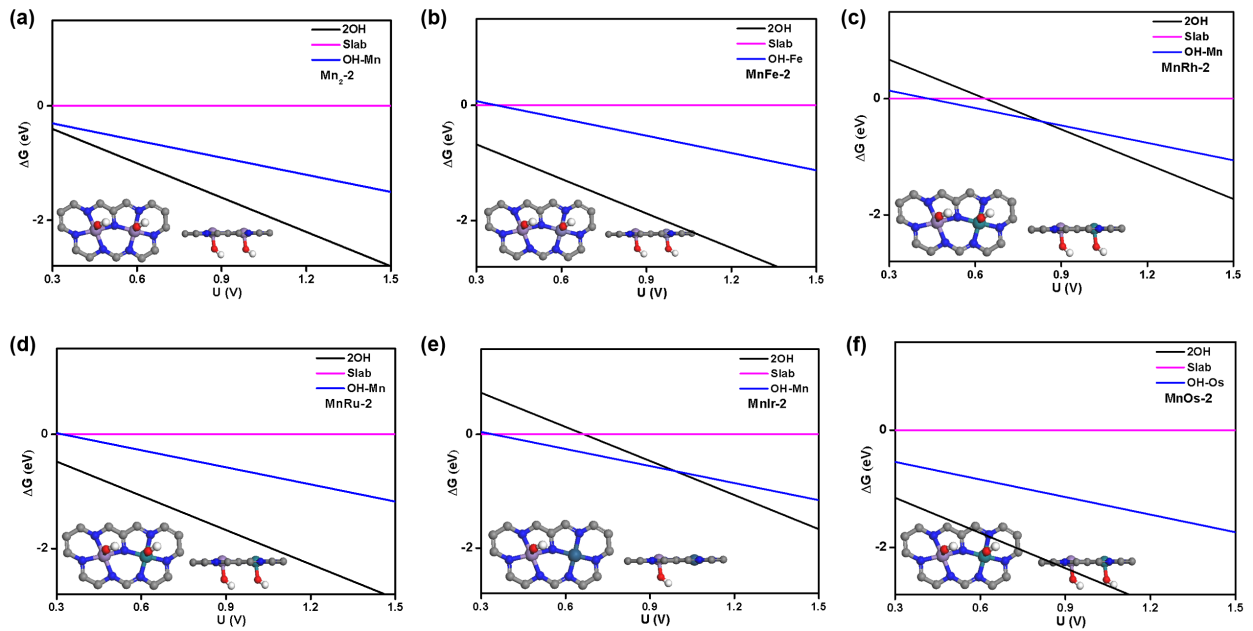


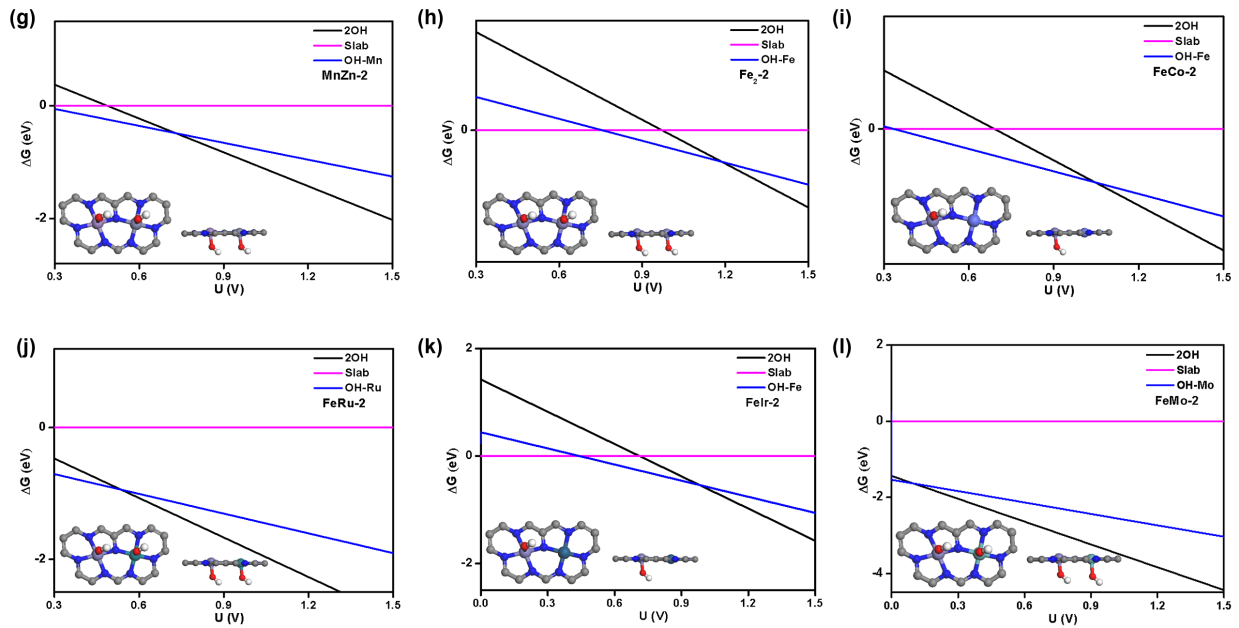


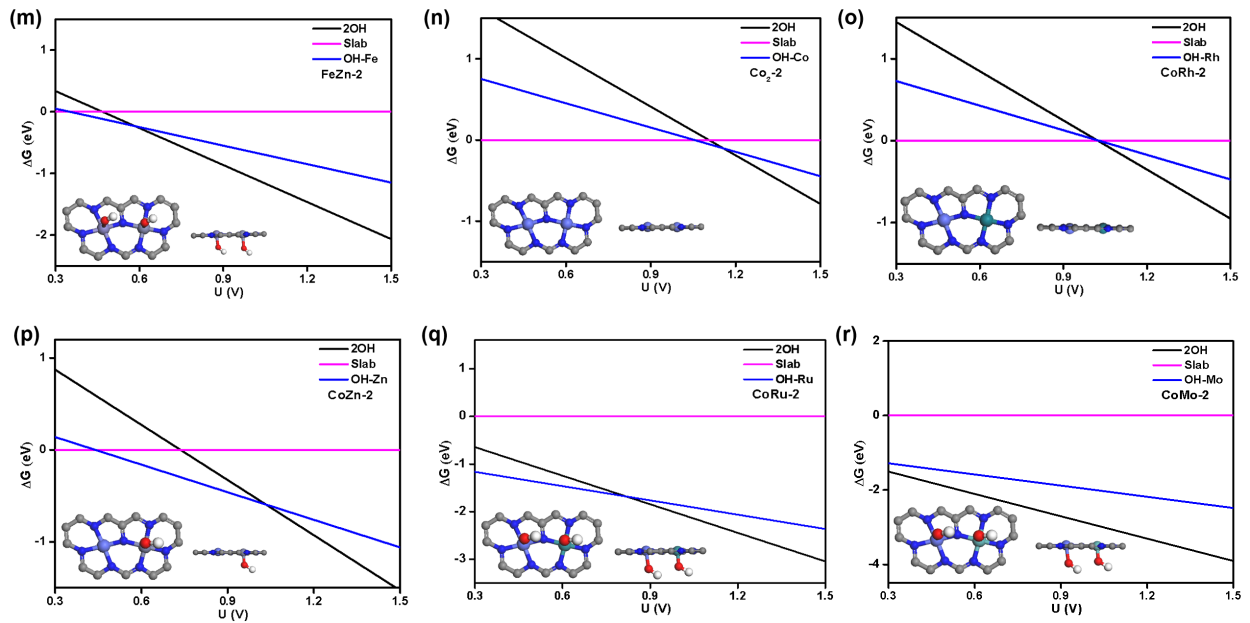


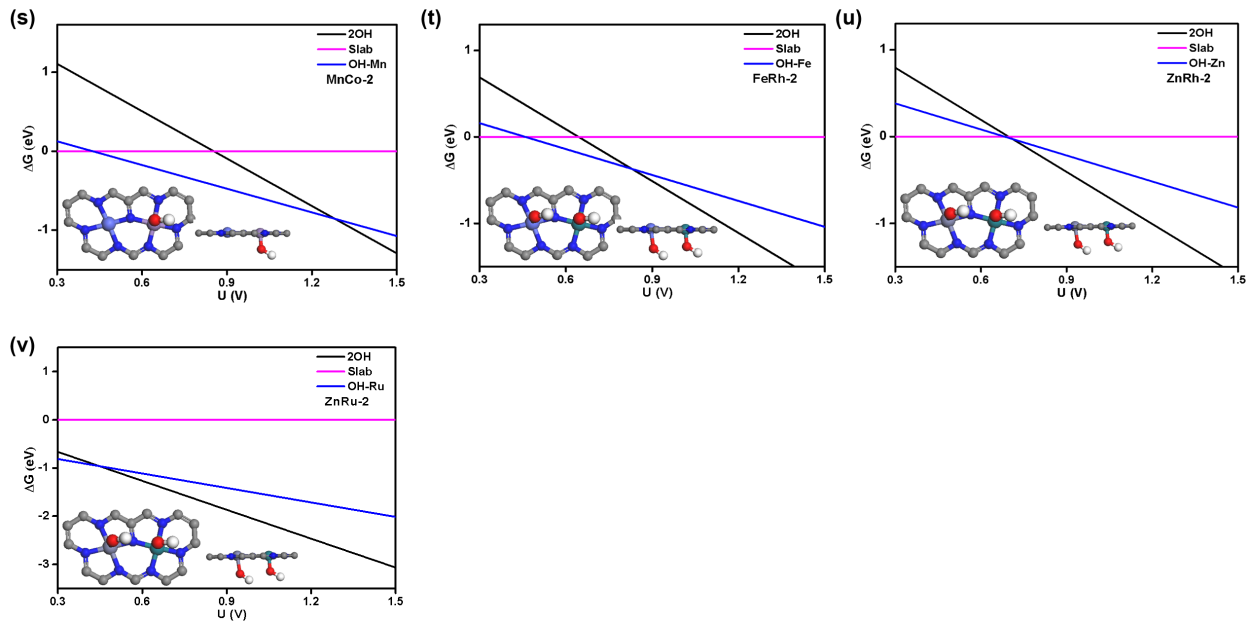


**Figure S7.** The pre-adsorption phase diagrams of M_1_M_2_-2 DACs.


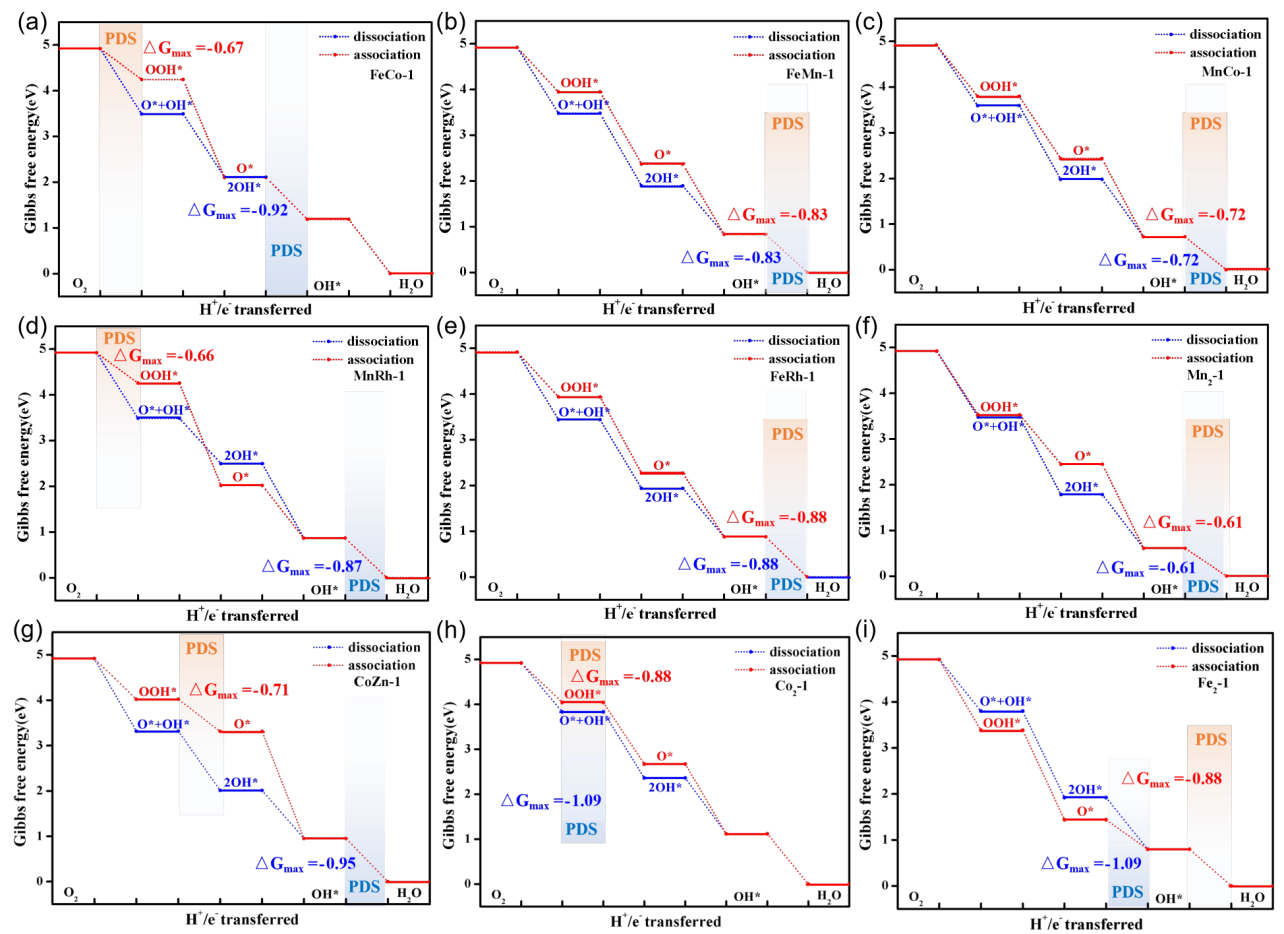


**Figure S8.** The ORR Gibbs free energy step diagrams of M_1_M_2_-1 DACs under the inverse dissociation mechanism and the corresponding association mechanism.


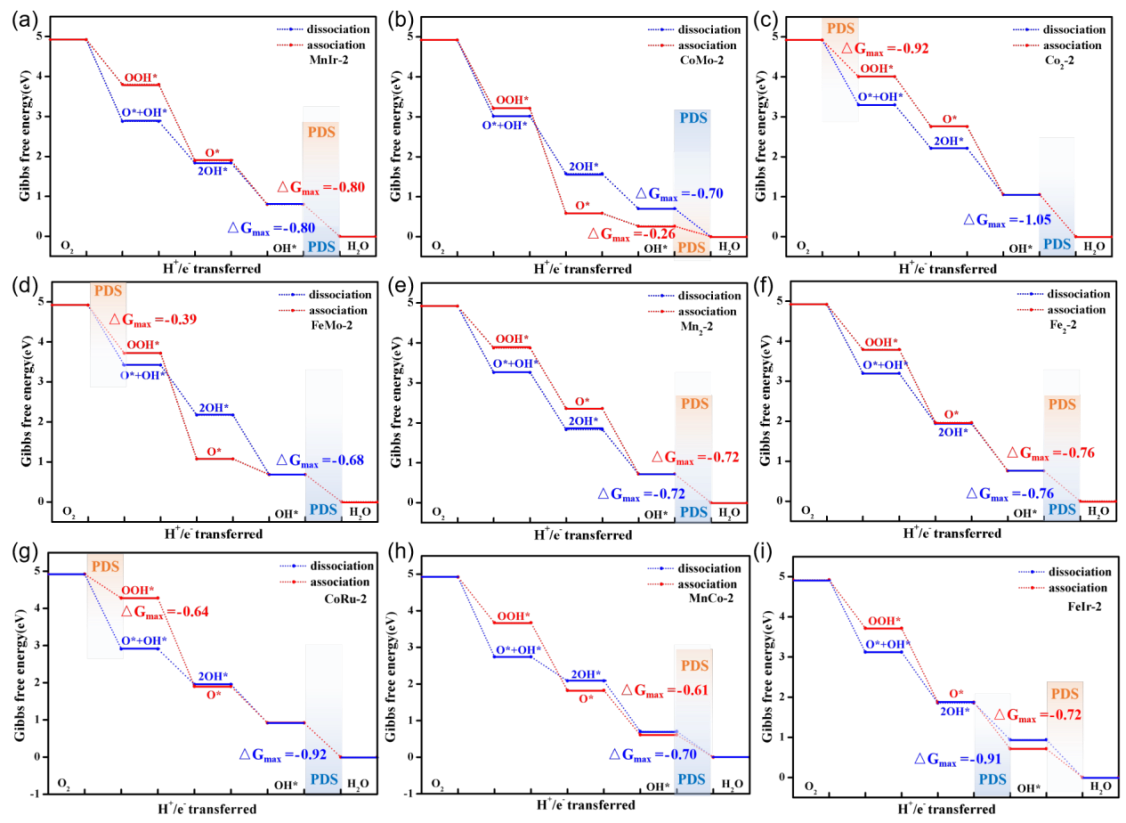


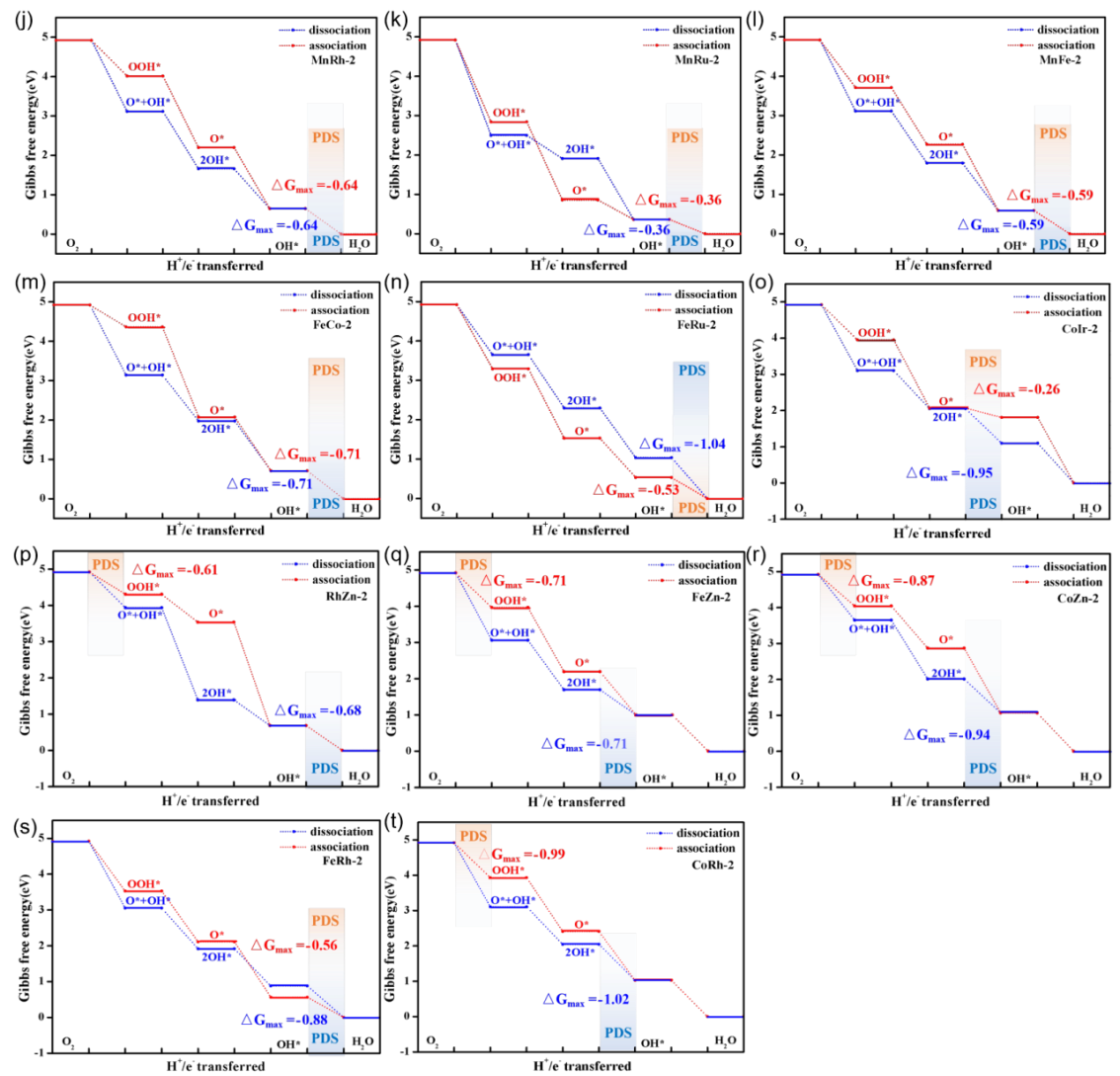


**Figure S9**. The ORR Gibbs free energy step diagrams of M_1_M_2_-2 DACs under the inverse dissociation mechanism and the corresponding association mechanism.


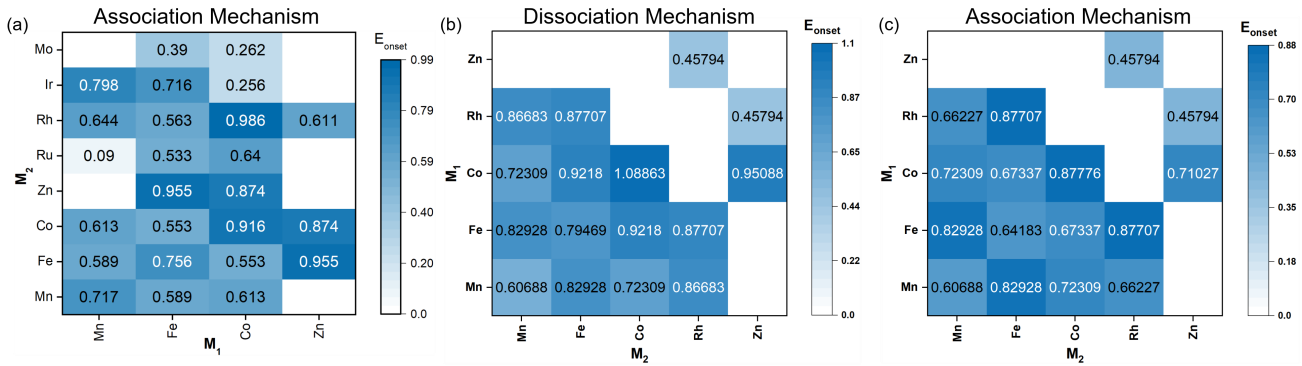


**Figure S10**. The heat map of ORR theoretical onset-potential through (a) association mechanism on M_1_M_2_-2, (b) dissociation mechanism on M_1_M_2_-1, and (c) association mechanism on M_1_M_2_-1.


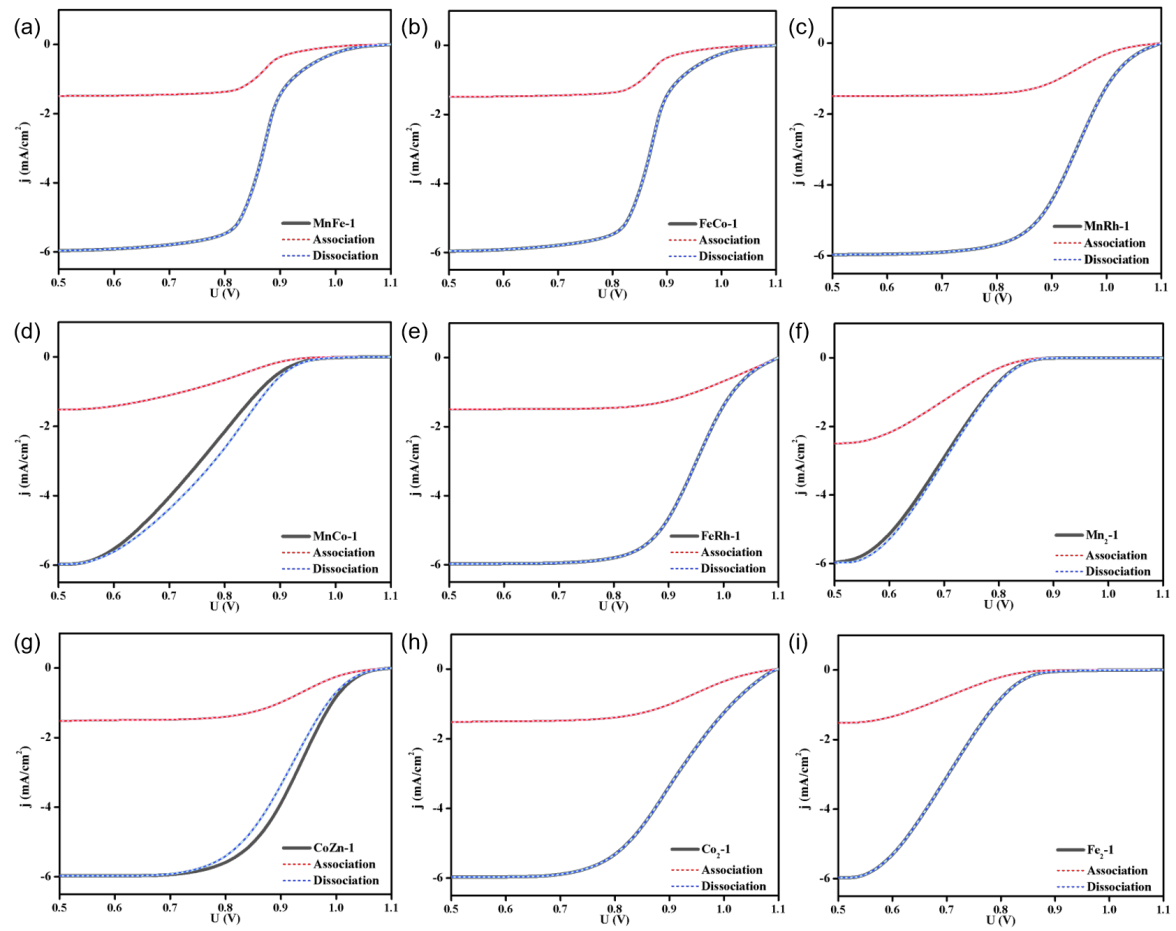


**Figure S11.** Simulated polarization curves of M_1_M_2_-1 DACs with constant charge method.


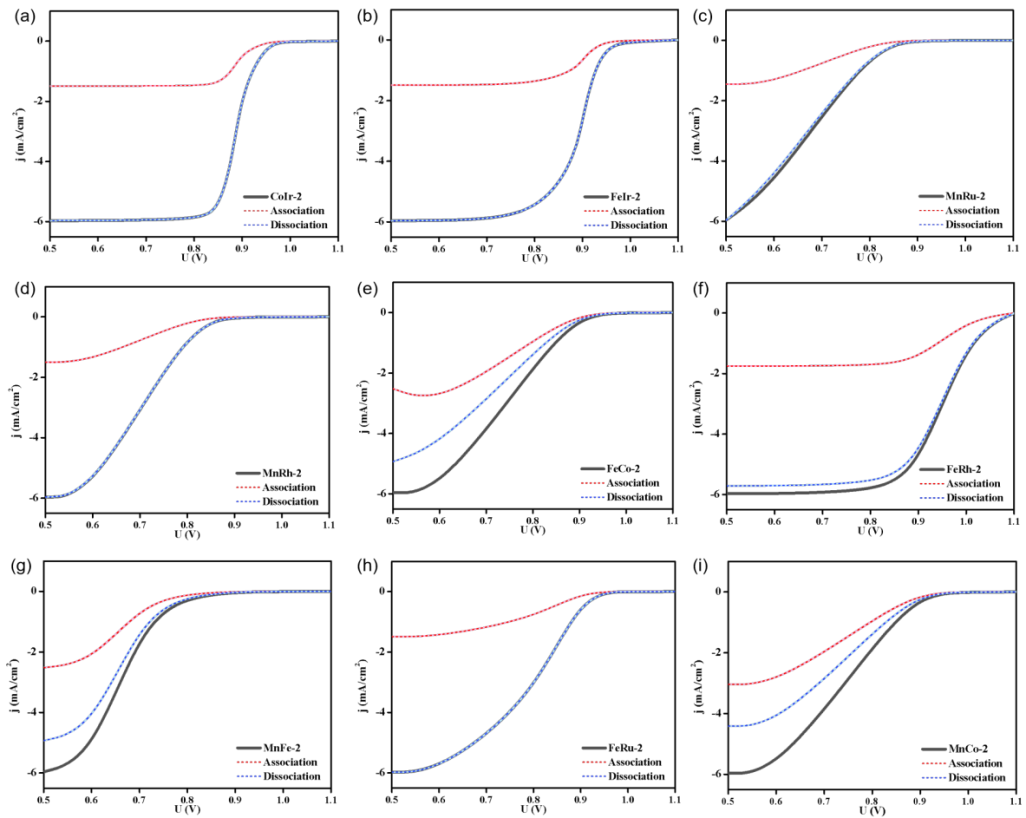


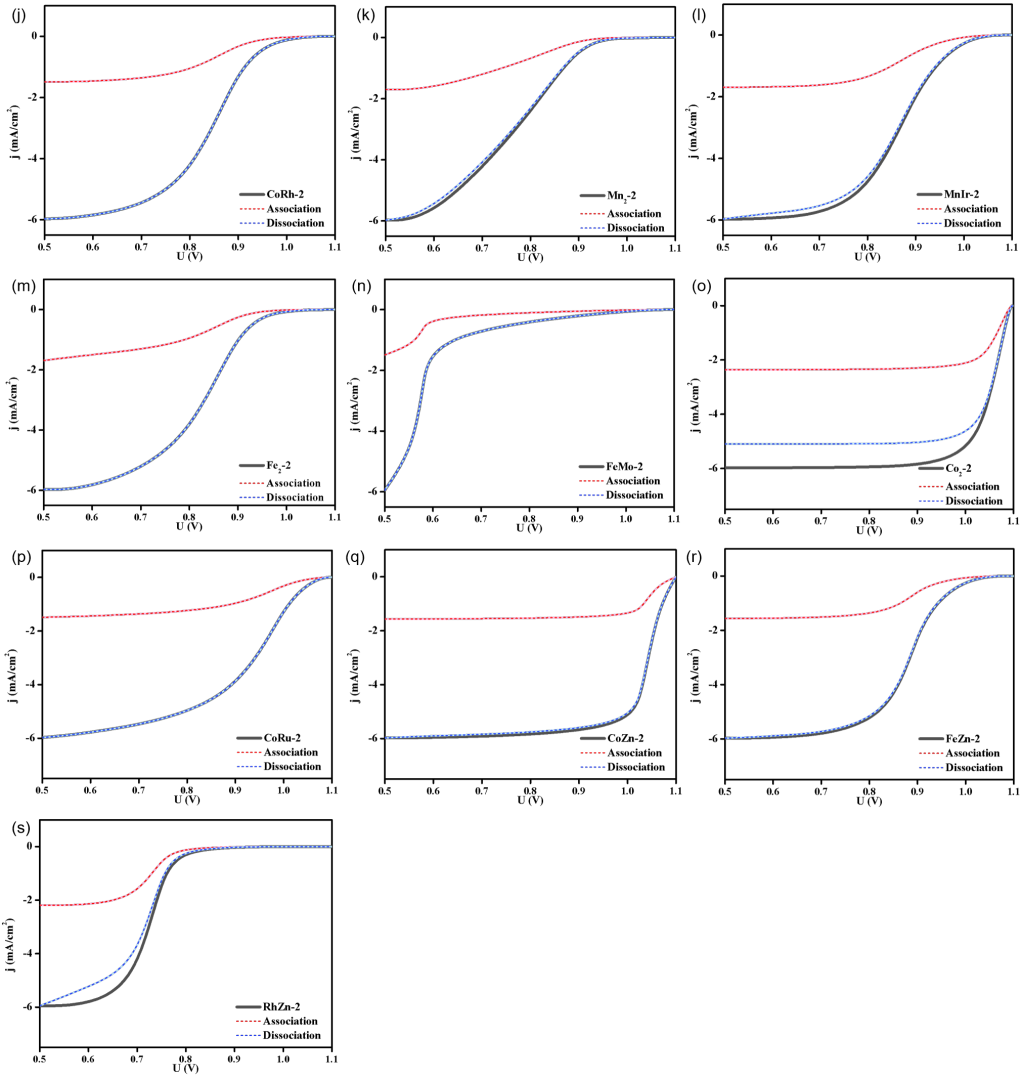


**Figure S12.** Simulated polarization curves of M_1_M_2_-2 DACs with constant charge method.


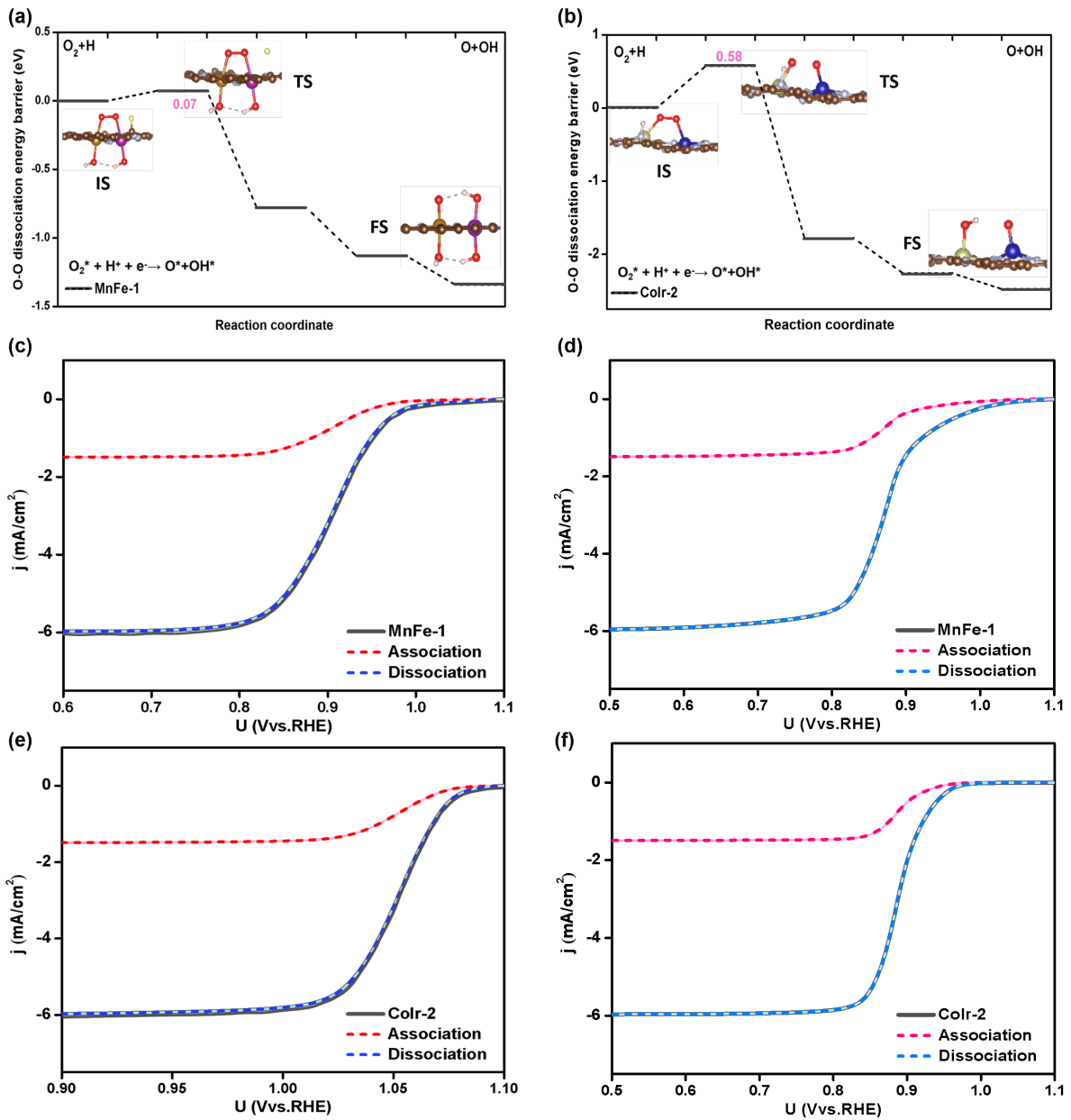


**Figure S13.** (a-b) The O-O bond cleavage barrier of O_2_* upon protonation from co-adsorbed H on MnFe-1 DAC and CoIr-2 DAC by CINEB. The polarization curves of (c) MnFe-1 and (e) CoIr-2 DAC were calculated with calculated dissociation barrier. The polarization curves of (d) MnFe-1 and (f) CoIr-2 DAC with an activation energy of 0.26 eV.


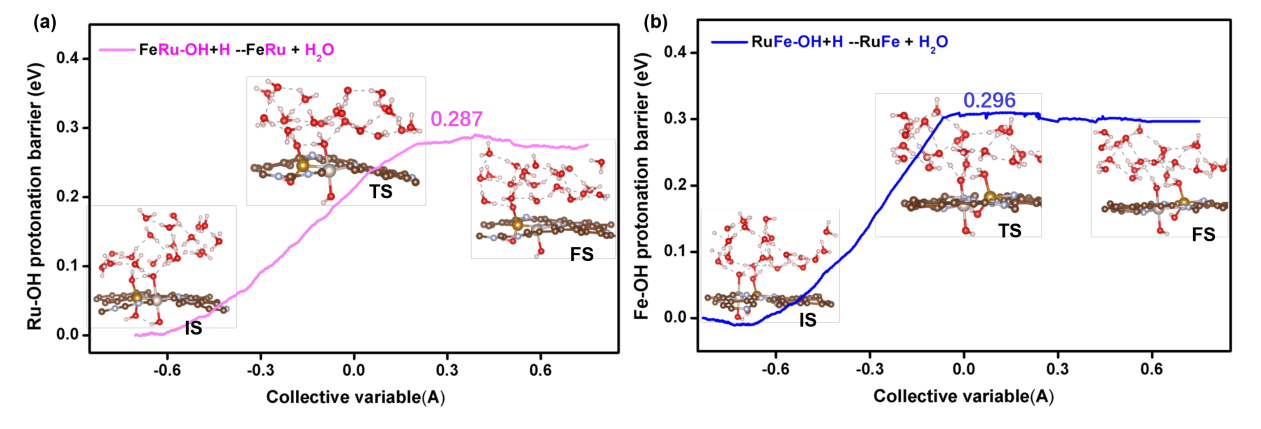


**Figure S14.** Free energy profile and average energy during the protonation process of 2OH at (a) the Fe site and (b) Ru site of the FeRu-2 DAC, respectively.


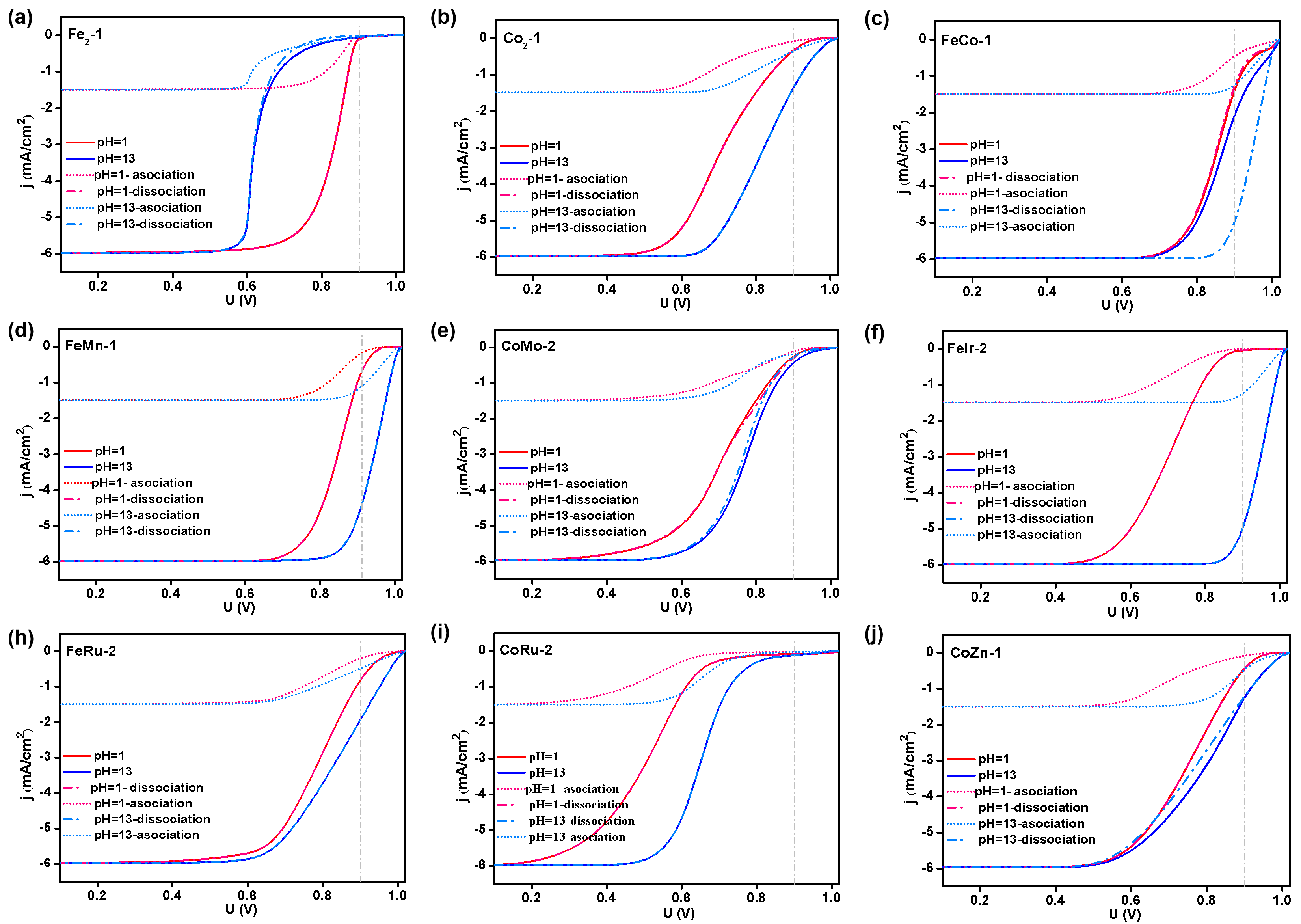


**Figure S15.** Simulated polarization curves of DACs at different potentials with pH=1 and 13 under the condition of constant potential.


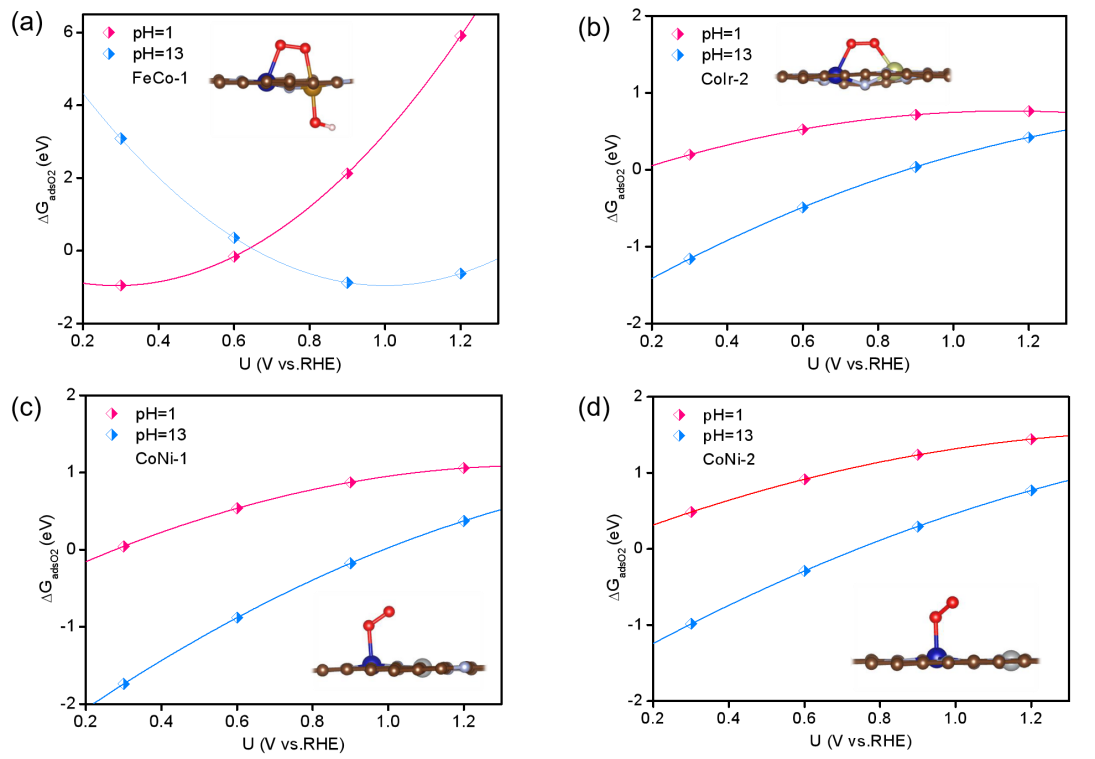


**Figure S16.** The variations in O_2_ adsorption energy (ΔG_adsO2_) and the corresponding optimized structural diagrams for four DACs during the ORR process, simulated using the potential-dependent method under different pH conditions.


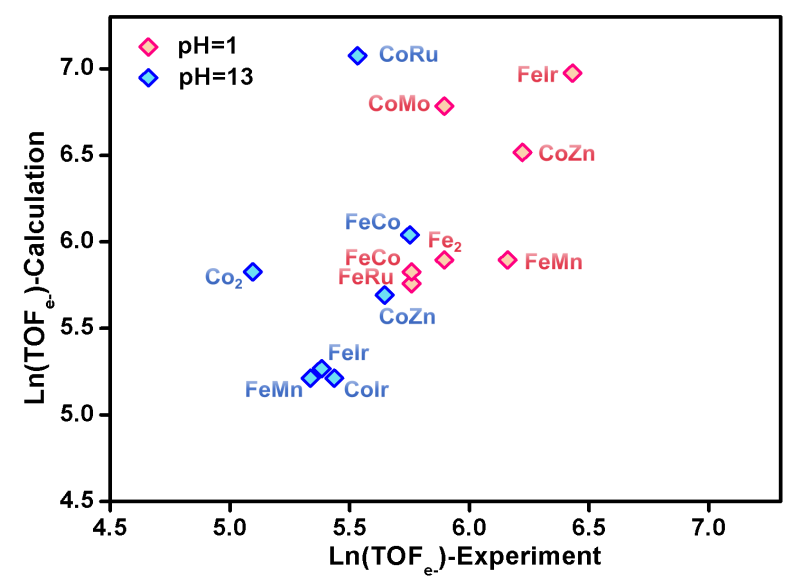


**Figure S17.** Under different pH conditions, the intrinsic turnover frequency (TOF_e-_) of DACs during the ORR process were simulated using the constant potential method and compared with the actual experimental values.


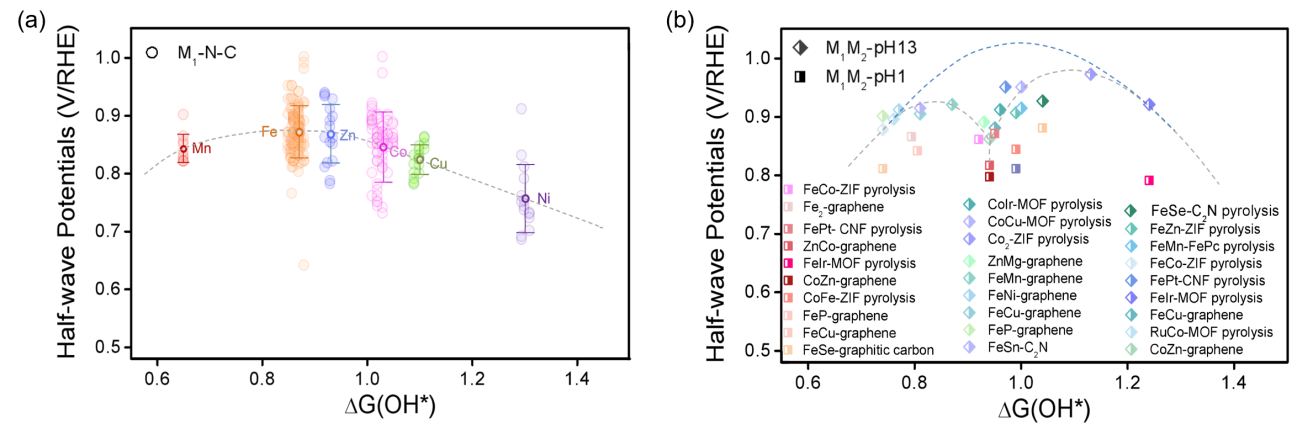


**Figure S18.** Experimental data indicate that ORR volcano plots for (a) SACs and (b) DAC exhibit a notable difference. The ORR volcano demonstrates a unique transition from (a) a single-peak for SACs to (b) a double-peak pattern for DACs. (The above data are available in the *DigCat* database: <https://www.digcat.org>).

**Figure S19**. Goodness-of-fit analysis for the single- and dual-peak volcano models


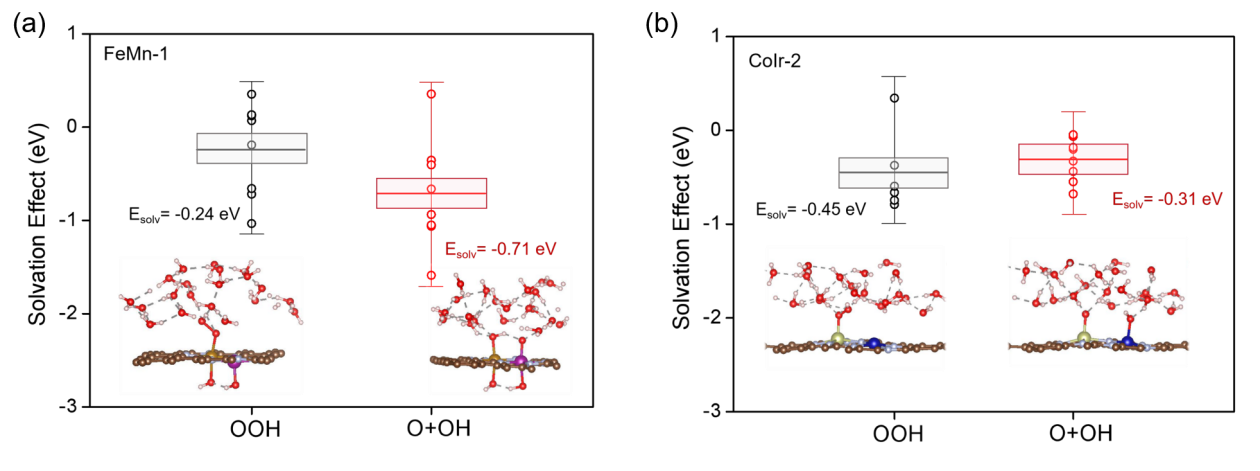


**Figure S20**. Binding energy changes of OH* and OOH* in the presence of explicit water molecules.


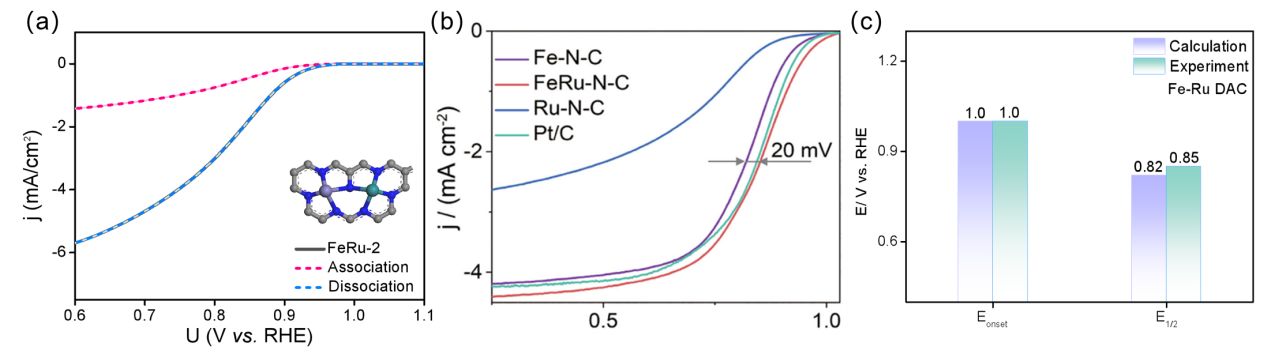


**Figure S21**. (a-b) Simulated and literature polarization curves^25^ of FeRu DAC; (c) Bar chart comparing calculated vs. experimental onset and half-wave potentials of FeRu DAC. Copy right 2024 Elsevier,ref 25.

**Section 4 Supplementary Tables**

**Table S1.** Comparison of ORR Performance for M_1_M_2_ DACs at pH = 1 (Related to Fig. 6c). DACs within the “performance trap” region near Δ*G*(OH*) ~ 0.9 eV are marked in red.

| **M_1_M_2_ DACs** | **DFT-OH*/eV** | U=0.9 V vs. RHE  **Ln(TOFe-)** | **E_1/2_/eV** | **Reference** |
| --- | --- | --- | --- | --- |
| FeMn | 0.83 | 4.21 | 0.804 | ***‌Nat Commun***.2021,12,1734‌ |
| Fe_2_ | 0.79 | 3.02 | 0.84 | ***Chem.*** 2019, 5, 2865–2878 |
| Fe_2_ | 0.79 | 4.49 | 0.865 | ***Angew. Chem. Int. Ed.*** *2025, 64, e202510671* |
| FeCo | 1.19 | 4.24 | 0.86 | ***J. Am. Chem. Soc.*** 2019, 141,  17763−17770 |
| FeIr | 0.94 | 3.12 | 0.77 | ***ACSCatal.***2022,12,9397−9409 |
| CoZn | 0.95 | 3.02 | 0.796 | ***Angew.Chem. Int. Ed.*** 2019, 58,2622–2626 |
| FeNi | 0.77 | 2.79 | 0.8 | ***J. Phys. Chem. Lett.*** 2020, 11, 1404−1410 |
| FeZn | 0.99 | 4.3 | 0.86 | ***Angew.Chem. Int. Ed.*** 2019, 58,2622–2626 |
| FeZn | 0.99 | 5.84 | 0.906 | ***Energy Environ. Sci.,*** *2022, 15, 1601–1610* |
| FeCu | 0.81 | 3.89 | 0.841 | ***ACS Catal.****2*026,published online |
| FeRu | 1.04 | 4.21 | 0.843 | ***Matter.***2024,7, 1517–1532 |
| CoPt | 0.84 | 4.18 | 0.841 | ***Adv. Mater.*** 2025, 37, 2507478 |

**Table S2.** Comparison of ORR Performance for M_1_M_2_ DACs at pH = 13 (Related to Fig. 6c). DACs within the “performance trap” region near Δ*G*(OH*) ~ 0.9 eV are marked in red.

| **M_1_M_2_ DACs** | **DFT-OH*/eV** | U=0.9 V vs. RHE  **Ln(TOFe-)** | **E_1/2_/eV** | **Reference** |
| --- | --- | --- | --- | --- |
| FeMn | 0.83 | 5.76 | 0.924 | ***Appl. Catal. B Environ.***2026,384,126191‌ |
| FeMn | 0.83 | 5.72 | 0.89 | ***Inorg. Chem.*** 2020, 59, 5194−5205 |
| FeMn | 0.83 | 5.75 | 0.928 | ***‌Nat Commun***.2021,12,1734 |
| Fe_2_ | 0.79 | 5.83 | 0.934 | ***Appl. Catal. B Environ.***2026,384,126191 |
| Fe_2_ | 0.79 | 5.67 | 0.92 | ***Angew. Chem. Int. Ed.*** *2023, 62, e202300826* |
| FeIr | 0.94 | 5.87 | 0.92 | ***ACSCatal.***2022,12,9397−9409 |
| CoZn | 0.95 | 5.02 | 0.861 | ***Angew.Chem. Int. Ed***. 2019, 58,2622–2626 |
| FeNi | 0.77 | 5.54 | 0.911 | ***Appl. Catal. B Environ.***2026,384,126191 |
| FeZn | 0.99 | 4.93 | 0.88 | ***Angew.Chem. Int. Ed***. 2019, 58,2622–2626 |
| FeZn | 0.99 | 3.71 | 0.81 | ***Energy Environ. Sci.,*** *2022, 15, 1601–1610* |
| FePt | 0.97 | 5.97 | 0.95 | ***‌Nat Commun***.2022,13,6414 |
| FeCu | 0.81 | 5.5 | 0.904 | ***Appl. Catal. B Environ.***2026,384,126191 |
| FeSe | 1.04 | 6.01 | 0.926 | ***Nature Commun.****2025,16,470* |
| CoCu | 1 | 5.97 | 0.895 | ***Adv. Funct. Mater.*** 2024, 34, 2311664 |
| CoIr | 1.10 | 5.86 | 0.911 | ***ACS Catal.*** 2021, 11, 8837−8846 |
| FeCo | 1.19 | 4.87 | 0.877 | ***ACS Catal.*** 2022, 12, 1216−1227 |
| FeCo | 1.19 | 5.69 | 0.92 | ***Appl. Catal. B Environ.***2026,384,126191 |
| FeCo | 1.19 | 4.18 | 0.86 | ***Electroanalysis*** 2022, 34,  1572–1578 |
| FeCo | 1.19 | 6.01 | 0.954 | ***Energy Environ. Sci.***, 2018, 11,  3375--3379 |
| CoRu | 0.92 | 4.81 | 0.895 | ***ACSNano.***2022,16,10657−10666 |
| Co_2_ | 1.06 | 6.11 | 0.972 | ***J. Am. Chem. Soc.*** 2024, 146, 35295−35304 |
| CoCu | 0.81 | 5.5 | 0.904 | ***Appl. Catal. B Environ.***2026,384,126191 |

**Table S3** The average Δ*E*_solv_ values of each system adsorbed with O* + OH*, OOH*, 2OH*, O*, and OH*.

| **The average Δ*E*_solv_** | **M_1_M_2_-1** | **M_1_M_2_-2** |
| --- | --- | --- |
| O*+OH* | -0.496 | -0.533 |
| OOH* | -0.292 | -0.364 |
| 2OH* | -0.470 | -0.449 |
| O* | -0.203 | -0.197 |
| OH* | -0.281 | -0.340 |

**Table S4.** The potential-dependent free energy expressions (*G*(U)) of each species in the M_1_M_2_-1 system, respectively.

| **System** | **Species** | ***G*(*U*) /eV (pH= 1)** | ***G*(*U*) /eV (pH= 13)** |
| --- | --- | --- | --- |
| FeCo-1 | Slab | 6.08U-8.49U^2^-438.03 | 8.14U-8.49U^2^-446.63 |
|  | O*+OH* | -4.10U+5.19 U^2^-451.64 | -6.48U+5.19U^2^-446.11 |
|  | 2OH* | 3.60U-0.96 U^2^-458.59 | 4.95U-0.96 U^2^-461.62 |
|  | OH* | -2.16U+6.99U^2^-447.33 | -4.10U+6.99U^2^-442.26 |
|  | OOH* | 3.30U-2.71 U^2^-451.72 | 7.15U-2.71 U^2^-455.44 |
|  | O* | -7.09U+5.35U^2^-419.79 | -9.69U+5.35U^2^-422.41 |
| FeMn-1 | Slab | 5.22U-4.41U^2^-451.96 | 7.48U-4.41U^2^-457.89 |
|  | O*+OH* | 2.65U-11.92U^2^-479.62 | 4.35U-11.92 U^2^-485.49 |
|  | 2OH* | 2.22U-0.23 U^2^-471.57 | 2.55U-0.23 U^2^-473.26 |
|  | OH* | 3.59U-0.84U^2^-462.60 | 4.79U-0.84U^2^-465.57 |
|  | OOH* | 8.96U-4.60U^2^-467.93 | 11.50U-4.60 U^2^-476.62 |
|  | O* | 2.99U-2.03 U^2^-466.08 | 5.88U-2.03 U^2^-477.63 |
| Co_2_-1 | Slab | 0.91U-3.02U^2^-425.28 | 5.20U-3.02U^2^-427.45 |
|  | O*+OH* | 4.23U-0.88 U^2^-447.17 | 5.49U-0.88U^2^-450.62 |
|  | 2OH* | 3.75U-0.81 U^2^-451.63 | 4.90U-0.81 U^2^-454.70 |
|  | OH* | 3.33U-2.10U^2^-441.08 | 6.31U-2.10U^2^-444.51 |
|  | OOH* | 2.58U-1.06 U^2^-445.04 | 4.08U-1.06U^2^-447.41 |
|  | O* | 4.29U-1.79 U^2^-436.67 | 6.83U-1.79 U^2^-440.63 |
| Fe_2_-1 | Slab | 3.10U-0.87U^2^-454.17 | 4.33U-0.87U^2^-456.81 |
|  | O*+OH* | 1.26U-4.93 U^2^-470.87 | 1.96U-4.93 U^2^-482.30 |
|  | 2OH* | 1.26U+1.76 U^2^-469.48 | -1.24U+1.76 U^2^-469.48 |
|  | OH* | 5.19U-2.74U^2^-460.86 | 7.08U-2.74U^2^-465.93 |
|  | OOH* | 3.50U-0.69 U^2^-464.51 | 4.48U-0.69 U^2^-467.34 |
|  | O* | 4.29U-1.79 U^2^-454.67 | 6.83U-1.79 U^2^-458.63 |
| CoZn-1 | Slab | 0.34U-3.50U^2^-420.99 | 5.31U-3.50U^2^-422.99 |
|  | O*+OH* | 4.29U-0.47 U^2^-438.84 | 4.96U-0.47U^2^-442.13 |
|  | 2OH* | 3.54U-0.28U^2^-443.70 | 3.94U-0.28U^2^-446.36 |
|  | OH* | 4.62U-2.02U^2^-433.00 | 7.48U-2.02U^2^-437.30 |
|  | OOH* | 4.20U-1.24U^2^-427.61 | 5.96U-1.24 U^2^-431.22 |
|  | O* | 2.47U-1.12 U^2^-436.30 | 4.06U-1.12 U^2^-438.62 |

**Table S5.** The potential-dependent free energy expressions (*G*(U)) of each species in the M_1_M_2_-2 system, respectively.

| **System** | **Species** | ***G*(*U*) /eV (pH= 1)** | ***G*(*U*) /eV (pH= 13)** |
| --- | --- | --- | --- |
| CoRh-2 | Slab | -1.40U+1.24U^2^-426.79 | -3.16U+1.24U^2^-424.98 |
|  | O*+OH* | -0.20U-0.03U^2^-441.93 | -0.16U-0.03U^2^-441.80 |
|  | 2OH* | -0.11U-0.12U^2^-446.50 | 0.05U-0.12 U^2^-446.47 |
|  | OH* | 0.008U-0.41U^2^-436.85 | 0.59U-0.41U^2^-437.06 |
|  | OOH* | -0.55U+0.25 U^2^-440.54 | -0.91U+0.25U^2^-440.02 |
|  | O* | 0.04U-0.50U^2^-431.92 | 0.75U-0.50U^2^-432.20 |
| CoIr-2 | Slab | -0.008U-0.31U^2^-428.17 | 0.44U-0.31U^2^-428.32 |
|  | O*+OH* | 0.22U-0.30 U^2^-443.25 | 0.65U-0.30 U^2^-443.56 |
|  | 2OH* | 0.01U-0.27 U^2^-447.69 | 0.40U-0.27 U^2^-447.84 |
|  | OH* | 0.16U-0.32U^2^-437.90 | 0.62U-0.32U^2^-438.17 |
|  | OOH* | -0.85U+0.18 U^2^-442.44 | -1.10U+0.18U^2^-441.74 |
|  | O* | -0.02U-0.22U^2^-433.51 | 0.29U-0.22U^2^-433.60 |
| CoRu-2 | Slab | 2.04U-3.05U^2^-434.41 | 6.38U-3.05U^2^-437.40 |
|  | O*+OH* | 4.27U-1.42U^2^-451.51 | 6.29U-1.42U^2^-455.26 |
|  | 2OH* | 3.31U-1.74U^2^-454.73 | 5.78U-1.74U^2^-457.96 |
|  | OH* | 4.10U-2.08U^2^-445.40 | 7.06U-2.08U^2^-449.36 |
|  | OOH* | 3.64U-1.09 U^2^-449.35 | 5.19U-1.09 U^2^-452.79 |
|  | O* | 4.18U-3.01 U^2^-440.88 | 8.46U-3.01 U^2^-445.89 |
| FeRu-2 | Slab | 3.55U-1.81U^2^-455.78 | 6.12U-1.81U^2^-459.41 |
|  | O*+OH* | 6.23U-2.38U^2^-477.77 | 9.61U-2.38U^2^-483.40 |
|  | 2OH* | 4.30U-0.72U^2^-481.77 | 5.33U-0.72U^2^-485.19 |
|  | OH* | 3.92U-0.38U^2^-472.04 | 4.46U-0.38U^2^-475.02 |
|  | OOH* | 2.51U-0.92U^2^-466.62 | 3.82U-0.92U^2^-468.87 |
|  | O* | 3.61U-0.75U^2^-476.08 | 4.76U-0.75U^2^-479.30 |
| FeIr-2 | Slab | 3.74U-1.44U^2^-441.07 | 5.79U-1.44U^2^-444.46 |
|  | O*+OH* | -6.29U+7.25 U^2^-442.47 | -6.58U+7.25U^2^-446.12 |
|  | 2OH* | 1.10U-2.54U^2^-450.44 | 4.72U-2.54U^2^-471.11 |
|  | OH* | 3.59U-3.24U^2^-450.45 | 8.19U-3.24U^2^-454.63 |
|  | OOH* | 2.61U-2.34 U^2^-454.70 | 6.01U-2.34U^2^-457.76 |
|  | O* | 4.15U-3.47U^2^-445.98 | 9.08U-3.47U^2^-450.68 |
| CoMo-2 | Slab | 1.52U-0.78U^2^-439.17 | 2.63U-0.78U^2^-440.65 |
|  | O*+OH* | 3.11U-1.10U^2^-440.65 | 4.66U-1.10U^2^-443.41 |
|  | 2OH* | 2.31U-0.95U^2^-459.57 | 3.67U-0.95U^2^-461.69 |
|  | OH* | 1.01U-0.03U^2^-449.52 | 1.05U-0.03U^2^-450.25 |
|  | OOH* | 1.15U-0.18U^2^-453.84 | 1.41U-0.18U^2^-454.75 |
|  | O* | 1.40U-0.30U^2^-444.60 | 1.82U-0.30U^2^-445.74 |

**Table S6** Raw data set of nine eigenvalues of 105 M_1_M_2_-1 DACs. The systems that can form side-on adsorption with O_2_ are marked in blue.

|  | R_M1_ | R_M2_ | E_M1_ | E_M2_ | N_M1_ | N_M2_ | Mean-N_M1/M2_ | Bond_M12_ | N_num_ | L_O2_ |
| --- | --- | --- | --- | --- | --- | --- | --- | --- | --- | --- |
| Mn_2_ | 1.17 | 1.17 | -4.37 | -4.37 | 1.55 | 1.55 | 1.55 | 2.306 | 6 | 1.323 |
| MnFe | 1.17 | 1.17 | -4.37 | -2.02 | 1.55 | 1.83 | 1.69 | 2.405 | 6 | 1.326 |
| MnCo | 1.17 | 1.16 | -4.37 | -0.31 | 1.55 | 1.88 | 1.72 | 2.508 | 6 | 1.326 |
| MnNi | 1.17 | 1.15 | -4.37 | 0.43 | 1.55 | 1.91 | 1.73 | 2.533 | 6 | 1.29 |
| MnCu | 1.17 | 1.17 | -4.37 | 0.62 | 1.55 | 1.9 | 1.72 | 2.559 | 6 | 1.282 |
| MnZr | 1.17 | 1.45 | -4.37 | -6.46 | 1.55 | 1.33 | 1.44 | 2.671 | 6 | 1.355 |
| MnNb | 1.17 | 1.34 | -4.37 | -8.76 | 1.55 | 1.6 | 1.58 | 2.51 | 6 | 1.5 |
| MnMo | 1.17 | 1.30 | -4.37 | -1.4 | 1.55 | 2.16 | 1.86 | 2.361 | 6 | 1.303 |
| MnRu | 1.17 | 1.25 | -4.37 | 0.61 | 1.55 | 2.2 | 1.88 | 2.336 | 6 | 1.36 |
| MnRh | 1.17 | 1.25 | -4.37 | 1.32 | 1.55 | 2.28 | 1.92 | 2.359 | 6 | 1.324 |
| MnPd | 1.17 | 1.28 | -4.37 | 0.92 | 1.55 | 2.2 | 1.88 | 2.567 | 6 | 1.281 |
| MnAg | 1.17 | 1.34 | -4.37 | 1.60 | 1.55 | 1.93 | 1.74 | 2.662 | 6 | 1.298 |
| MnHf | 1.17 | 1.44 | -4.37 | -7.08 | 1.55 | 1.3 | 1.42 | 2.731 | 6 | 1.303 |
| MnTa | 1.17 | 1.34 | -4.37 | -10.93 | 1.55 | 1.5 | 1.52 | 2.51 | 6 | 1.508 |
| MnW | 1.17 | 1.3 | -4.37 | -1.18 | 1.55 | 2.36 | 1.96 | 2.361 | 6 | 1.509 |
| MnRe | 1.17 | 1.28 | -4.37 | -1.07 | 1.55 | 1.9 | 1.72 | 2.338 | 6 | 1.409 |
| MnOs | 1.17 | 1.26 | -4.37 | 2.32 | 1.55 | 2.2 | 1.88 | 2.361 | 6 | 1.377 |
| MnIr | 1.17 | 1.27 | -4.37 | 1.2 | 1.55 | 2.2 | 1.88 | 2.366 | 6 | 1.346 |
| MnPt | 1.17 | 1.3 | -4.37 | 2.2 | 1.55 | 2.28 | 1.92 | 2.582 | 6 | 1.28 |
| MnAu | 1.17 | 1.34 | -4.37 | 5.22 | 1.55 | 2.54 | 2.04 | 2.648 | 6 | 1.269 |
| Fe_2_ | 1.17 | 1.17 | -2.02 | -2.02 | 1.83 | 1.83 | 1.83 | 2.335 | 6 | 1.307 |
| FeCo | 1.17 | 1.16 | -2.02 | -0.31 | 1.83 | 1.88 | 1.86 | 2.378 | 6 | 1.322 |
| FeNi | 1.17 | 1.15 | -2.02 | 0.43 | 1.83 | 1.91 | 1.87 | 2.493 | 6 | 1.275 |
| FeCu | 1.17 | 1.17 | -2.02 | 0.62 | 1.83 | 1.9 | 1.87 | 2.531 | 6 | 1.276 |
| FeZr | 1.17 | 1.45 | -2.02 | -6.46 | 1.83 | 1.33 | 1.58 | 2.585 | 6 | 1.379 |
| FeNb | 1.17 | 1.34 | -2.02 | -8.76 | 1.83 | 1.6 | 1.72 | 2.425 | 6 | 1.498 |
| FeMo | 1.17 | 1.3 | -2.02 | -1.4 | 1.83 | 2.16 | 2.00 | 2.356 | 6 | 1.444 |
| FeRu | 1.17 | 1.25 | -2.02 | 0.61 | 1.83 | 2.2 | 2.02 | 2.323 | 6 | 1.428 |
| FeRh | 1.17 | 1.25 | -2.02 | 1.32 | 1.83 | 2.28 | 2.06 | 2.343 | 6 | 1.439 |
| FePd | 1.17 | 1.28 | -2.02 | 0.92 | 1.83 | 2.2 | 2.02 | 2.536 | 6 | 1.33 |
| FeAg | 1.17 | 1.34 | -2.02 | 1.6 | 1.83 | 1.93 | 1.88 | 2.673 | 6 | 1.274 |
| FeHf | 1.17 | 1.44 | -2.02 | -7.08 | 1.83 | 1.3 | 1.56 | 2.634 | 6 | 1.297 |
| FeTa | 1.17 | 1.34 | -2.02 | -10.93 | 1.83 | 1.5 | 1.66 | 2.524 | 6 | 1.51 |
| FeW | 1.17 | 1.30 | -2.02 | -1.18 | 1.83 | 2.36 | 2.10 | 2.363 | 6 | 1.51 |
| FeRe | 1.17 | 1.28 | -2.02 | -1.07 | 1.83 | 1.9 | 1.86 | 2.338 | 6 | 1.432 |
| FeOs | 1.17 | 1.26 | -2.02 | 2.32 | 1.83 | 2.2 | 2.02 | 2.325 | 6 | 1.388 |
| FeIr | 1.17 | 1.27 | -2.02 | 1.2 | 1.83 | 2.2 | 2.02 | 2.339 | 6 | 1.336 |
| FePt | 1.17 | 1.30 | -2.02 | 2.2 | 1.83 | 2.28 | 2.05 | 2.541 | 6 | 1.293 |
| FeAu | 1.17 | 1.34 | -2.02 | 5.22 | 1.83 | 2.54 | 2.18 | 2.709 | 6 | 1.296 |
| Co_2_ | 1.16 | 1.16 | -0.31 | -0.31 | 1.88 | 1.88 | 1.88 | 2.274 | 6 | 1.32 |
| CoNi | 1.16 | 1.15 | -0.31 | 0.43 | 1.88 | 1.91 | 1.90 | 2.618 | 6 | 1.281 |
| CoCu | 1.16 | 1.17 | -0.31 | 0.62 | 1.88 | 1.90 | 1.89 | 2.648 | 6 | 1.285 |
| CoZr | 1.16 | 1.45 | -0.31 | -6.46 | 1.88 | 1.33 | 1.60 | 2.552 | 6 | 1.337 |
| CoNb | 1.16 | 1.34 | -0.31 | -8.76 | 1.88 | 1.60 | 1.74 | 2.455 | 6 | 1.23 |
| CoMo | 1.16 | 1.30 | -0.31 | -1.4 | 1.88 | 2.16 | 2.02 | 2.343 | 6 | 1.246 |
| CoRu | 1.16 | 1.25 | -0.31 | 0.61 | 1.88 | 2.2 | 2.04 | 2.322 | 6 | 1.296 |
| CoRh | 1.16 | 1.25 | -0.31 | 1.32 | 1.88 | 2.28 | 2.08 | 2.332 | 6 | 1.285 |
| CoPd | 1.16 | 1.28 | -0.31 | 0.92 | 1.88 | 2.2 | 2.04 | 2.654 | 6 | 1.281 |
| CoAg | 1.16 | 1.34 | -0.31 | 1.6 | 1.88 | 1.93 | 1.90 | 2.694 | 6 | 1.292 |
| CoHf | 1.16 | 1.44 | -0.31 | -7.08 | 1.88 | 1.3 | 1.59 | 2.647 | 6 | 1.233 |
| CoTa | 1.16 | 1.34 | -0.31 | -10.93 | 1.88 | 1.5 | 1.69 | 2.516 | 6 | 1.513 |
| CoW | 1.16 | 1.3 | -0.31 | -1.18 | 1.88 | 2.36 | 2.12 | 2.35 | 6 | 1.472 |
| CoRe | 1.16 | 1.28 | -0.31 | -1.07 | 1.88 | 1.9 | 1.89 | 2.342 | 6 | 1.41 |
| CoOs | 1.16 | 1.26 | -0.31 | 2.32 | 1.88 | 2.2 | 2.04 | 2.333 | 6 | 1.22 |
| CoIr | 1.16 | 1.27 | -0.31 | 1.2 | 1.88 | 2.2 | 2.04 | 2.338 | 6 | 1.256 |
| CoPt | 1.16 | 1.30 | -0.31 | 2.2 | 1.88 | 2.28 | 2.08 | 2.696 | 6 | 1.266 |
| CoAu | 1.16 | 1.34 | -0.31 | 5.22 | 1.88 | 2.54 | 2.21 | 2.718 | 6 | 1.271 |
| Ni_2_ | 1.15 | 1.15 | 0.43 | 0.43 | 1.91 | 1.91 | 1.91 | 2.63 | 6 | 1.241 |
| NiCu | 1.15 | 1.17 | 0.43 | 0.62 | 1.91 | 1.9 | 1.90 | 2.647 | 6 | 1.244 |
| NiZr | 1.15 | 1.45 | 0.43 | -6.46 | 1.91 | 1.33 | 1.62 | 2.655 | 6 | 1.284 |
| NiNb | 1.15 | 1.34 | 0.43 | -8.76 | 1.91 | 1.6 | 1.76 | 2.542 | 6 | 1.319 |
| NiMo | 1.15 | 1.3 | 0.43 | -1.4 | 1.91 | 2.16 | 2.04 | 2.43 | 6 | 1.449 |
| NiRu | 1.15 | 1.25 | 0.43 | 0.61 | 1.91 | 2.2 | 2.06 | 2.363 | 6 | 1.306 |
| NiRh | 1.15 | 1.25 | 0.43 | 1.32 | 1.91 | 2.28 | 2.10 | 2.491 | 6 | 1.288 |
| NiPd | 1.15 | 1.28 | 0.43 | 0.92 | 1.91 | 2.2 | 2.06 | 2.683 | 6 | 1.241 |
| NiAg | 1.15 | 1.34 | 0.43 | 1.6 | 1.91 | 1.93 | 1.92 | 2.69 | 6 | 1.25 |
| NiHf | 1.15 | 1.44 | 0.43 | -7.08 | 1.91 | 1.3 | 1.60 | 2.658 | 6 | 1.307 |
| NiTa | 1.15 | 1.34 | 0.43 | -10.93 | 1.91 | 1.5 | 1.70 | 2.543 | 6 | 1.513 |
| NiW | 1.15 | 1.3 | 0.43 | -1.18 | 1.91 | 2.36 | 2.14 | 2.413 | 6 | 1.497 |
| NiRe | 1.15 | 1.28 | 0.43 | -1.07 | 1.91 | 1.90 | 1.90 | 2.395 | 6 | 1.341 |
| NiOs | 1.15 | 1.26 | 0.43 | 2.32 | 1.91 | 2.2 | 2.06 | 2.38 | 6 | 1.247 |
| NiIr | 1.15 | 1.27 | 0.43 | 1.2 | 1.91 | 2.2 | 2.06 | 2.507 | 6 | 1.245 |
| NiPt | 1.15 | 1.3 | 0.43 | 2.2 | 1.91 | 2.28 | 2.09 | 2.701 | 6 | 1.239 |
| NiAu | 1.15 | 1.34 | 0.43 | 5.22 | 1.91 | 2.54 | 2.22 | 2.714 | 6 | 1.248 |
| Cu_2_ | 1.17 | 1.17 | 0.62 | 0.62 | 1.90 | 1.90 | 1.90 | 2.687 | 6 | 1.251 |
| CuZr | 1.17 | 1.45 | 0.62 | -6.46 | 1.90 | 1.33 | 1.62 | 2.684 | 6 | 1.317 |
| CuNb | 1.17 | 1.34 | 0.62 | -8.76 | 1.90 | 1.60 | 1.75 | 2.567 | 6 | 1.332 |
| CuMo | 1.17 | 1.3 | 0.62 | -1.4 | 1.90 | 2.16 | 2.03 | 2.457 | 6 | 1.474 |
| CuRu | 1.17 | 1.25 | 0.62 | 0.61 | 1.90 | 2.2 | 2.05 | 2.384 | 6 | 1.303 |
| CuRh | 1.17 | 1.25 | 0.62 | 1.32 | 1.90 | 2.28 | 2.09 | 2.497 | 6 | 1.282 |
| CuPd | 1.17 | 1.28 | 0.62 | 0.92 | 1.90 | 2.2 | 2.05 | 2.688 | 6 | 1.244 |
| CuAg | 1.17 | 1.34 | 0.62 | 1.6 | 1.90 | 1.93 | 1.92 | 2.734 | 6 | 1.255 |
| CuHf | 1.17 | 1.44 | 0.62 | -7.08 | 1.90 | 1.3 | 1.6 | 2.69 | 6 | 1.465 |
| CuW | 1.17 | 1.3 | 0.62 | -1.18 | 1.90 | 2.36 | 2.13 | 2.472 | 6 | 1.497 |
| CuRe | 1.17 | 1.28 | 0.62 | -1.07 | 1.90 | 1.90 | 1.90 | 2.414 | 6 | 1.34 |
| CuOs | 1.17 | 1.26 | 0.62 | 2.32 | 1.90 | 2.2 | 2.0 | 2.393 | 6 | 1.247 |
| CuIr | 1.17 | 1.27 | 0.62 | 1.2 | 1.90 | 2.2 | 2.0 | 2.338 | 6 | 1.24 |
| CuPt | 1.17 | 1.3 | 0.62 | 2.2 | 1.90 | 2.28 | 2.09 | 2.706 | 6 | 1.243 |
| CuAu | 1.17 | 1.34 | 0.62 | 5.22 | 1.90 | 2.54 | 2.22 | 2.735 | 6 | 1.251 |
| Zn_2_ | 1.25 | 1.25 | -1.08 | -1.08 | 1.65 | 1.65 | 1.65 | 2.652 | 6 | 1.323 |
| ZnZr | 1.25 | 1.45 | -1.08 | -6.46 | 1.65 | 1.33 | 1.49 | 2.696 | 6 | 1.316 |
| ZnNb | 1.25 | 1.34 | -1.08 | -8.76 | 1.65 | 1.6 | 1.62 | 2.577 | 6 | 1.326 |
| ZnMo | 1.25 | 1.30 | -1.08 | -1.4 | 1.65 | 2.16 | 1.90 | 2.479 | 6 | 1.415 |
| ZnRu | 1.25 | 1.25 | -1.08 | 0.61 | 1.65 | 2.2 | 1.92 | 2.417 | 6 | 1.418 |
| ZnRh | 1.25 | 1.25 | -1.08 | 1.32 | 1.65 | 2.28 | 1.96 | 2.522 | 6 | 1.345 |
| ZnPd | 1.25 | 1.28 | -1.08 | 0.92 | 1.65 | 2.2 | 1.92 | 2.693 | 6 | 1.246 |
| ZnAg | 1.25 | 1.34 | -1.08 | 1.60 | 1.65 | 1.93 | 1.79 | 2.573 | 6 | 1.256 |
| ZnHf | 1.25 | 1.44 | -1.08 | -7.08 | 1.65 | 1.3 | 1.48 | 2.573 | 6 | 1.432 |
| ZnTa | 1.25 | 1.34 | -1.08 | -10.93 | 1.65 | 1.5 | 1.58 | 2.66 | 6 | 1.502 |
| ZnW | 1.25 | 1.3 | -1.08 | -1.18 | 1.65 | 2.36 | 2.00 | 2.699 | 6 | 1.436 |
| ZnRe | 1.25 | 1.28 | -1.08 | -1.07 | 1.65 | 1.9 | 1.77 | 2.44 | 6 | 1.453 |
| ZnOs | 1.25 | 1.26 | -1.08 | 2.32 | 1.65 | 2.2 | 1.92 | 2.447 | 6 | 1.43 |
| ZnIr | 1.25 | 1.27 | -1.08 | 1.2 | 1.65 | 2.2 | 1.92 | 2.524 | 6 | 1.31 |
| ZnPt | 1.25 | 1.3 | -1.08 | 2.2 | 1.65 | 2.28 | 1.96 | 2.715 | 6 | 1.29 |
| ZnAu | 1.25 | 1.34 | -1.08 | 5.22 | 1.65 | 2.54 | 2.10 | 2.755 | 6 | 1.311 |

**Table S7** Raw data set of nine eigenvalues of 105 M_1_M_2_-2 DACs. The systems that can form side-on adsorption with O_2_ are marked in blue.

|  | R_M1_ | R_M2_ | E_M1_ | E_M2_ | N_M1_ | N_M2_ | Mean-N_M1/M2_ | Bond_M12_ | N_num_ | L_O2_ |
| --- | --- | --- | --- | --- | --- | --- | --- | --- | --- | --- |
| Mn_2_ | 1.17 | 1.17 | -4.37 | -4.37 | 1.55 | 1.55 | 1.55 | 3.48 | 7 | 1.378 |
| MnFe | 1.17 | 1.17 | -4.37 | -2.02 | 1.55 | 1.83 | 1.69 | 3.476 | 7 | 1.374 |
| MnCo | 1.17 | 1.16 | -4.37 | -0.31 | 1.55 | 1.88 | 1.72 | 3.53 | 7 | 1.329 |
| MnNi | 1.17 | 1.15 | -4.37 | 0.43 | 1.55 | 1.91 | 1.73 | 3.53 | 7 | 1.295 |
| MnCu | 1.17 | 1.17 | -4.37 | 0.62 | 1.55 | 1.9 | 1.72 | 3.486 | 7 | 1.294 |
| MnZr | 1.17 | 1.45 | -4.37 | -6.46 | 1.55 | 1.33 | 1.44 | 3.21 | 7 | 1.496 |
| MnNb | 1.17 | 1.34 | -4.37 | -8.76 | 1.55 | 1.6 | 1.58 | 3.158 | 7 | 1.453 |
| MnMo | 1.17 | 1.30 | -4.37 | -1.4 | 1.55 | 2.16 | 1.86 | 3.388 | 7 | 1.41 |
| MnRu | 1.17 | 1.25 | -4.37 | 0.61 | 1.55 | 2.2 | 1.88 | 3.417 | 7 | 1.398 |
| MnRh | 1.17 | 1.25 | -4.37 | 1.32 | 1.55 | 2.28 | 1.92 | 3.488 | 7 | 1.364 |
| MnPd | 1.17 | 1.28 | -4.37 | 0.92 | 1.55 | 2.2 | 1.88 | 3.561 | 7 | 1.291 |
| MnAg | 1.17 | 1.34 | -4.37 | 1.60 | 1.55 | 1.93 | 1.74 | 3.476 | 7 | 1.309 |
| MnHf | 1.17 | 1.44 | -4.37 | -7.08 | 1.55 | 1.3 | 1.42 | 3.352 | 7 | 1.511 |
| MnTa | 1.17 | 1.34 | -4.37 | -10.93 | 1.55 | 1.5 | 1.52 | 3.546 | 7 | 1.462 |
| MnW | 1.17 | 1.3 | -4.37 | -1.18 | 1.55 | 2.36 | 1.96 | 3.292 | 7 | 1.442 |
| MnRe | 1.17 | 1.28 | -4.37 | -1.07 | 1.55 | 1.9 | 1.72 | 3.312 | 7 | 1.431 |
| MnOs | 1.17 | 1.26 | -4.37 | 2.32 | 1.55 | 2.2 | 1.88 | 3.418 | 7 | 1.424 |
| MnIr | 1.17 | 1.27 | -4.37 | 1.2 | 1.55 | 2.2 | 1.88 | 3.487 | 7 | 1.382 |
| MnPt | 1.17 | 1.3 | -4.37 | 2.2 | 1.55 | 2.28 | 1.92 | 3.566 | 7 | 1.315 |
| MnAu | 1.17 | 1.34 | -4.37 | 5.22 | 1.55 | 2.54 | 2.04 | 3.602 | 7 | 1.306 |
| Fe_2_ | 1.17 | 1.17 | -2.02 | -2.02 | 1.83 | 1.83 | 1.83 | 3.555 | 7 | 1.34 |
| FeCo | 1.17 | 1.16 | -2.02 | -0.31 | 1.83 | 1.88 | 1.86 | 3.55 | 7 | 1.352 |
| FeNi | 1.17 | 1.15 | -2.02 | 0.43 | 1.83 | 1.91 | 1.87 | 3.551 | 7 | 1.296 |
| FeCu | 1.17 | 1.17 | -2.02 | 0.62 | 1.83 | 1.9 | 1.87 | 3.552 | 7 | 1.296 |
| FeZr | 1.17 | 1.45 | -2.02 | -6.46 | 1.83 | 1.33 | 1.58 | 3.276 | 7 | 1.492 |
| FeNb | 1.17 | 1.34 | -2.02 | -8.76 | 1.83 | 1.6 | 1.72 | 3.196 | 7 | 1.52 |
| FeMo | 1.17 | 1.3 | -2.02 | -1.4 | 1.83 | 2.16 | 2.00 | 3.493 | 7 | 1.51 |
| FeRu | 1.17 | 1.25 | -2.02 | 0.61 | 1.83 | 2.2 | 2.02 | 3.502 | 7 | 1.417 |
| FeRh | 1.17 | 1.25 | -2.02 | 1.32 | 1.83 | 2.28 | 2.06 | 3.545 | 7 | 1.366 |
| FePd | 1.17 | 1.28 | -2.02 | 0.92 | 1.83 | 2.2 | 2.02 | 3.576 | 7 | 1.293 |
| FeAg | 1.17 | 1.34 | -2.02 | 1.6 | 1.83 | 1.93 | 1.88 | 3.518 | 7 | 1.297 |
| FeHf | 1.17 | 1.44 | -2.02 | -7.08 | 1.83 | 1.3 | 1.56 | 3.319 | 7 | 1.505 |
| FeTa | 1.17 | 1.34 | -2.02 | -10.93 | 1.83 | 1.5 | 1.66 | 3.282 | 7 | 1.51 |
| FeW | 1.17 | 1.30 | -2.02 | -1.18 | 1.83 | 2.36 | 2.10 | 3.314 | 7 | 1.53 |
| FeRe | 1.17 | 1.28 | -2.02 | -1.07 | 1.83 | 1.9 | 1.86 | 3.466 | 7 | 1.48 |
| FeOs | 1.17 | 1.26 | -2.02 | 2.32 | 1.83 | 2.2 | 2.02 | 3.48 | 7 | 1.48 |
| FeIr | 1.17 | 1.27 | -2.02 | 1.2 | 1.83 | 2.2 | 2.02 | 3.556 | 7 | 1.391 |
| FePt | 1.17 | 1.30 | -2.02 | 2.2 | 1.83 | 2.28 | 2.05 | 3.581 | 7 | 1.298 |
| FeAu | 1.17 | 1.34 | -2.02 | 5.22 | 1.83 | 2.54 | 2.18 | 3.569 | 7 | 1.318 |
| Co_2_ | 1.16 | 1.16 | -0.31 | -0.31 | 1.88 | 1.88 | 1.88 | 3.545 | 7 | 1.275 |
| CoNi | 1.16 | 1.15 | -0.31 | 0.43 | 1.88 | 1.91 | 1.90 | 3.514 | 7 | 1.288 |
| CoCu | 1.16 | 1.17 | -0.31 | 0.62 | 1.88 | 1.90 | 1.89 | 3.549 | 7 | 1.275 |
| CoZr | 1.16 | 1.45 | -0.31 | -6.46 | 1.88 | 1.33 | 1.60 | 3.296 | 7 | 1.434 |
| CoNb | 1.16 | 1.34 | -0.31 | -8.76 | 1.88 | 1.60 | 1.74 | 3.304 | 7 | 1.29 |
| CoMo | 1.16 | 1.30 | -0.31 | -1.4 | 1.88 | 2.16 | 2.02 | 3.372 | 7 | 1.51 |
| CoRu | 1.16 | 1.25 | -0.31 | 0.61 | 1.88 | 2.2 | 2.04 | 3.531 | 7 | 1.375 |
| CoRh | 1.16 | 1.25 | -0.31 | 1.32 | 1.88 | 2.28 | 2.08 | 3.544 | 7 | 1.353 |
| CoPd | 1.16 | 1.28 | -0.31 | 0.92 | 1.88 | 2.2 | 2.04 | 3.572 | 7 | 1.336 |
| CoAg | 1.16 | 1.34 | -0.31 | 1.6 | 1.88 | 1.93 | 1.90 | 3.533 | 7 | 1.251 |
| CoHf | 1.16 | 1.44 | -0.31 | -7.08 | 1.88 | 1.3 | 1.59 | 3.341 | 7 | 1.448 |
| CoTa | 1.16 | 1.34 | -0.31 | -10.93 | 1.88 | 1.5 | 1.69 | 3.296 | 7 | 1.456 |
| CoW | 1.16 | 1.3 | -0.31 | -1.18 | 1.88 | 2.36 | 2.12 | 3.311 | 7 | 1.509 |
| CoRe | 1.16 | 1.28 | -0.31 | -1.07 | 1.88 | 1.9 | 1.89 | 3.491 | 7 | 1.48 |
| CoOs | 1.16 | 1.26 | -0.31 | 2.32 | 1.88 | 2.2 | 2.04 | 3.532 | 7 | 1.382 |
| CoIr | 1.16 | 1.27 | -0.31 | 1.2 | 1.88 | 2.2 | 2.04 | 3.547 | 7 | 1.348 |
| CoPt | 1.16 | 1.30 | -0.31 | 2.2 | 1.88 | 2.28 | 2.08 | 3.575 | 7 | 1.286 |
| CoAu | 1.16 | 1.34 | -0.31 | 5.22 | 1.88 | 2.54 | 2.21 | 3.54 | 7 | 1.27 |
| Ni_2_ | 1.15 | 1.15 | 0.43 | 0.43 | 1.91 | 1.91 | 1.91 | 3.516 | 7 | 1.246 |
| NiCu | 1.15 | 1.17 | 0.43 | 0.62 | 1.91 | 1.9 | 1.90 | 3.416 | 7 | 1.247 |
| NiZr | 1.15 | 1.45 | 0.43 | -6.46 | 1.91 | 1.33 | 1.62 | 3.39 | 7 | 1.494 |
| NiNb | 1.15 | 1.34 | 0.43 | -8.76 | 1.91 | 1.6 | 1.76 | 3.407 | 7 | 1.318 |
| NiMo | 1.15 | 1.3 | 0.43 | -1.4 | 1.91 | 2.16 | 2.04 | 3.55 | 7 | 1.311 |
| NiRu | 1.15 | 1.25 | 0.43 | 0.61 | 1.91 | 2.2 | 2.06 | 3.539 | 7 | 1.302 |
| NiRh | 1.15 | 1.25 | 0.43 | 1.32 | 1.91 | 2.28 | 2.10 | 3.535 | 7 | 1.286 |
| NiPd | 1.15 | 1.28 | 0.43 | 0.92 | 1.91 | 2.2 | 2.06 | 3.574 | 7 | 1.244 |
| NiAg | 1.15 | 1.34 | 0.43 | 1.6 | 1.91 | 1.93 | 1.92 | 3.533 | 7 | 1.256 |
| NiHf | 1.15 | 1.44 | 0.43 | -7.08 | 1.91 | 1.3 | 1.60 | 3.43 | 7 | 1.504 |
| NiTa | 1.15 | 1.34 | 0.43 | -10.93 | 1.91 | 1.5 | 1.70 | 3.374 | 7 | 1.451 |
| NiW | 1.15 | 1.3 | 0.43 | -1.18 | 1.91 | 2.36 | 2.14 | 3.584 | 7 | 1.319 |
| NiRe | 1.15 | 1.28 | 0.43 | -1.07 | 1.91 | 1.90 | 1.90 | 3.556 | 7 | 1.312 |
| NiOs | 1.15 | 1.26 | 0.43 | 2.32 | 1.91 | 2.2 | 2.06 | 3.53 | 7 | 1.312 |
| NiIr | 1.15 | 1.27 | 0.43 | 1.2 | 1.91 | 2.2 | 2.06 | 3.54 | 7 | 1.294 |
| NiPt | 1.15 | 1.3 | 0.43 | 2.2 | 1.91 | 2.28 | 2.09 | 3.571 | 7 | 1.245 |
| NiAu | 1.15 | 1.34 | 0.43 | 5.22 | 1.91 | 2.54 | 2.22 | 3.572 | 7 | 1.25 |
| Cu_2_ | 1.17 | 1.17 | 0.62 | 0.62 | 1.90 | 1.90 | 1.90 | 3.518 | 7 | 1.251 |
| CuZr | 1.17 | 1.45 | 0.62 | -6.46 | 1.90 | 1.33 | 1.62 | 3.277 | 7 | 1.23 |
| CuNb | 1.17 | 1.34 | 0.62 | -8.76 | 1.90 | 1.60 | 1.75 | 3.584 | 7 | 1.245 |
| CuMo | 1.17 | 1.3 | 0.62 | -1.4 | 1.90 | 2.16 | 2.03 | 3.538 | 7 | 1.26 |
| CuRu | 1.17 | 1.25 | 0.62 | 0.61 | 1.90 | 2.2 | 2.05 | 3.531 | 7 | 1.307 |
| CuRh | 1.17 | 1.25 | 0.62 | 1.32 | 1.90 | 2.28 | 2.09 | 3.547 | 7 | 1.292 |
| CuPd | 1.17 | 1.28 | 0.62 | 0.92 | 1.90 | 2.2 | 2.05 | 3.579 | 7 | 1.246 |
| CuAg | 1.17 | 1.34 | 0.62 | 1.6 | 1.90 | 1.93 | 1.92 | 3.526 | 7 | 1.261 |
| CuHf | 1.17 | 1.44 | 0.62 | -7.08 | 1.90 | 1.3 | 1.6 | 3.32 | 7 | 1.231 |
| CuW | 1.17 | 1.3 | 0.62 | -1.18 | 1.90 | 2.36 | 2.13 | 3.544 | 7 | 1.243 |
| CuRe | 1.17 | 1.28 | 0.62 | -1.07 | 1.90 | 1.90 | 1.90 | 3.527 | 7 | 1.313 |
| CuOs | 1.17 | 1.26 | 0.62 | 2.32 | 1.90 | 2.2 | 2.0 | 3.517 | 7 | 1.325 |
| CuIr | 1.17 | 1.27 | 0.62 | 1.2 | 1.90 | 2.2 | 2.0 | 3.555 | 7 | 1.299 |
| CuPt | 1.17 | 1.3 | 0.62 | 2.2 | 1.90 | 2.28 | 2.09 | 3.579 | 7 | 1.25 |
| CuAu | 1.17 | 1.34 | 0.62 | 5.22 | 1.90 | 2.54 | 2.22 | 3.568 | 7 | 1.253 |
| Zn_2_ | 1.25 | 1.25 | -1.08 | -1.08 | 1.65 | 1.65 | 1.65 | 3.491 | 7 | 1.251 |
| ZnZr | 1.25 | 1.45 | -1.08 | -6.46 | 1.65 | 1.33 | 1.49 | 3.488 | 7 | 1.24 |
| ZnNb | 1.25 | 1.34 | -1.08 | -8.76 | 1.65 | 1.6 | 1.62 | 3.368 | 7 | 1.28 |
| ZnMo | 1.25 | 1.30 | -1.08 | -1.4 | 1.65 | 2.16 | 1.90 | 3.278 | 7 | 1.251 |
| ZnRu | 1.25 | 1.25 | -1.08 | 0.61 | 1.65 | 2.2 | 1.92 | 3.496 | 7 | 1.368 |
| ZnRh | 1.25 | 1.25 | -1.08 | 1.32 | 1.65 | 2.28 | 1.96 | 3.545 | 7 | 1.347 |
| ZnPd | 1.25 | 1.28 | -1.08 | 0.92 | 1.65 | 2.2 | 1.92 | 3.565 | 7 | 1.289 |
| ZnAg | 1.25 | 1.34 | -1.08 | 1.60 | 1.65 | 1.93 | 1.79 | 3.533 | 7 | 1.28 |
| ZnHf | 1.25 | 1.44 | -1.08 | -7.08 | 1.65 | 1.3 | 1.48 | 3.531 | 7 | 1.261 |
| ZnTa | 1.25 | 1.34 | -1.08 | -10.93 | 1.65 | 1.5 | 1.58 | 3.511 | 7 | 1.245 |
| ZnW | 1.25 | 1.3 | -1.08 | -1.18 | 1.65 | 2.36 | 2.00 | 3.517 | 7 | 1.23 |
| ZnRe | 1.25 | 1.28 | -1.08 | -1.07 | 1.65 | 1.9 | 1.77 | 3.552 | 7 | 1.407 |
| ZnOs | 1.25 | 1.26 | -1.08 | 2.32 | 1.65 | 2.2 | 1.92 | 3.533 | 7 | 1.392 |
| ZnIr | 1.25 | 1.27 | -1.08 | 1.2 | 1.65 | 2.2 | 1.92 | 3.556 | 7 | 1.362 |
| ZnPt | 1.25 | 1.3 | -1.08 | 2.2 | 1.65 | 2.28 | 1.96 | 3.58 | 7 | 1.289 |
| ZnAu | 1.25 | 1.34 | -1.08 | 5.22 | 1.65 | 2.54 | 2.10 | 3.483 | 7 | 1.291 |

**Table S8.** The E_anti-agg_ and U_diss_ of 78 DACs can follow the O-O dissociation mechanism. The unstable systems are marked in red.

|  | **E_anti-agg_ /eV** | **U_diss_ /eV** |  | **E_anti-agg_ /eV** | **U_diss_ /eV** |
| --- | --- | --- | --- | --- | --- |
| Mn_2_-1 | -3.28 | 0.46 | FeRu-2 | -0.54 | 0.06 |
| MnFe-1 | -1.97 | 0.54 | FeRh-2 | -1.58 | 0.40 |
| MnCo-1 | -2.44 | 0.94 | CoRh-2 | -2.32 | 1.13 |
| Fe_2_-1 | -1.33 | 0.22 | MnRe-2 | 0.60 | 0.56 |
| FeCo-1 | -2.28 | 0.69 | MnOs-2 | -0.37 | 0.43 |
| Co_2_-1 | -2.43 | 0.93 | MnIr-2 | -1.46 | 0.78 |
| MnRu-1 | -2.40 | 0.53 | FeRe-2 | 0.28 | 0.73 |
| MnRh-1 | -2.42 | 0.64 | FeOs-2 | -0.02 | -0.08 |
| FeRu-1 | -0.82 | -0.04 | FeW-2 | -0.01 | -0.44 |
| FeRh-1 | -1.56 | 0.33 | FeIr-2 | -1.82 | 0.62 |
| MnRe-1 | -1.70 | 0.41 | CoRe-2 | 0.51 | -0.97 |
| MnOs-1 | 0.51 | -0.01 | CoOs-2 | -0.94 | 0.74 |
| MnIr-1 | 0.13 | 0.54 | CoIr-2 | -2.04 | 1.10 |
| FeRe-1 | 4.11 | 0.85 | MnNb-2 | -1.14 | -0.72 |
| FeOs-1 | -1.08 | 0.10 | MnTa-2 | -1.35 | -0.15 |
| FeIr-1 | -0.71 | -0.10 | MnW-2 | 0.38 | -2.04 |
| CoRe-1 | -1.72 | 0.58 | FeZr-2 | -2.60 | 0.29 |
| MnZr-1 | -2.50 | -0.27 | FeNb-2 | -0.31 | -0.53 |
| FeZr-1 | -0.51 | -0.78 | FeMo-2 | -0.43 | 0.01 |
| FeMo-1 | -0.92 | 0.01 | FeTa-2 | 0.33 | 0.34 |
| FeTa-1 | -1.11 | 0.11 | CoZr-2 | -0.72 | -0.75 |
| FeW-1 | 5.12 | -0.84 | CoMo-2 | -0.78 | 0.06 |
| CoZr-1 | -1.33 | -0.61 | CoHf-2 | -2.94 | -0.81 |
| CoW-1 | -1.50 | 0.47 | CoTa-2 | -0.83 | 0.01 |
| NiMo-1 | -1.60 | 0.54 | CoW-2 | 0.02 | -0.64 |
| CuZr-1 | -0.49 | -0.62 | NiTa-2 | -1.25 | -0.06 |
| CuNb-1 | -0.34 | -0.22 | CoRu-2 | -0.92 | 0.61 |
| CoZn-1 | -2.44 | 0.94 | CoZn-2 | -2.50 | 0.97 |
| MnZn-1 | -3.95 | 0.79 | MnZn-2 | -3.91 | 0.77 |
| FeZn-1 | -2.00 | 0.56 | FeZn-2 | -1.91 | 0.51 |
| Zn_2_-1 | -3.44 | 0.96 | ZnRu-2 | -0.63 | 0.77 |
| ZnRu-1 | -2.10 | 0.58 | ZnRh-2 | -1.42 | 1.15 |
| ZnRh-1 | -1.83 | 0.55 | ZeRe-2 | 0.03 | 1.31 |
| ZnRe-1 | -1.75 | 0.64 | ZnOs-2 | 0.73 | 1.10 |
| ZnOs-1 | -1.98 | 0.39 | ZnIr-2 | -1.72 | 1.40 |
| Mn_2_-2 | -3.25 | 0.44 | FeCo-2 | -2.17 | 0.64 |
| MnFe-2 | -1.47 | 0.29 | Co_2_-2 | -2.78 | 1.11 |
| MnCo-2 | -2.29 | 0.84 | MnRu-2 | -0.36 | 0.30 |
| Fe_2_-2 | -1.94 | 0.52 | MnRh-2 | -1.33 | 0.61 |

**Table S9.** Standard dissolution potential U^0^_diss_ and the number of transferred electrons *n* involved in dissolution.

|  | **U^0^_diss_ (V)** | ***n*** |
| --- | --- | --- |
| Fe | -0.447 | 2 |
| Co | -0.28 | 2 |
| Mn | -1.185 | 2 |
| Ni | -0.257 | 2 |
| Cu | 0.342 | 2 |
| Ru | 0.455 | 2 |
| Rh | 0.6 | 2 |
| Re | 0.3 | 3 |
| Os | 0.85 | 2 |
| Ir | 1.156 | 3 |
| Zr | -1.553 | 4 |
| Mo | -0.2 | 3 |
| Ta | -0.6 | 3 |
| Nb | -1.099 | 3 |
| W | 0.1 | 3 |
| Hf | -1.55 | 4 |

**Table S10.** Adsorption energy values of reactants ( Δ*G*_O+OH*_, Δ*G*_OOH*_, Δ*G*_O*_, Δ*G*_2OH*_ and Δ*G*_OH*_, in unit of eV) during the ORR process for M_1_M_2_-1 DACs when using the constant charge calculation method.

| **System** | **Δ*G*_O+OH*_** | **Δ*G*_2OH*_** | **Δ*G*_OOH*_** | **Δ*G*_O*_** | **Δ*G*_OH*_** |
| --- | --- | --- | --- | --- | --- |
| Mn_2_ | 3.48 | 1.78 | 3.53 | 2.44 | 0.61 |
| MnFe | 3.48 | 1.89 | 3.95 | 2.37 | 0.83 |
| MnCo | 3.60 | 1.98 | 3.80 | 2.43 | 0.72 |
| MnRh | 3.49 | 2.49 | 4.26 | 2.02 | 0.87 |
| Fe_2_ | 3.80 | 1.92 | 3.38 | 1.44 | 0.79 |
| FeCo | 3.49 | 2.11 | 4.25 | 2.10 | 1.19 |
| FeRh | 3.44 | 1.94 | 3.94 | 2.27 | 0.88 |
| Co_2_ | 3.83 | 2.36 | 4.04 | 2.67 | 1.12 |
| CoZn | 3.31 | 2.01 | 4.02 | 3.31 | 0.95 |

**Table S11.** Adsorption energy values of reactants ( Δ*G*_O+OH*_, Δ*G*_OOH*_, Δ*G*_O*_, Δ*G*_2OH*_ and Δ*G*_OH*_, in unit of eV) during the ORR process for M_1_M_2_-2 DACs when using the constant charge calculation method.

| **System** | **Δ*G*_O+OH*_** | **Δ*G*_2OH*_** | **Δ*G*_OOH*_** | **Δ*G*_O*_** | **Δ*G*_OH*_** |
| --- | --- | --- | --- | --- | --- |
| Mn_2_ | 3.26 | 1.83 | 3.87 | 2.36 | 0.72 |
| MnFe | 3.11 | 1.79 | 3.71 | 2.27 | 0.59 |
| MnCo | 2.74 | 2.09 | 3.67 | 1.83 | 0.61 |
| MnRu | 2.50 | 1.91 | 2.84 | 0.86 | 0.36 |
| MnRh | 3.12 | 1.66 | 4.01 | 2.20 | 0.64 |
| MnIr | 2.88 | 1.84 | 3.80 | 1.90 | 0.80 |
| Fe_2_ | 3.20 | 1.94 | 3.79 | 1.96 | 0.76 |
| FeCo | 3.14 | 1.97 | 4.37 | 2.07 | 0.71 |
| FeRu | 3.65 | 2.30 | 3.29 | 1.53 | 1.04 |
| FeRh | 3.05 | 1.92 | 3.53 | 2.11 | 0.88 |
| FeIr | 3.12 | 1.85 | 3.72 | 1.87 | 0.94 |
| FeMo | 3.42 | 2.17 | 3.72 | 1.07 | 0.68 |
| FeZn | 3.06 | 1.70 | 3.96 | 2.19 | 1.00 |
| Co_2_ | 3.30 | 2.21 | 4.00 | 2.75 | 1.05 |
| CoRu | 2.91 | 1.96 | 4.28 | 1.90 | 0.92 |
| CoRh | 3.11 | 2.05 | 3.93 | 2.41 | 1.03 |
| CoZn | 3.65 | 2.01 | 4.04 | 2.87 | 1.07 |
| CoIr | 3.10 | 2.05 | 3.95 | 2.07 | 1.10 |
| CoMo | 3.01 | 1.58 | 3.21 | 0.58 | 0.70 |
| ZnRh | 3.94 | 1.39 | 4.31 | 3.54 | 0.68 |

**Table S12.** According to this predictive model, pH = 1, the predicted activity of ORR is superior to that of the candidate system of FeP-2.

| **System** | **System** |
| --- | --- |
| ZnS-2 | MnSe-2 |
| FeIn-1 | CoP-2 |
| FeTi-1 | FeS-2 |
| FeSn-1 | FeSe-2 |
| FeV-1 | CoS-1 |
| MnS-2 | CoS-2 |
| ZnSe-2 | CoSe-2 |

**Table S13.** According to this predictive model, pH = 13, the predicted activity of ORR is superior to that of the candidate system of FeSn-1.

| **System** | **System** |
| --- | --- |
| FeV-1 | FeSe-2 |
| MnS-2 | CoS-2 |
| ZnSe-2 | CoS-2 |

**Table S14.** Comparison of DFT-optimized M_1_M_2_-1 bond lengths with experimental values. Corresponding to the precisely fabricated N_2_-Fe-N_2_-M-N_2_ active site (Ref. 25^26^).

| **System** | **DFT-optimized/Å** | **Experimental value/Å** |
| --- | --- | --- |
| FeMn-1 | 2.40 | 2.38 |
| Fe_2_-1 | 2.33 | 2.33 |
| FeCo-1 | 2.38 | 2.35 |
| FeNi-1 | 2.49 | 2.41 |
| FeCu-1 | 2.53 | 2.44 |

**Table S15.** The values of U-J parameters for DFT/PBE+U calculations

| **3d** | **Sc** | **Ti** | **V** | **Cr** | **Mn** | **Fe** | **Co** | **Ni** | **Cu** | **Zn** |
| --- | --- | --- | --- | --- | --- | --- | --- | --- | --- | --- |
| **U - J** | 2.11 | 2.58 | 2.72 | 2.79 | 3.06 | 3.29 | 3.42 | 3.4 | 3.87 | 4.12 |

**Table S16.** The average Δ*E*_solv_ values of MnFe-1/CoIr-2 calculated by implicit and explcit models.

|  | **FeMn-1** | | **CoIr-2** | |
| --- | --- | --- | --- | --- |
|  | **Δ*E*_solv-expl_** | **Δ*E*_solv-impl_** | **Δ*E*_solv-expl_** | **Δ*E*_solv-impl_** |
| OOH* | -0.24 | -0.20 | -0.31 | -0.21 |
| O*+OH* | -0.71 | -0.80 | -0.45 | -0.45 |

**References**

1. Furthmuller, G. K. a. J., Efficiency of ab-initio total energy calculations for metals and semiconductors using a plane-wave basis set. *Phys. Rev. B: Condens. Matter Mater. Phys.* **1996,** (54), 11169–11186.

2. ochl, P. E. B., Projector augmented-wave method. *Phys. Rev. B: Condens. Matter Mater. Phys.* **1994,** *50*, 17953–17979.

3. Rostgaard, C., The Projector Augmented-wave Method. **2009,** *62*, 11556–11570.

4. Cui, P.; Zhao, L.; Long, Y.; Dai, L.; Hu, C., Carbon-Based Electrocatalysts for Acidic Oxygen Reduction Reaction. *Angew Chem Int Ed Engl* **2023,** *62* (14), e202218269.

5. Kim, J. H.; Shin, D.; Kim, J.; Lim, J. S.; Paidi, V. K.; Shin, T. J.; Jeong, H. Y.; Lee, K. S.; Kim, H.; Joo, S. H., Reversible Ligand Exchange in Atomically Dispersed Catalysts for Modulating the Activity and Selectivity of the Oxygen Reduction Reaction. *Angew Chem Int Ed Engl* **2021,** *60* (37), 20528-20534.

6. Liu, F.; Shi, L.; Song, S.; Ge, K.; Zhang, X.; Guo, Y.; Liu, D., Simultaneously Engineering the Coordination Environment and Pore Architecture of Metal-Organic Framework-Derived Single-Atomic Iron Catalysts for Ultraefficient Oxygen Reduction. *Small* **2021,** *17* (40), e2102425.

7. S. L. Dudarev; G. A. Botton; S. Y. Savrasov; Humphreys, C. J.; Sutton, A. P., Electron-energy-loss spectra and the structural stability of nickel oxide: An LSDA U study. *PHYSICAL REVIEW B* **1998,** *57* (3), 0163-1829.

8. Liu, J. C.; Xiao, H.; Li, J., Constructing High-Loading Single-Atom/Cluster Catalysts via an Electrochemical Potential Window Strategy. *J Am Chem Soc* **2020,** *142* (7), 3375-3383.

9. Mathew, K.; Kolluru, V. S. C.; Mula, S.; Steinmann, S. N.; Hennig, R. G., Implicit self-consistent electrolyte model in plane-wave density-functional theory. *J Chem Phys* **2019,** *151* (23), 234101.

10. Mathew, K.; Sundararaman, R.; Letchworth-Weaver, K.; Arias, T. A.; Hennig, R. G., Implicit solvation model for density-functional study of nanocrystal surfaces and reaction pathways. *J Chem Phys* **2014,** *140* (8), 084106.

11. Hu, X.; Chen, S.; Chen, L.; Tian, Y.; Yao, S.; Lu, Z.; Zhang, X.; Zhou, Z., What is the Real Origin of the Activity of Fe-N-C Electrocatalysts in the O_2_ Reduction Reaction? Critical Roles of Coordinating Pyrrolic N and Axially Adsorbing Species. *J Am Chem Soc* **2022,** *144* (39), 18144-18152.

12. Tripković, V.; Skúlason, E.; Siahrostami, S.; Nørskov, J. K.; Rossmeisl, J., The oxygen reduction reaction mechanism on Pt(111) from density functional theory calculations. *Electrochimica Acta* **2010,** *55* (27), 7975-7981.

13. Rebarchik, M.; Bhandari, S.; Kropp, T.; Mavrikakis, M., How Noninnocent Spectator Species Improve the Oxygen Reduction Activity of Single-Atom Catalysts: Microkinetic Models from First-Principles Calculations. *Acs Catal* **2020,** *10* (16), 9129-9135.

14. Henkelman, G.; Uberuaga, B. P.; Jónsson, H., A climbing image nudged elastic band method for finding saddle points and minimum energy paths. *The Journal of Chemical Physics* **2000,** *113* (22), 9901-9904.

15. Li, P.; Jiang, Y.; Hu, Y.; Men, Y.; Liu, Y.; Cai, W.; Chen, S., Hydrogen bond network connectivity in the electric double layer dominates the kinetic pH effect in hydrogen electrocatalysis on Pt. *Nat Catal* **2022,** *5* (10), 900-911.

16. TRASA'TTI, S.; Heusler；, K. E.; Justice；, J. C.; Nik；, K.; Agar；, J. N.; Bard；, A. J.; Bewick；, A., THE ABSOLUTE ELECTRODE POTENTIAL: AN EXPLANATORY NOTE. *Pure & AppL Chem.* **1986,** *58*, 55—966.

17. Yiran Ying; Xin Luo; Jinli Qiao; Huang, H., Double-Atom Catalysts: “More is Different:” Synergistic Effect and Structural Engineering in Double-Atom Catalysts. *Adv. Funct. Mater* **2021,** *31* (3), 2170015.

18. Xu, L.; Wang, X.; Hu, X.; Wang, Y.; Zhang, C.; Xu, W.; Zhao, W.; Xu, N.; Woo, D.; Yao, H.; Li, X.; Jiang, H.; Huang, M.; Lee, J.; Zeng, X. C.; Han, Z. K., Artificial-intelligence-assisted design principle for developing high-performance single-atom catalysts. *Innovation (Camb)* **2025,** *6* (7), 100911.

19. Hansen, H. A.; Viswanathan, V.; Nørskov, J. K., Unifying Kinetic and Thermodynamic Analysis of 2 e^–^ and 4 e^–^ Reduction of Oxygen on Metal Surfaces. *The Journal of Physical Chemistry C* **2014,** *118* (13), 6706-6718.

20. Hansen, H. A.; Varley, J. B.; Peterson, A. A.; Norskov, J. K., Understanding Trends in the Electrocatalytic Activity of Metals and Enzymes for CO_2_ Reduction to CO. *J Phys Chem Lett* **2013,** *4* (3), 388-92.

21. Jinnouchi, R.; Kodama, K.; Hatanaka, T.; Morimoto, Y., First principles based mean field model for oxygen reduction reaction. *Phys Chem Chem Phys* **2011,** *13* (47), 21070-83.

22. Liu, J.; Xu, H.; Zhu, J.; Cheng, D., Understanding the Pathway Switch of the Oxygen Reduction Reaction from Single- to Double-/Triple-Atom Catalysts: A Dual Channel for Electron Acceptance-Backdonation. *JACS Au* **2023,** *3* (11), 3031-3044.

23. Liu, X.; Wei, Z.; Liu, J.; Tan, W.; Fang, X.; Fang, D.; Wang, X.; Wang, D.; Tang, J.; Fan, X., Oxidization of Al_0.5_Ga_0.5_As(001) surface: The electronic properties. *Appl Surf Sci* **2018,** *436*, 460-466.

24. Cen, W.; Liu, Y.; Wu, Z.; Wang, H.; Weng, X., A theoretic insight into the catalytic activity promotion of CeO_2_ surfaces by Mn doping. *Phys Chem Chem Phys* **2012,** *14* (16), 5769-77.

25. Li, L.; Zhu, J.; Kong, F.; Wang, Y.; Kang, C.; Xu, M.; Du, C.; Yin, G., Tailoring atomic strain environment for high-performance acidic oxygen reduction by Fe-Ru dual atoms communicative effect. *Matter* **2024,** *7* (4), 1517-1532.

26. Liu, L.; Hu, J.; Rao, X.; Zhu, Y.; Li, P.; Chen, S.; Zhang, S., Revealing the dependence of oxygen reduction mechanism and activity on the D-band center difference of Fe-M bimetallic sites. *Applied Catalysis B: Environment and Energy* **2026,** *384*, 126191.
